# Peptide Prenylation Follows Divergent Substrate Engagement Rules

**DOI:** 10.1021/jacs.5c18820

**Published:** 2025-12-02

**Authors:** Mujeeb A. Wakeel, Andrew C. McShan, Vinayak Agarwal

**Affiliations:** † School of Chemistry and Biochemistry, 1372 Georgia Institute of Technology, Atlanta, Georgia 30332, United States; ‡ School of Biological Sciences, Georgia Institute of Technology, Atlanta, Georgia 30332, United States

## Abstract

Lipidation of ribosomally synthesized and post-translationally modified peptides (RiPPs) is a bioactivity conferring modification. In this study, we present evidence that prenylation of lanthipeptides—a widely distributed chemical family of RiPPs—proceeds with lanthipeptide prenyltransferases recognizing the entirety of the peptidic substrate including the N-terminal leader region of the RiPP precursor peptide. This mode of substrate recognition is markedly different from that of prenyltransferases that lipidate other RiPPs such as cyanobactins wherein the leader peptide is not engaged at all. Molecular recognition rules dictate catalysis, and we posit that leader peptide recognition imposes substrate selectivity upon lanthipeptide prenyltransferases and narrows their substrate scope as compared to the leader-free prenylation of cyanobactins which proceeds in a hyperpromiscuous manner.

## Graphical abstract

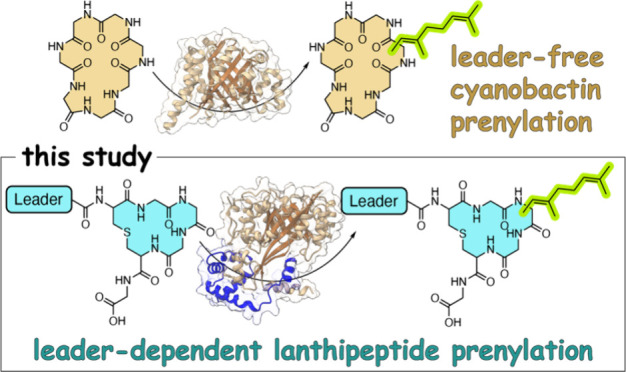

Lipidation of macrocyclic peptides is a pharmaceutically relevant chemical transformation.^1^ Illustratively, lipidation enhances the antibiotic activity of teicoplanin and daptomycin.^2,3^ Lipidation alters the peptide transport properties across biological membranes, an observation that has been exploited for chemical biology as well as therapeutic applications.^4,5^

Among peptidic natural products, macrocyclic ribosomally synthesized and post-translationally derived peptides (RiPPs) combine the pharmaceutical relevance of cyclic peptides with the engineering potential of RiPPs.^6–9^ RiPPs are biosynthesized starting from a precursor peptide. The RiPP biosynthetic enzymes bind to the leader and other recognition sequences (RSs) in the precursor peptide to post-translationally modify the core region. The modified core is proteolytically removed from the leader and RS(s) to furnish the mature RiPP.^10^ This model divests sites for substrate recognition from catalysis; while the leader and the RS(s) are recognized by the RiPP biosynthetic enzymes, post-translational modifications occur only on the core.

Among lipidated macrocyclic RiPPs (lmRiPPs), cyanobactins are the largest chemical family. Deviating from the model of RiPP biosynthesis described above, lipidation in cyanobactin biosynthesis occurs in a leader-independent fashion. Two proteases extract the core region of the cyanobactin precursor peptide from between the N-terminal leader and the C-terminal RS and catalyze N–C macrocyclization (Figure 1A).^11–13^ After the leader and RS have been removed, prenyltransferases (PTs) lipidate the side chains of Ser, Thr, His, Arg, Tyr, and Trp residues of macrocyclic core peptides.^14^ Decoupled from the requirement to engage with the leader peptides or RSs, cyanobactin PTs are hyperpromiscuous and operate with few restrictions on macrocycle sequence and size and can even lipidate structurally different RiPPs from other chemical classes such as lanthipeptides.^14–16^ Even for linear cyanobactins, these PTs operate in a leader-independent fashion.^17^ Leader-independent prenylation of lasso peptides has also been reported.^18^

**Figure 1:**
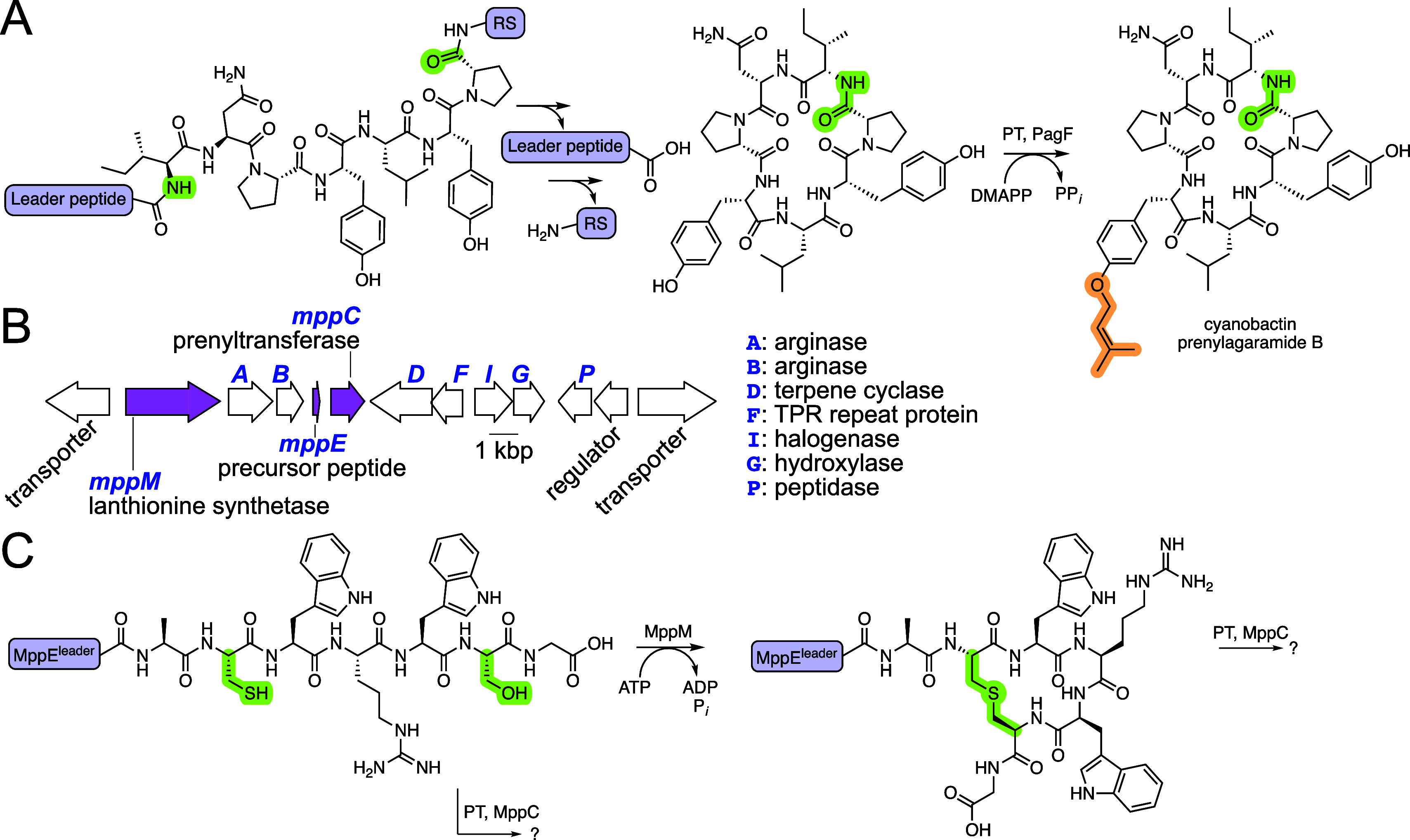
(A) Cyanobactin biosynthesis involves the proteolytic removal of the N-terminal leader and the C-terminal RS to yield a macrocyclic core peptide that is prenylated. PagF is a cyanobactin PT that furnishes the cyanobactin prenylagaramide B. (B) The *mpp* BGC with the *mppE*, *mppM*, and *mppC* genes highlighted in pink. (C) Lanthipeptide biosynthesis wherein the lanthionine synthetase MppM catalyzes macrocyclizing thioether ring formation highlighted in green.

As the macrocyclizing amide bond formation necessitates leader and RS scission from the core peptide, cyanobactin PTs operate in a leader/RS-independent manner which could explain their hyperpromiscuity (Figure 1A). However, numerous other RiPP macrocyclization modalities do not involve leader peptide removal.^19^ Among these, lanthionine ring formation between the Cys and Ser/Thr side chains is well represented; these RiPPs are referred to as lanthipeptides.^20^ This observation prompted us to question whether lanthipeptide prenylation would also proceed in hyperpromiscuous leader-independent manner akin to cyanobactin prenylation. Alternatively, preservation of the leader peptide after lanthionine ring formation could impose a requirement for leader recognition upon lanthipeptide PTs and induce substrate selectivity. Which of these scenarios is operative in lanthipeptide prenylation was not apparent.

As the name suggests, cyanobactins are derived from cyanobacteria. The activities of several cyanobacterial PTs have been described along with their expansive substrate scope.^14,21–25^ To compare the activity of cyanobactin PTs against a cyanobacterial lanthipeptide PT, we chose to characterize the activity of the PT encoded within the cyanobacterium *Moorena producens* PAL-derived *mpp* biosynthetic gene cluster (BGC) (Figure 1B).^26^ Here, the heptapeptide core of the MppE precursor peptide is macrocyclized by the class II lanthionine synthetase MppM (Figure 1C).^27^ Within the *mpp* BGC, the gene *mppC* encoded a α-β-β-α-type soluble PT; cyanobactin PTs reside within the same family.^28^ The RiPP encoded by the *mpp* BGC is cryptic.

First, we queried whether prenylation proceeded on the linear or the MppM-macrocyclized MppE^core^ (Figure 1C, Table S1). By monitoring substrate conversion *in vitro* using purified MppC and C_5_, C_10_, C_15_, and C_20_ prenyl donors—dimethylallyl pyrophosphate (DMAPP), geranyl pyrophosphate (GPP), farnesyl pyrophosphate (FPP), and geranylgeranyl pyrophosphate (GGPP), respectively—we discerned that MppC preferred the macrocyclized MppE^core^ and GPP as substrates (Figure 2A, Figures S1–S13). This observation was in line with a recent report from Chekan and Piel wherein macrocyclization preceded lanthipeptide prenylation.^29^

**Figure 2:**
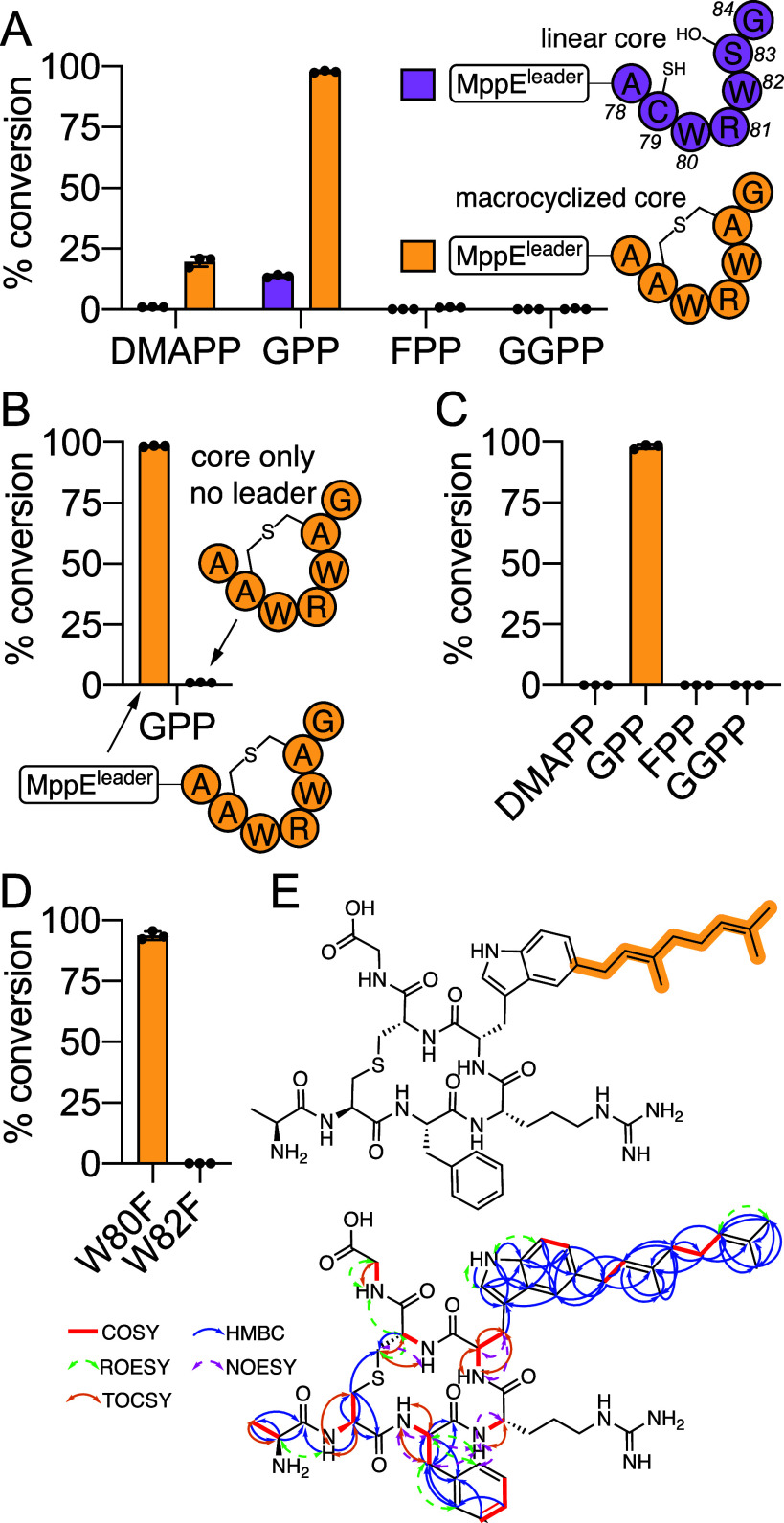
(A) Relative activity of MppC for linear (purple) and macrocyclized MppE precursor peptide (orange). Mean and standard deviation from three independent experiments is plotted with the individual data points shown. (B) Relative activity of MppC for macrocyclized MppE^core^ with and without the presence of the MppE^leader^; GPP was the prenyl donor. (C) Competition experiment in which four prenyl donors were provided together to MppC using macrocyclized MppE precursor peptide. (D) Relative product formation with the Trp80 and Trp82 residues in the MppE^core^ mutated to Phe. (E) Structure (top) and NMR correlations (bottom) for the lmRiPP generated by MppM and MppC; Trp80 mutated to Phe. The lanthionine configuration has been established previously.^27^

To discern the leader dependence of MppC activity, the macrocyclized MppE^core^ was generated with and without the MppE^leader^. Using in vitro assays, we could readily discern substrate conversion was abolished in the absence of the MppE^leader^ or when the MppE^leader^ was present in trans (Figure 2B, Figure S14–S15). Amino acid l-Trp was not accepted as a substrate either (Figure S16). These data juxtapose the leader-dependent activity of MppC against leader-independent cyanobactin PTs discussed above. With the peptide substrate thusly established, preference for the prenyl donor was reconfirmed in a competition experiment wherein all four prenyl donors were provided in the same reaction. Here, we observed accumulation of the geranylated product only (Figure 2C, Figure S17).

Mutation of the Trp82 to Phe abolished geranylated product formation, while product formation was retained for the Trp80Phe mutant indicating that the Trp82 side chain indole would be the site for geranylation (Figure 2D, Figures S18–S22). To minimize spectral overlap between two indole side chains, the geranylated lmRiPP was produced in preparative amounts using the MppE Trp80Phe precursor peptide. Product identity was queried using NMR to reveal that geranylation was affected upon the Trp82 side chain indole-5 position (Figure 2E, Figures S23–S31, Table S2). Mutation of the Trp82 to His and Tyr did not yield any geranylated products (Figures S32–S35).

The obligate leader dependence of MppC prompted us to examine the molecular basis for MppC/MppE^leader^ interaction. The MppE^leader^ is atypically long and belongs to the Nif11-like class of RiPP leader peptides.^30^ Akin to the nitrile hydratase-like leader peptides (NHLPs), the Nif11-like leader peptides are also predicted to possess a structured nucleus (leader^SN^) followed by a disordered region (leader^DR^) (Figure 3A).^31,32^ The Asp/Glu rich leader^DR^ harbors the L­(X)_4_L motif; these attributes are discernible in the MppE sequence. The leader^SN^ and the leader^DR^ in NHLPs adopt independent interaction modalities with RiPP biosynthetic enzymes.^31–34^ The L­(X)_4_L motif is in itself a hotspot for interacting with peptide modifying enzymes.^35–37^

**Figure 3:**
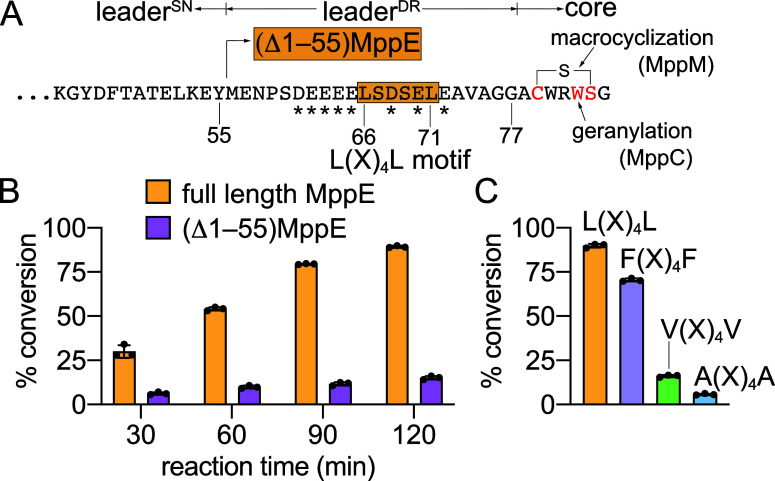
(A) Architecture of the MppE precursor peptide. The boundaries for the leader^SN^, leader^DR^, and the core are marked. The Glu/Asp residues in leader^DR^ are marked by *. The (Δ1–55)­MppE precursor peptide eliminates the leader^SN^. Residues in the MppE^core^ involved in lanthionine ring formation and prenylation are denoted. (B) Relative geranylation activity of MppC for the full-length MppE and the (Δ1–55)­MppE precursor peptides. The two peptides were assayed in vitro as C-terminal fusions to maltose-binding protein (MBP). (C) Prenylation activity of MppC when the L­(X)_4_L motif in the full-length MppE precursor peptide was replaced with F­(X)_4_F, V­(X)_4_V, or A­(X)_4_A.

The (Δ1–55)­MppE construct eliminated the leader^SN^ but preserved the leader^DR^ (Figure 3A). Lanthionine ring in the MppE^core^ was installed by coexpression of the gene encoding the (Δ1–55)­MppE precursor peptide with *mppM* as reported previously.^27^ In vitro assays demonstrated that the (Δ1–55)­MppE construct did not support MppC activity (Figure 3B, Figures S36–S43). Furthermore, mutating the two Leu residues that comprise the L­(X)_4_L motif to Ala and to Val also disrupted MppC activity (Figure 3C, Figures S44–S47). These data allow us to posit that both elements of the MppE^leader^—the leader^SN^ as well as the leader^DR^—are involved in interactions with MppC.

Next, we generated a MppC/MppE^leader^ interaction model using AlphaFold 3 that would satisfy the biochemically imposed constraint that the entirety of the MppE^leader^ should be engaged by MppC. The MppC/MppE^leader^ complex in the presence of a Mg^2+^ was predicted by AlphaFold 3 with excellent statistics (ipTM = 0.83; pTM = 0.89) (Figure 4A, Figure S48). MppC was modeled to possess a central β-barrel with the Mg^2+^ ion bound at the top. The C-terminus of the MppE^leader^ was positioned such that the macrocyclized MppE^core^ would be delivered at the other end of the β-barrel in a manner that would prevent solvent quenching of the allylic carbocation akin to the model for substrate binding described for the cyanobactin prenyltransferase PagF by Nair and Schmidt.^38^ Taken together, substrate binding for MppC modeled by AlphaFold 3 was in line with previously described experimental models of peptide PTs.^15,24,25,38,39^

**Figure 4:**
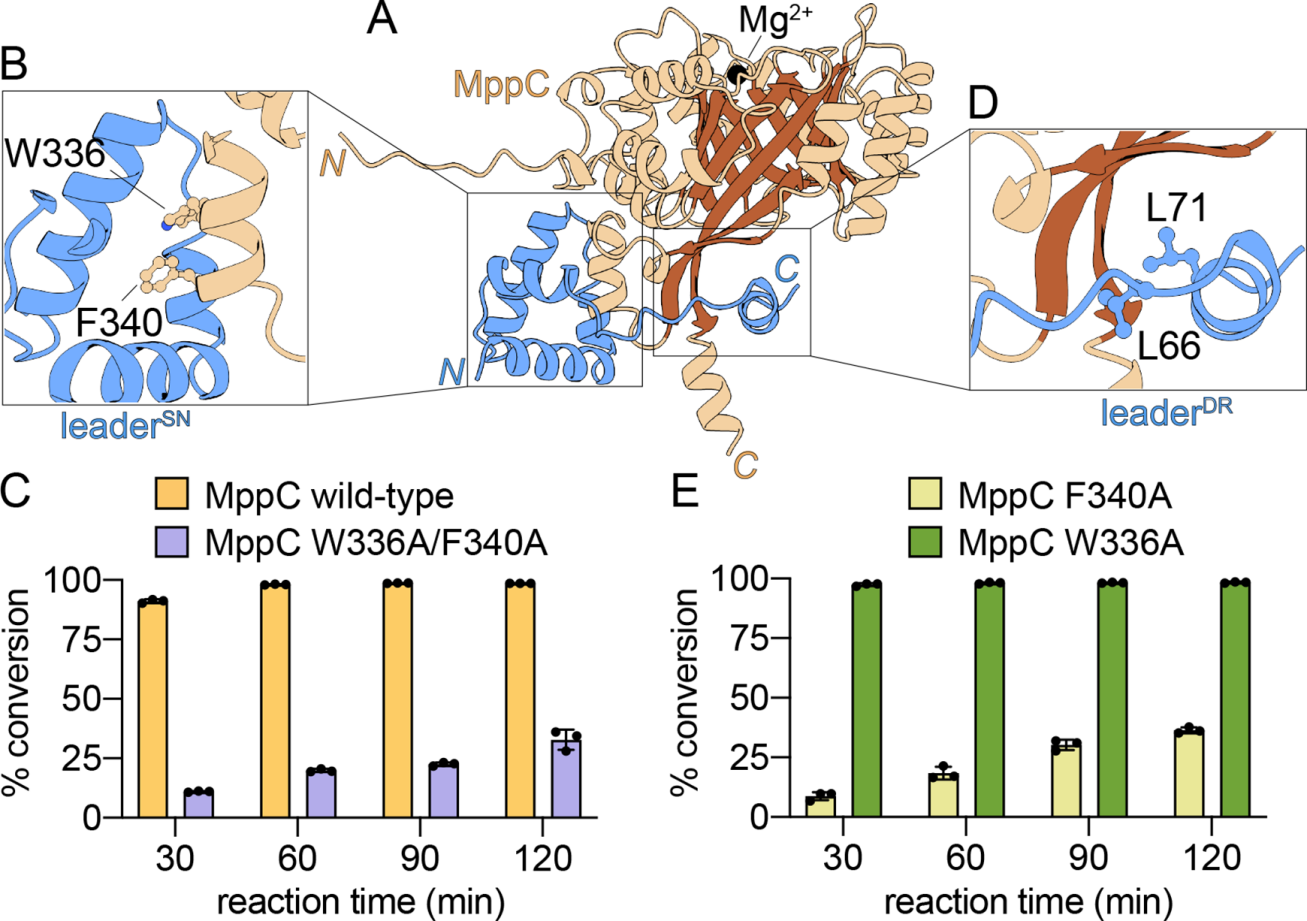
(A) AlphaFold 3-predicted model of MppC in complex with MppE^leader^ and Mg^2+^. MppC is colored brown; MppE^leader^ is colored blue. (B) Zoomed-in view of the MppC Trp336 and Phe340 side chains embedded within the hydrophobic cavity constituted by the rigidly structured nucleus at the MppE^leader^ N-terminus. (C) Prenylation activity of the MppC Trp336Ala/Phe340Ala relative to the wild-type enzyme. (D) Zoomed-in view of the Leu66 and Leu71 side chains that constitute the L­(X)_4_L motif in the intrinsically disordered region of the MppE^leader^ peptide binding to a surface-exposed hydrophobic patch of MppC. (E) Prenylation activity of the MppC Phe340Ala and MppC Trp336Ala enzymes.

The entirety of the MppE^leader^ was engaged by MppC (Figure 4A). The MppC Trp336 and Phe340 side chains were inserted within the hydrophobic cavity of the MppE leader^SN^ (Figure 4B). Mutation of the MppC Trp336 and Phe340 residues to Ala did not cause structural perturbation in MppC itself but led to reduction in the prenylation activity of the mutant enzyme (Figure 4C, Figures S49–S58). Insertion of enzyme hydrophobic side chains in to the leader^SN^ is reminiscent of NHLP binding to class II lanthionine synthetases (LanMs).^31^ The Leu side chains of the MppE L­(X)_4_L motif were modeled to be coordinated to shallow hydrophobic grooves on the MppC surface in a manner reminiscent of the L­(X)_4_L motif binding to peptidase C39 domains (Figure 4D).^37^ Taken together, findings in this study establish that lanthipeptide prenylation follows a different substrate/enzyme interaction paradigm as compared to leader-independent cyanobactin prenylation. Note that N-acylation by fatty acids has also been reported as a lanthipeptide lipidation mechanism by Vagstad and Piel.^40^

To evaluate the individual contributions of the MppC Trp336 and Phe340 residues for binding to the MppE^leader^, single-point mutant MppC enzymes were generated. Curiously, the principal contribution to engaging with the MppE^leader^ was provided by Phe340 alone, and Trp336 had minimal role in this interaction (Figure 4E, Figures S59–S66). The AlphaFold 3-generated model indeed demonstrated that the Phe340 side chain was more intimately entrenched within the hydrophobic cavity of the MppE leader^SN^.

Using MppC as a genome mining hook, putative prenylated lanthipeptide encoding BGCs were identified wherein the PTs were modeled by AlphaFold 3 to follow similar precursor peptide engagement modes as described for MppC in this study (Figures S67–S71).^41^ Most strikingly, other lanthipeptide PTs were modeled to engage the corresponding leader^SN^s using a Phe side chain akin to the role of MppC Phe340 residue described above. Hence, the substrate engagement model described here for MppC might be broadly applicable for leader-dependent lanthipeptide prenylation.

Different modes of peptide substrate binding are directly relevant to catalysis. Among the two Trp residues in the MppE^core^, MppC only prenylates the Trp82 side chain. This observation is in contrast to the activity of the leader-independent cyanobactin prenyltransferases that prenylate multiple side chains within a single substrate.^24,39^ Thus, it is plausible that binding of the MppE^leader^ to MppC sets the register for the highly specific delivery of the macrocyclic MppE^core^ to the MppC active site. Contrasting the highly specific activity of MppC with that of cyanobactin PTs presents an illustration of the physiological role of the leader peptide in imposing specificity for post-translational modification of the core.

## Supplementary Material



## Data Availability

The NMR data reported in this study have been deposited to the NP-MRD database (https://np-mrd.org/) with the accession ID NP0351680.

## Notes

Details regarding materials and methods, Supplementary Tables S1 and S2, Supplementary Figures S1–S71, and supplementary references (PDF)
