# Supplementary material for: Peptide Prenylation Follows Divergent Substrate Engagement Rules
Source: J Am Chem Soc. 2025 Dec 2;147(50):45855–60. doi: 10.1021/jacs.5c18820 (PMC12715784; doi:10.1021/jacs.5c18820)
Supplement: Supplementary file 1 [file ja5c18820_si_001.pdf]

SUPPLEMENTARY INFORMATION FOR:

Peptide prenylation follows divergent substrate engagement rules

Mujeeb A. Wakeel,[1] Andrew C. McShan,[1] Vinayak Agarwal[1,2,]*

[1] School of Chemistry and Biochemistry, Georgia Institute of Technology, USA 30332

[2] School of Biological Sciences, Georgia Institute of Technology, USA 30332

* correspondence: vagarwal@gatech.edu

This Supplementary Information document contains:

Supplementary Materials and Methods

Supplementary Tables

Supplementary Figures

Supplementary References

SUPPLEMENTARY MATERIALS AND METHODS

Preparation of expression plasmids

The *mppC* gene from the *Moorena producens*-derived *mpp* biosynthetic gene cluster was codon-optimized for expression in *Escherichia coli* and synthesized (Twist Biosciences) with insertion into the pET-28a(+) plasmid vector. The pET-28a(+)-*mppE*, pCDFDuet-1-*mppM*, and pET-28a(+)-*mppM* plasmids used here were constructed in a previous study.[1] Codon-optimized genes encoding MppE and (Δ1–55)MppE were amplified from pET-28a(+)-*mppE* using PrimeSTAR Max Premix (2×) DNA polymerase (Takara Bio) for cloning into pET-28a(+)-MBP vector. Purified amplicons encoding MppE and (Δ1–55)MppE were cloned into pET-28a(+)-MBP vector using Gibson assembly method, resulting in pET-28a(+)-MBP-*mppE* and pET-28a(+)-MBP-*(Δ1–55)MppE* (see Table S1 for peptide sequences). The *mppC* and *mppE* mutants were constructed using site-directed mutagenesis with primers obtained from Eton Bioscience. All DNA cloning was performed using *E. coli* DH5α, and 50 μg/mL kanamycin was used to apply negative selection pressure. DNA sequencing of the isolated recombinant plasmids was performed using Sanger sequencing and Oxford Nanopore Technologies (Eton Bioscience and Plasmidsaurus).

Protein expression

Sequencing-verified recombinant plasmids were transformed into *E. coli* BL21(DE3) competent cells using standard heat-shock protocol and grown at 37 °C for 18 h on LB-agar plate containing appropriate antibiotics. 10 mL terrific broth (TB) media (Fisher Scientific) containing appropriate antibiotics was inoculated with selected colonies, followed by incubation in a 37 °C shaker (180 rpm) for 18 h. The 10 mL culture was then used to grow a 1 L culture at 37 °C with constant shaking at 180 rpm while measuring optical density at 600 nm wavelength (OD_{600}). When OD_{600} reached 0.6–0.8, the temperature was reduced to 16 °C. After 45 min of cooling, protein expression was induced with appropriate inducers: 0.15 mM isopropyl-β-D-thiogalactopyranoside (IPTG) for expression of genes inserted in pCDFDuet-1 and pET-28a(+) plasmids and 1 mg/mL L-(+)-arabinose for expression of chaperone genes in the pGro7 plasmid (Takara Bio). Co-expression of pET-28a(+)-*mppE* wild-type and mutants thereof with pCDFDuet-1-*mppM* was performed for *in vivo* macrocyclization of $MppE^{core}$ peptide by MppM lanthionine synthetase. Genes encoding MppC and MppM were separately co-expressed with pGro7 for protein purification of MppC and MppM on Ni^{2+}-charged HisTrap columns (Cytiva) to conduct *in vitro* peptide modifications. Genes were expressed at 16 °C, 180 rpm for 18 h, followed by culture harvest and protein purification.

Protein purification

After 18 h of expression, cultures were centrifuged (3,315×g, 25 min, 4 °C, Avanti®J-E) and *E. coli* cells from 1 L culture were resuspended by vortexing in 40 mL lysis buffer (20 mM Tris-Cl (pH 7.5), 500 mM NaCl, 30 mM imidazole). Resuspended cells were subjected to sonication (QSonica Q500 sonicator: pulse 10 s on, 50 s off; at 50% amplitude for 15 min), and the resulting cell lysate was centrifuged (39,191×g, 45 min, 4 °C, Avanti™J-30I) for clarification and recovery of soluble proteins.

Ni-NTA affinity chromatography:

Clarified supernatant containing soluble proteins was loaded onto a 5 mL Ni^{2+}-charged HisTrap column (Cytiva) pre-equilibrated with 10 column volumes (CV) of binding buffer (20 mM Tris-Cl (pH 7.5), 500 mM NaCl, 30 mM imidazole). The protein-bound Ni^{2+}-column was extensively washed with binding buffer at 2 mL/min flow rate on AKTA Go system (Cytiva), followed by elution of the protein of interest using a linear gradient of elution buffer (20 mM Tris-Cl (pH 7.5), 500 mM NaCl, 1 M imidazole) from 0–100% column volumes over 30 min. Purified protein fractions were collected and analyzed by SDS-PAGE, and fractions containing the desired protein were pooled and concentrated. Concentrated proteins of interest were then buffer-exchanged into 20 mM HEPES-Na (pH 7.5), 500 mM NaCl, 10% glycerol using a PD-10 column (Cytiva), followed by measurement of protein concentration using Bradford Assay and storage of aliquots at −80 °C until needed.

Size-exclusion chromatography:

Ni-affinity-purified MppC wild-type and MppC-W336A/F340A were analyzed using a calibrated Superdex 75 increase 10/300 GL size exclusion chromatography column (Cytiva). The column was equilibrated with 25 mL of equilibration buffer (40 mM sodium phosphate (pH 7.5), 100 mM NaCl) at 0.3 mL/min flow rate, followed by the injection of 300 µL protein. 25 mL of equilibration buffer was then allowed to flow through the column at a flow rate of 0.3 mL/min. Protein eluate was collected in 0.3 mL fractions for SDS-PAGE analysis, and raw data of protein elution profiles were exported and plotted using Prism (GraphPad). The Superdex 75 increase 10/300 GL size exclusion chromatography column used in this study was calibrated using Gel Filtration Calibration Kit low molecular weights (LMW) (Cytiva).

Enzymatic assays

Prenylation of MppM-modified MppE substrate:

Typical 100 μL reactions contained 20 mM HEPES-Na (pH 7.5), 5 mM $MgCl_2$, 5 mM DTT, 100 μM MppM-modified MppE substrate peptide, 2 μM MppM, and 0.5 mM ATP. Reactions were incubated at 30 °C for 1 h, followed by addition of 5 mM $MgCl_2$, 10 μM MppC, and 200 μM prenyl donor (dimethylallyl diphosphate (DMAPP), geranyl diphosphate (GPP), farnesyl diphosphate (FPP), or geranylgeranyl diphosphate (GGPP)). The reactions were further incubated at 30 °C for 2 h. After 2 h, 0.057 M HCl was added, followed by brief mixing and reactions were incubated on ice for 5 min. 0.057 M NaOH was then added to neutralize the reaction mixtures and reaction tubes centrifuged at 16,800×g for 5 min to remove precipitates. 10 μM LahT150 peptidase was then added to clear reaction mixtures and incubated at 30 °C for 2 h to excise the $MppE^{leader}$.[2] To quench the peptidase reaction, equal volume of 98% (v/v) MeOH + 2% (v/v) formic acid was added to the reactions, followed by centrifugation at 16,800×g for 30 min. The resulting clarified mixtures were analyzed for macrocyclic $MppE^{core}$ peptide (substrate) and prenylated macrocyclic $MppE^{core}$ peptide (product) present using liquid chromatography/mass spectrometry (LC/MS). Reactions with macrocyclic MppE-W80F and MppE-W82F were conducted under similar conditions, except that the reactions were conducted with only GPP. Competition experiment for prenyl donors was also performed under similar conditions, except that each replicate reaction mixture contained all four prenyl donors (200 μM each of DMAPP, GPP, FPP, and GGPP). All reactions in this study were performed in triplicate.

Prenylation of linear MppE substrate with prenyl groups:

100 μL reactions containing 20 mM HEPES-Na (pH 7.5), 5mM $MgCl_2$, 5 mM DTT, 100 μM linear MppE substrate, 10 μM MppC, and 200 μM prenyl donor (DMAPP, GPP, FPP, or GGPP) were incubated at 30 °C for 2 h, followed by addition of 0.057 M HCl, brief mixing, and incubation on ice for 5 min. Reaction mixtures were neutralized by 0.057 M NaOH and centrifuged at 16,800×g for 5 min to remove enzyme precipitates. $MppE^{leader}$ was cleaved by the addition of 10 μM LahT150 peptidase followed by incubation at 30 °C for 2 h. Proteolysis by LahT150 was quenched by adding 98% (v/v) MeOH + 2% (v/v) formic acid to the reaction mixtures, followed by centrifugation at 16,800×g for 30 min. Reaction mixtures were analyzed using LC/MS.

Geranylation of macrocyclic $MppE^{core}$ peptide without the $MppE^{leader}$:

100 μL reactions containing 20 mM HEPES-Na (pH 7.5), 5mM $MgCl_2$, 5 mM DTT, 100 μM MppM-modified MppE substrate, 2 μM MppM, and 0.5 mM ATP were incubated at 30 °C for 1 h, followed by the addition of 50 μM LahT150 and incubation at 30 °C for 2 h to release the macrocyclic $MppE^{core}$

peptide from MppE^{leader}. The reaction mixtures were then treated with 0.057 M HCl, followed by the addition of 0.057 M NaOH and centrifugation at 16,800×g for 5 min. 5 mM $MgCl_2$, 10 μM MppC, and 200 μM GPP were added to the clear reaction mixtures, which were then incubated at 30 °C for 2 h. Reactions were quenched by 98% (v/v) MeOH + 2% (v/v) formic acid and analyzed by LC/MS.

Time-course geranylation of MppM-modified MBP-MppE and MBP-(Δ1–55)MppE substrates:

220 μL reactions containing 20 mM HEPES-Na (pH 7.5), 5mM $MgCl_2$, 5 mM DTT, 100 μM MppM-modified MBP-MppE or MBP-(Δ1–55)MppE, 30 μM MppM, and 0.5 mM ATP were incubated at 30 °C for 4 h, followed by addition of 5 mM $MgCl_2$, 10 μM MppC, and 200 μM GPP and incubation at 30 °C. 50 μL aliquots were taken from the reaction mixtures at 30 min intervals over 2 h, quenching the reaction with 0.057 M HCl. 0.057 M NaOH was then added to each aliquot and centrifuged at 16,800×g for 5 min. MBP-MppE^{leader} and the MBP-(Δ1–55)MppE^{leader} were cleaved with 10 μM LahT150 peptidase, followed by quenching with 98% (v/v) MeOH + 2% (v/v) formic acid and analysis by LC/MS.

Geranylation of MppM-modified MppE and MppE-L(X)$_4$L mutants:

100 μL reactions containing 20 mM HEPES-Na (pH 7.5), 5mM $MgCl_2$, 5 mM DTT, 100 μM MppM-modified MppE or MppE-L(X)$_4$L mutants, 20 μM MppM, and 0.5 mM ATP were incubated at 30 °C for 4 h, followed by addition of 5 mM $MgCl_2$, 10 μM MppC, and 200 μM GPP and incubation at 30 °C for 2 h. After 2 h, 0.057 M HCl was added to the reaction mixtures, gently mixed, and neutralized with 0.057 M NaOH. 2 μg/mL Glu-C protease (Promega) was added to clear reaction mixtures and incubated at 30 °C for 4 h to cleave MppE^{leader} and mutants thereof, followed by quenching with 98% (v/v) MeOH + 2% (v/v) formic acid for LC/MS analysis.

Geranylation of MppM-modified MppE-W80F/W82H and MppE-W80F/W82Y substrates:

100 μL reactions containing 20 mM HEPES-Na (pH 7.5), 5mM $MgCl_2$, 5 mM DTT, 50 μM MppM-modified MppE-W80F/W82H or MppE-W80F/W82Y, 15 μM MppM, and 0.5 mM ATP were incubated at 30 °C for 4 h, followed by addition of 5 mM $MgCl_2$, 5 μM MppC, and 100 μM GPP and incubation at 30 °C for 18 h. 0.057 M HCl was then added to the reaction mixtures, gently mixed, and neutralized with 0.057 M NaOH. MppE^{leader} was excised by the addition of 5 μM LahT150 peptidase and incubation at 30 °C for 2 h. Proteolysis reaction was quenched with 98% (v/v) MeOH + 2% (v/v) formic acid, followed by centrifugation and LC/MS analysis.

Time-course geranylation of MppM-modified MppE substrate by MppC and mutants thereof:

220 µL reactions containing 20 mM HEPES-Na (pH 7.5), 5mM $MgCl_2$, 5 mM DTT, 100 µM MppM-modified MppE substrate (~98% macrocycle from co-expression), 2 µM MppM, and 0.5 mM ATP were incubated at 30 °C for 1 h, followed by addition of 5 mM $MgCl_2$, 10 µM MppC or mutants thereof, and 200 µM GPP and incubation at 30 °C for 2 h. 50 µL aliquots were taken at 30 min intervals over 2 h, quenched with 0.057 M HCl, and neutralized with an equal concentration of NaOH, followed by centrifugation. The resulting clear reaction mixtures were treated with 10 µM LahT150 at 30 °C for 2 h, followed by quenching and analysis by LC/MS.

LC/MS protocol

Following proteolytic removal of the leader peptides and subsequent addition of 98% (v/v) MeOH + 2% (v/v) formic acid, clear reaction mixtures were analyzed on an HPLC system using an Agilent Poroshell 120 EC-C_{18} 2.7 µm 4.6×100 mm column. Separation of molecules in the reaction mixtures was performed at 0.3 mL/min flow rate, using 2% solvent B (MeCN + 0.1% (v/v) formic acid) from 0–5 min, increasing solvent B to 100% from 5–18 min in a linear gradient, holding solvent B at 100% from 18–22 min, decreasing solvent B to 2% from 22–24 min in a linear gradient, and then using 2% solvent B from 24–30 min. Solvent A composition was H_2O + 0.1% (v/v) formic acid. Mass analysis of separated molecules was performed on an Agilent 6530 Quadrupole-Time-of-Flight mass spectrometer coupled to the HPLC system. Electrospray ionization (ESI) source was used to generate ions that were analyzed in the positive ionization mode, and data acquisition was performed for ions in the MS range of 400–3000 *m/z*. The MS range was changed to 300–3000 Da *m/z* for MppE-W80F/W82H^{core} and MppE-W80F/W82Y^{core} peptide data acquisition. MS range of 100–3000 Da *m/z* was used when L-Trp was utilized as a substrate. Agilent MassHunter software was used to analyze acquired MS data, and quantification of substrates and products was performed by calculating the areas under corresponding extracted ion chromatogram (EIC) peaks.

Preparative-scale purification of geranylated macrocyclic MppE-W80F^{Core} peptide for NMR

The Gly77 in MppE precursor peptide was mutated to a Lys residue for a preparative-scale trypsin digestion of modified MppE to obtain the geranylated macrocyclic product for NMR. 250 mg of purified MppM-modified MppE-G77K/W80F peptide was geranylated in batches of 1 mL reactions containing 20 mM HEPES-Na (pH 7.5), 20 mM $MgCl_2$, 5 mM DTT, 200 µM MppM-modified MppE-G77K/W80F, 20 µM MppC, 4 µM MppM, 400 µM GPP, and 1 mM ATP, incubated at 30 °C for 14 h. The reaction mixtures were treated with 0.07125 M HCl and incubated on ice for 30 min, followed by the addition of 0.07125 M

NaOH. The resulting mixture was centrifuged (29,416×g, 30 min, 4 °C, Avanti®J-E) to remove all enzyme precipitates. The clear reaction mixture containing geranylated MppM-modified MppE-G77K/W80F was then treated with trypsin protease in batches of 1 mL reactions containing 4 μM trypsin, 50 mM Tris-Cl (pH 8.0), and 5 mM DTT, incubated at 30 °C for 6 h. Reaction mixtures were centrifuged (29,416×g, 30 min, 18 °C, Avanti®J-E), followed by separation of peptide fragments using solid phase extraction (SPE). Strata™-X 33 μm polymeric reversed phase tube containing 200 mg sorbent (Phenomenex) was utilized for SPE method development on a small scale, monitoring fractions for geranylated macrocyclic MppE-W80F^{core} peptide by LC/MS. Strata™-X 33 μm polymeric reversed phase tube containing 2 g sorbent (Phenomenex) was then used for large-scale extraction of geranylated macrocyclic MppE-W80F^{core} peptide. Three 2 g resin tubes were each conditioned with 40 mL MeOH, equilibrated with 40 mL Milli-Q H_2O, followed by the loading of ~50 mL clear mixture of peptide fragments per tube. Each tube was reloaded with the flow-through multiple times under gravity flow. Tubes were then washed with MeOH/H_2O using 40 mL 10% (v/v) MeOH, 40 mL 20% (v/v) MeOH, 40 mL 40% (v/v) MeOH, and 40 mL 60% (v/v) MeOH, in that order, collecting fractions for LC/MS analysis. Elution of less-polar peptide fragments was then carried out by using 40 mL MeCN (2×) and 40 mL 98% (v/v) MeCN + 2% (v/v) formic acid (3×), monitoring fractions by LC/MS. Geranylated macrocyclic MppE-W80F^{core} peptide eluted in the first 40 mL 98% (v/v) MeCN + 2% (v/v) formic acid fraction. Acetonitrile was removed from this fraction by using a rotary evaporator followed by the removal of water using a freeze dryer (Labconco). Dried geranylated macrocyclic MppE-W80F^{core} peptide was dissolved in 600 μL DMSO-*d6* for NMR data acquisition.

One-dimensional (1D) and two-dimensional (2D) NMR experiments were carried out using a Bruker Avance III HD spectrometer operating at 700 MHz equipped with a 5 mm TCI cryoprobe. Processed NMR data were analyzed using MestReNova v14.2.0-26256, referencing chemical shifts for the ^{1}H and ^{13}C NMR spectra against the chemical shifts of DMSO-*d6* at δ_H 2.50 ppm and δ_C 39.52 ppm.

Sequence similarity network generation and genome mining

A sequence similarity network (SSN) was created using MppC as the query sequence searched against the UniProt database with a default e-value of 5.0 and a maximum limit on the number of retrieved sequences set to 1,000 between 200 and 1,000 amino acid length using the online EFI-EST toolkit (https://efi.igb.illinois.edu/efi-est/).[3] The 258 sequences thusly retrieved (252 unique) were organized into 229 nodes using an empirically determined alignment sequence threshold of 75 and a sequence similarity cutoff of 95%. The resulting SSN is illustrated in Figure S67. Crucially, the SSN created using MppC as the query sequence and the abovementioned search and sequence organization criteria did not include sequences for leader-independent cyanobactin prenyltransferases such as PagF. In the same vein, a SSN

created using PagF as the query sequence did not include MppC as one of the nodes. This implies that the leader-dependent (such as MppC) and leader-independent (such as PagF) prenyltransferases are divergent in their sequences.

The most dominant bacterial phyla represented in the SSN resulting from using MppC as the query sequence were Cyanobacteriota (67 nodes, 29% of all nodes in the SSN), Myxococcota (53, 23%), and Pseudomonadota (27, 12%). Thus, these three bacterial phyla accounted for 64% of all nodes in the SSN. The node corresponding to MppC clustered in a subnetwork comprising majorly of Cyanobacteriota-derived nodes. As judged by gene neighborhood networks, 76 MppC-like sequences in the SSN colocalized with LanM-like proteins (LanMs were denoted by the Pfam annotation PF05147-PF13575). Furthermore, 87 MppC-like sequences colocalized with Nif11-domain containing open reading frames (ORFs) as denoted by the Pfam PF07862. Akin Nif11-like leader sequences, the nitrile hydratase-like leader peptides (NHLPs) are long and structured; 9 MppC-like sequences colocalized with NHLPs as denoted by the Pfam PF02979.

SUPPLEMENTARY TABLES

Table S1: Sequences of peptides used in this study

Peptide/Enzyme	Amino Acid Sequence
MBP	MGSSHHHHHH[‡]SSGLVPRGS[†]HMKIEEGKLVIWINGDKGYNGLAEVGK<br>KFEKDTGIKVTVEHPDKLEEKFPQVAATGDGPDIIFWAHDRFGGYAQSG<br>LLAEITPDKAFQDKLYPFTWDAVRYNGKLIAYPIAVEALSLIYNKDLLPN<br>PPKTWEEIPALDKELKAKGKSALMFNLQEPYFTWPLIAADGGYAFKYE<br>NGKYDIKDVGVDNAGAKAGLTFLVDLIKNKHMNADTDYSIAEAAFNK<br>GETAMTINGPWAWSNIDTSKVNYGVTVLPTFKGQPSKPFVGVLSAGIN<br>AASPNKELAKEFLENYLLTDEGLEAVNKDKPLGAVALKSYEEELAKDP<br>RIAATMENAQKGEIMPNIPQMSAFWYAVRTAVINAASGRQTVDEALKD<br>AQTNSSSHHHHHH[‡]ANSVPLVPRGS[†]ENLYFQS[§]GS
MppE	MSSEQIEQFVEEIQRDPALKEQLQLQGSIDETIDKVIEIAKEKGYDFTATE<br>LKEYMENPSDEEEELSDSELEAVAGG**ACWRWSG**
MBP-MppE	(MBP)-MSSEQIEQFVEEIQRDPALKEQLQLQGSIDETIDKVIEIAKEKGY<br>DFTATELKEYMENPSDEEEELSDSELEAVAGG**ACWRWSG**
MBP-(Δ1–55)MppE	(MBP)-MENPSDEEEELSDSELEAVAGG**ACWRWSG**
MppE-A(X)$_4$A	MSSEQIEQFVEEIQRDPALKEQLQLQGSIDETIDKVIEIAKEKGYDFTATE<br>LKEYMENPSDEEEEASDSEAEAVAGG**ACWRWSG**
MppE-F(X)$_4$F	MSSEQIEQFVEEIQRDPALKEQLQLQGSIDETIDKVIEIAKEKGYDFTATE<br>LKEYMENPSDEEEEFSDSEFEAVAGG**ACWRWSG**
MppE-V(X)$_4$V	MSSEQIEQFVEEIQRDPALKEQLQLQGSIDETIDKVIEIAKEKGYDFTATE<br>LKEYMENPSDEEEEVSDSEVEAVAGG**ACWRWSG**
MppE-G77K	MSSEQIEQFVEEIQRDPALKEQLQLQGSIDETIDKVIEIAKEKGYDFTATE<br>LKEYMENPSDEEEELSDSELEAVAGK**ACWRWSG**
MppE-W80F	MSSEQIEQFVEEIQRDPALKEQLQLQGSIDETIDKVIEIAKEKGYDFTATE<br>LKEYMENPSDEEEELSDSELEAVAGG**ACFRWSG**
MppE-W82F	MSSEQIEQFVEEIQRDPALKEQLQLQGSIDETIDKVIEIAKEKGYDFTATE<br>LKEYMENPSDEEEELSDSELEAVAGG**ACWRFSG**
MppE- G77K/W80F	MSSEQIEQFVEEIQRDPALKEQLQLQGSIDETIDKVIEIAKEKGYDFTATE<br>LKEYMENPSDEEEELSDSELEAVAGK**ACFRWSG**
MppE-W80F/W82H	MSSEQIEQFVEEIQRDPALKEQLQLQGSIDETIDKVIEIAKEKGYDFTATE<br>LKEYMENPSDEEEELSDSELEAVAGG**ACFRHSG**
MppE-W80F/W82Y	MSSEQIEQFVEEIQRDPALKEQLQLQGSIDETIDKVIEIAKEKGYDFTATE<br>LKEYMENPSDEEEELSDSELEAVAGG**ACFRYSG**

[‡]His$_6$ tag, [†]thrombin cleavage site, [§]TEV protease cleavage site

core peptides in boldface

Table S2: NMR chemical shifts of geranylated macrocyclic MppE-W80F^{core} peptide in DMSO-*d6*.

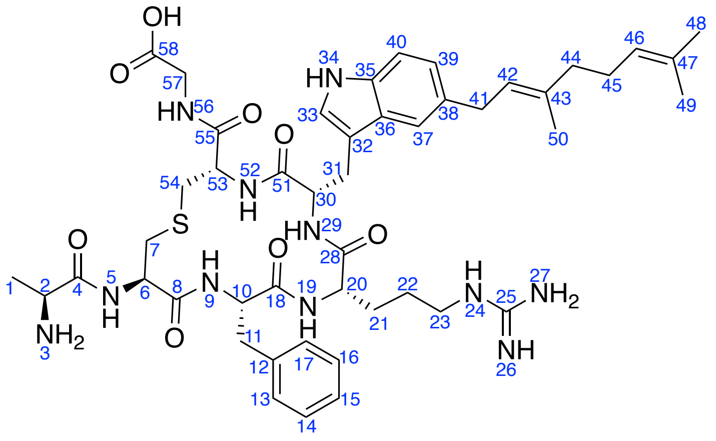

Amino acid	position	δ_H, multiplicity	δ_C	COSY	HMBC	TOCSY	ROESY	NOESY
Ala	1	1.16, d	17.96, CH_3	2	2, 4	2	2	2
	2	3.69, br. m	48.22, CH	1	1, 4	1	5	5
	3		N/A					
	4		170.10, C					
Cys	5	8.46, d	N/A	6	4	6, 7a, 7b	2	2
	6	4.45, br. m	51.84, CH	5, 7a	7, 8	7a, 7b		
	7a	2.79, br. m	34.31, CH_2	6, 7b	6, 8, 54	5, 6		
	7b	2.67, br.	34.31, CH_2	7a		5, 6		
	8		169.07, C					
Phe	9	9.13, br.	N/A	10		10, 11		10, 11
	10	4.44, br. m	55.45, CH	9, 11	11, 12, 18	9, 11	17, 19	9, 11
	11	2.89, br. m	37.88, CH_2	10	10, 12, 13, 18	9, 10	13	9, 10, 13
	12		137.40, C					
	13	7.21, br. m	129.06, CH		11		11	11
	14	7.21, br. m	128.18, CH	15	12			
	15	7.16, t	126.28, CH	14	13			
	16	7.21, br. m	128.18, CH	15	12			
	17	7.21, br. m	129.06, CH		11		10	19
	18		171.48, C					
Arg	19	9.17, br.	N/A	20		20	10	10, 11, 17, 20
	20	3.85, br. s	54.68, CH	19		19		
	21							
	22							
	23							
	24		N/A					
	25		157.21, C					

	26		N/A					
	27		N/A					
	28							
Trp	29	8.21, br.	N/A	30		30, 31b		30, 31b
	30	4.38, br.	53.99, CH	29, 31b		29, 31a, 31b		31b
	31a	3.30, d		31b	32	30		
	31b	3.16, br.	57.55, CH_2	30, 31a	32	29, 30,		30
	32		110.12, C					
	33	7.07, s	123.88, CH		32, 35, 36		34	34
	34	10.73, s	N/A		32, 33, 35, 36		33, 40	33, 40
	35		134.79, C					
	36		127.35, C					
	37	7.27, s	117.05, CH		32, 35, 39, 41	41	41	41
	38		130.75, C					
	39	6.84, d	121.64, CH	40	35, 37, 41		41, 42	41
	40	7.21, br. m	111.27, CH	39	36, 38		34	34
	51							
geranyl	41	3.34, d	33.97, CH_2	42	37, 38, 42, 43	37, 42	37, 39, 42	37, 39
	42	5.32, t	124.66, CH	41	38, 41, 44, 50	41, 44, 50	39, 41, 44	
	43		134.20, C					
	44	1.99, t	39.06, CH_2	45	42, 43, 45, 50	42, 46	42	
	45	2.05, q	26.23, CH_2	44, 46	43, 44, 46, 47			
	46	5.07, t	124.19, CH	45	44, 48, 49	44	48	
	47		130.99, C					
	48	1.62, s	25.53, CH_3		46, 47, 49		46	
	49	1.55, s	17.56, CH_3		46, 47, 48	46		
	50	1.70, s	15.94, CH_3		42, 43, 44	41, 42		
Ser	52	8.21, br.	N/A	53		53, 54		53, 54
	53	4.38, br.	53.99, CH	52	54	52, 54	54	
	54	2.89, br. m	33.43, CH_2			52, 53	53	
	55							
Gly	56	7.76, br. s	N/A	57		57	53, 57	53, 57
	57	3.69, br. m	43.38, CH_2	56		56	56	56
	58							

SUPPLEMENTARY FIGURES

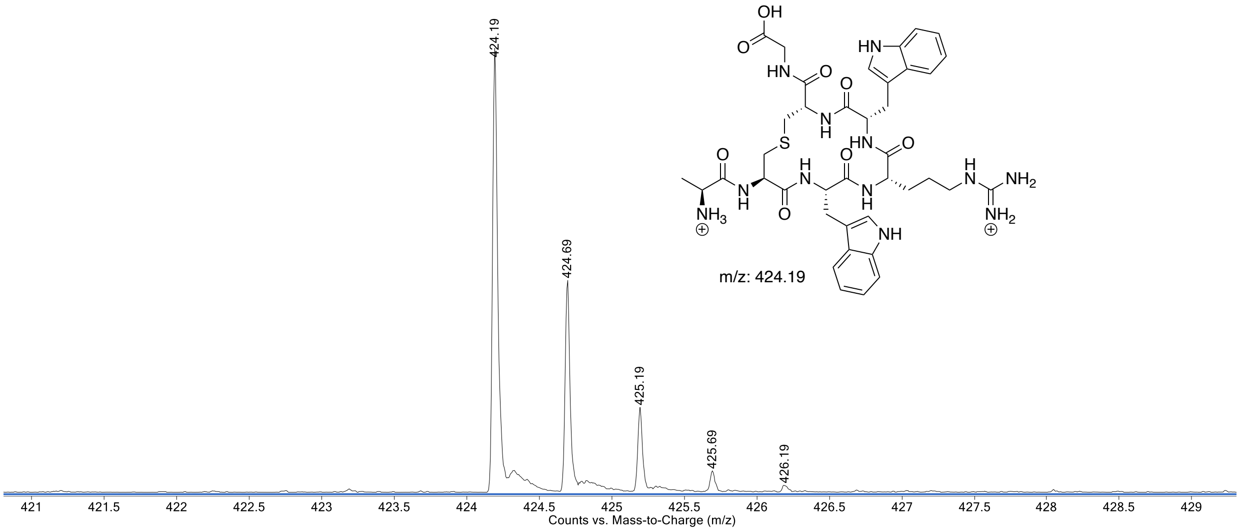

Figure S1: MS[1] spectrum demonstrating the isotopic distribution of the $[M+2H]^{2+}$ ions corresponding to macrocyclic MppE^{core} peptide (theoretical *m/z*: 424.19) following excision of MppE^{leader} peptide by LahT150.

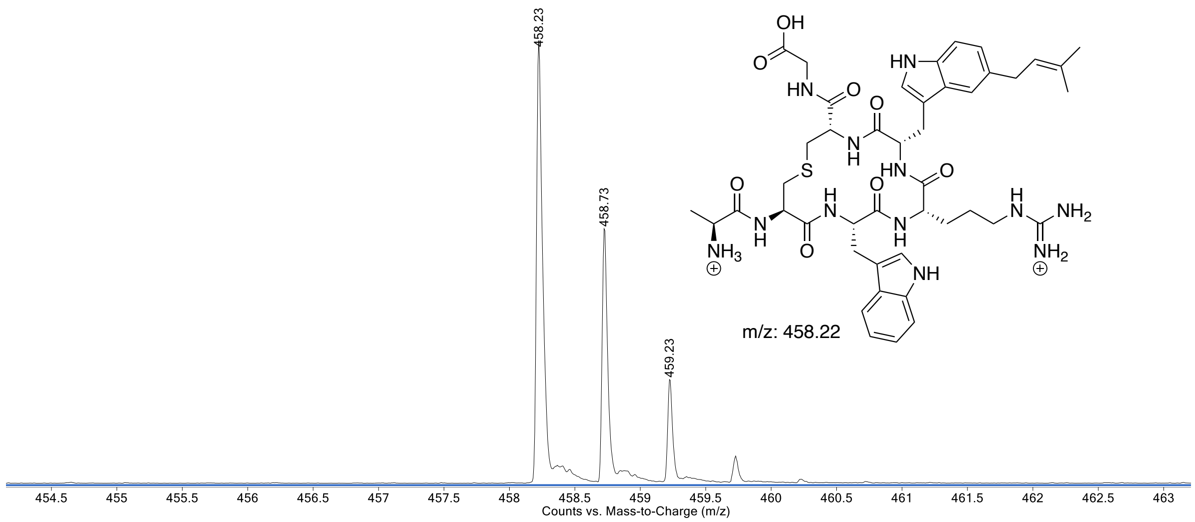

Figure S2: MS[1] spectrum demonstrating the isotopic distribution of the $[M+2H]^{2+}$ ions corresponding to C_5-prenylated macrocyclic MppE^{core} peptide (theoretical *m/z*: 458.22) when MppM-modified MppE substrate peptide was incubated with dimethylallyl diphosphate (DMAPP) and purified MppC. MppE^{leader} peptide was excised by LahT150.

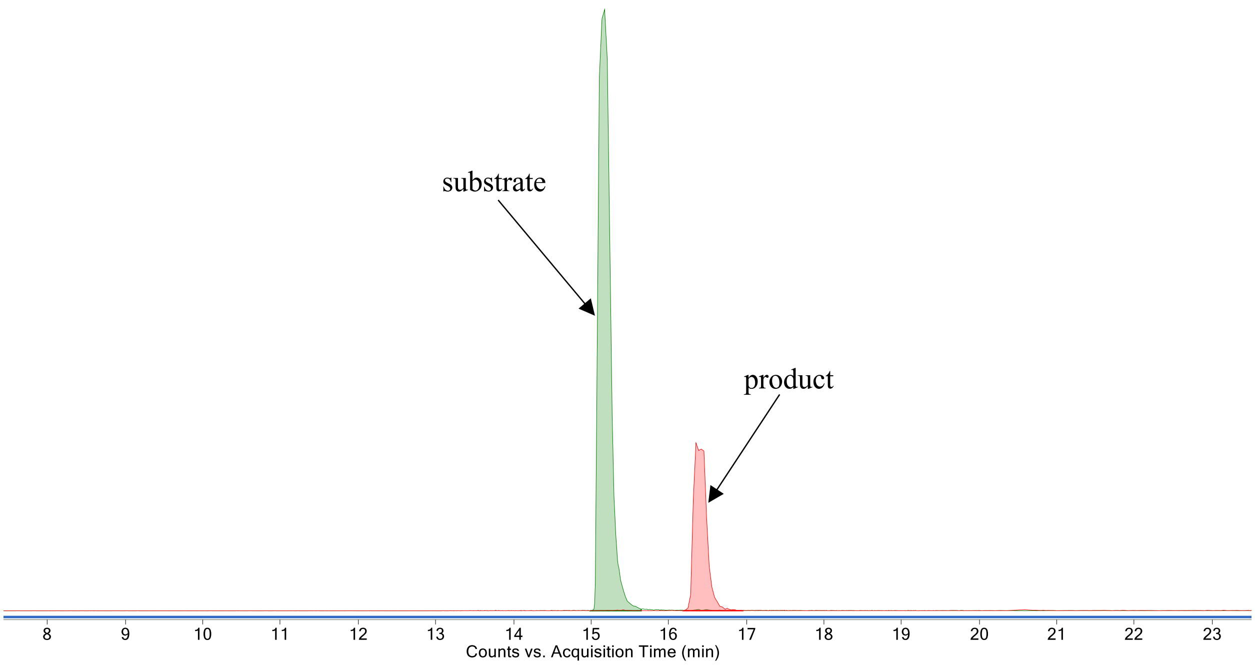

Figure S3: Extracted ion chromatograms (EICs) for the most abundant $[M+2H]^{2+}$ ions corresponding to macrocyclic MppE^{core} peptide (in green) and C_5-prenylated macrocyclic MppE^{core} peptide (in red) when MppM-modified MppE substrate peptide was incubated with DMAPP and purified MppC.

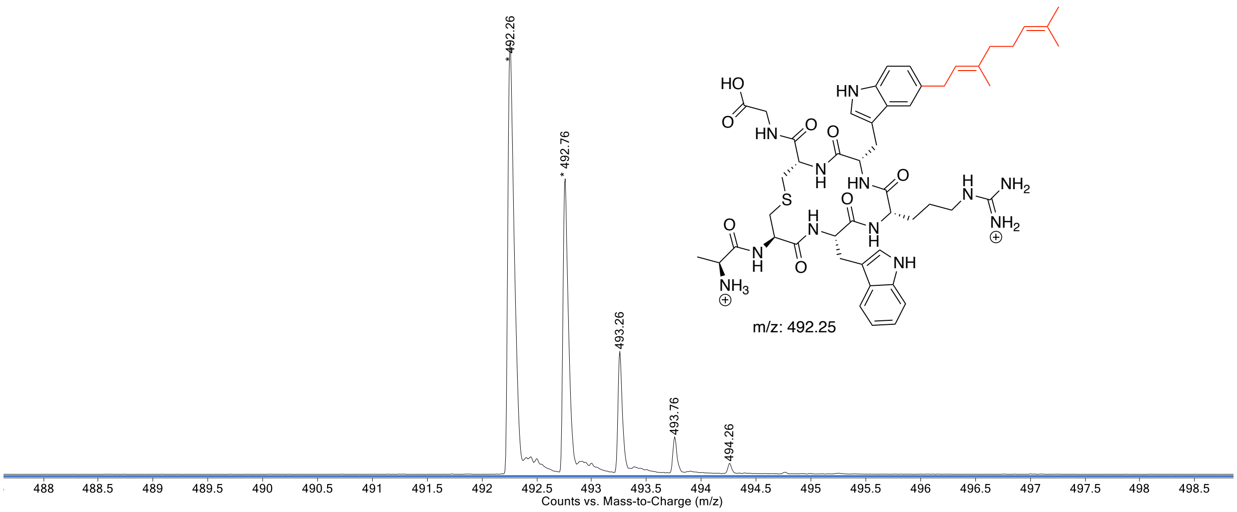

Figure S4: MS^1 spectrum demonstrating the isotopic distribution of the $[M+2H]^{2+}$ ions corresponding to C_{10}-prenylated macrocyclic $MppE^{core}$ peptide (theoretical *m/z*: 492.25) when MppM-modified MppE substrate peptide was incubated with geranyl diphosphate (GPP) and purified MppC. $MppE^{leader}$ peptide was excised by LahT150.

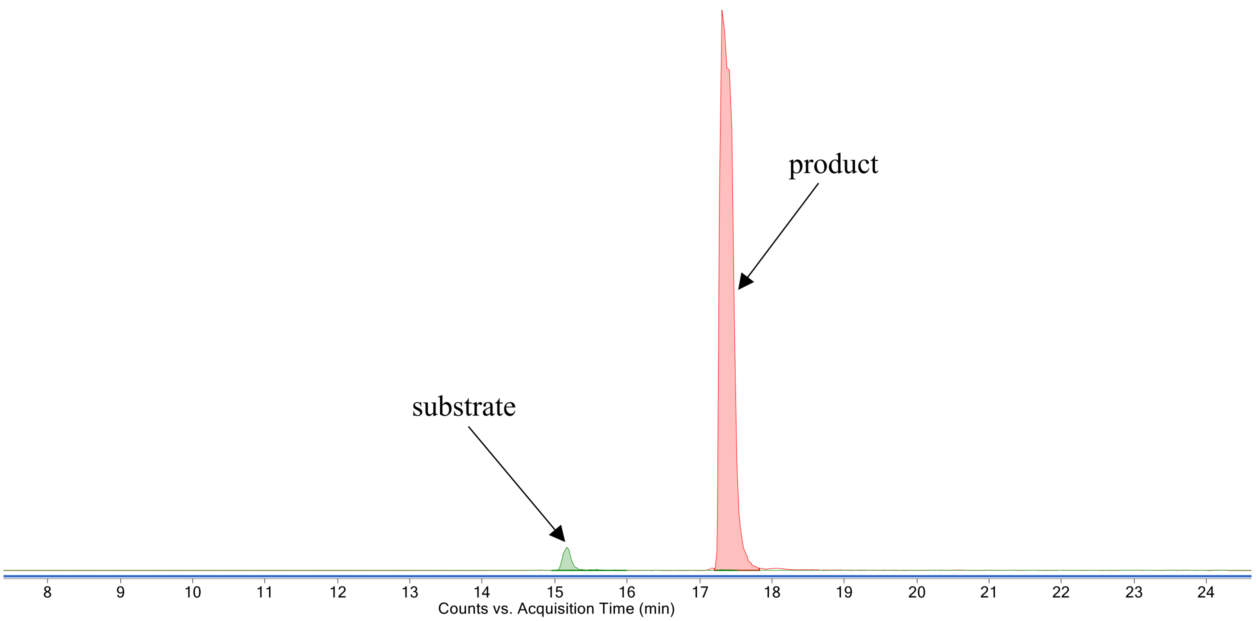

Figure S5: EICs for the most abundant $[M+2H]^{2+}$ ions corresponding to macrocyclic MppE^{core} peptide (in green) and C_{10}-prenylated macrocyclic MppE^{core} peptide (in red) when MppM-modified MppE substrate peptide was incubated with GPP and purified MppC.

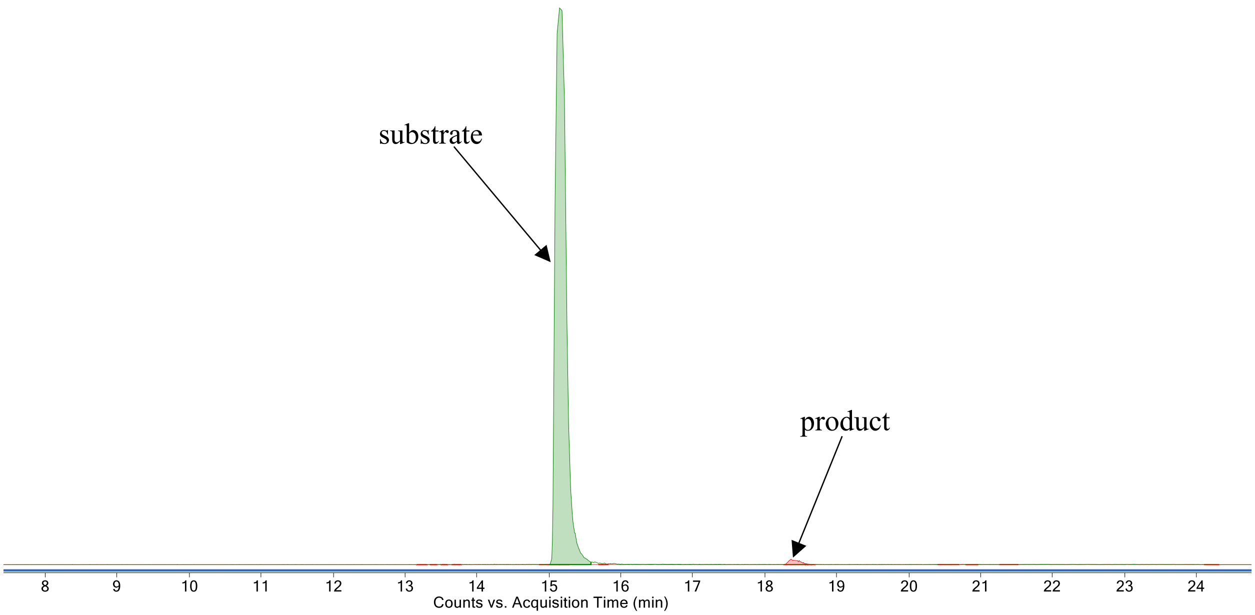

Figure S6: EICs for the most abundant $[M+2H]^{2+}$ ions corresponding to macrocyclic $MppE^{core}$ peptide (in green) and C_{15}-prenylated macrocyclic $MppE^{core}$ peptide (in red) when MppM-modified MppE substrate peptide was incubated with farnesyl diphosphate (FPP) and purified MppC. $MppE^{leader}$ peptide was excised by LahT150. Note that the MS^1 spectra for the product species denoted in this figure are not being shown as they do not conform to the theoretical isotopic distribution of the product species, indicating that no product was detected to be produced in this assay.

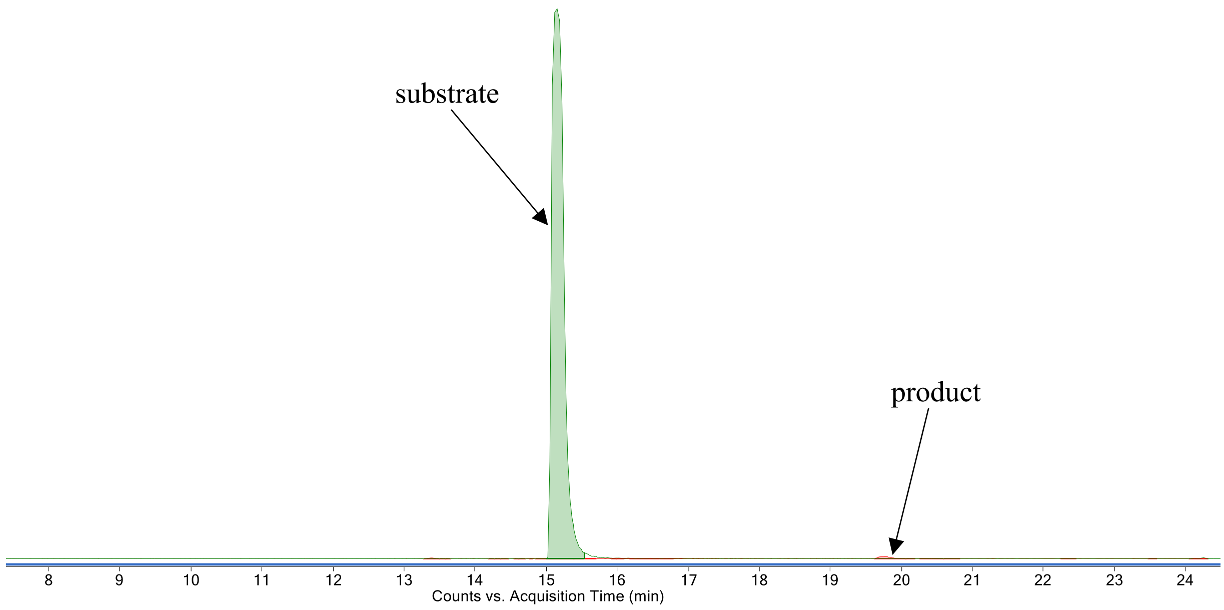

Figure S7: EICs for the most abundant $[M+2H]^{2+}$ ions corresponding to macrocyclic $MppE^{core}$ peptide (in green) and C_{20}-prenylated macrocyclic $MppE^{core}$ peptide (in red) when MppM-modified MppE substrate peptide was incubated with geranylgeranyl diphosphate (GGPP) and purified MppC. $MppE^{leader}$ peptide was excised by LahT150. Note that the MS^1 spectra for the product species denoted in this figure are not being shown as they do not conform to the theoretical isotopic distribution of the product species, indicating that no product was detected to be produced in this assay.

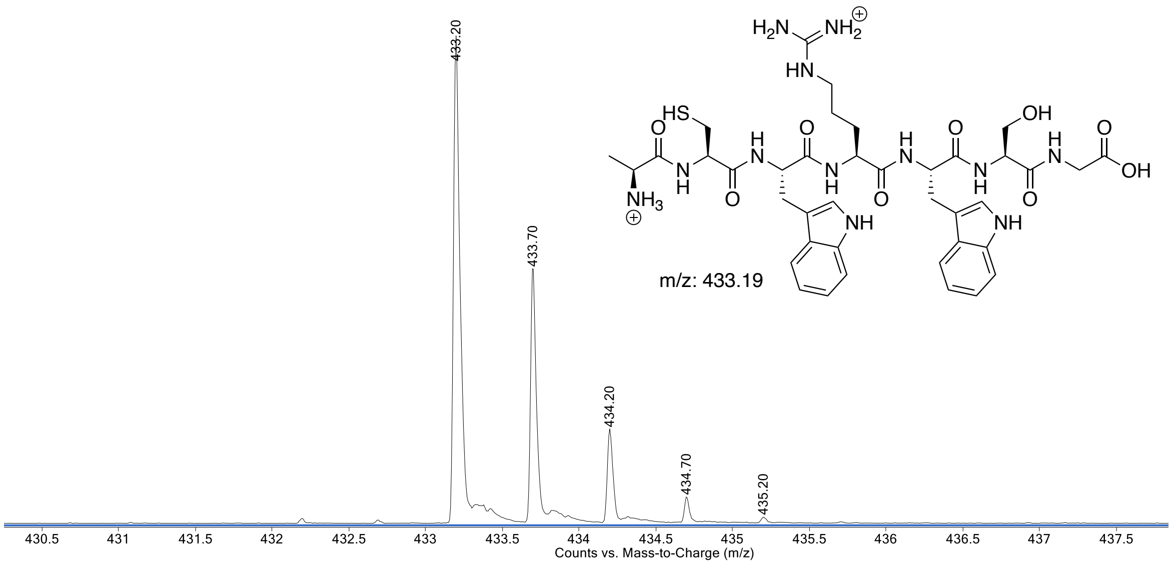

Figure S8: MS^1 spectrum demonstrating the isotopic distribution of the $[M+2H]^{2+}$ ions corresponding to linear MppE^{core} peptide (theoretical *m/z*: 433.19) following excision of MppE^{leader} peptide by LahT150.

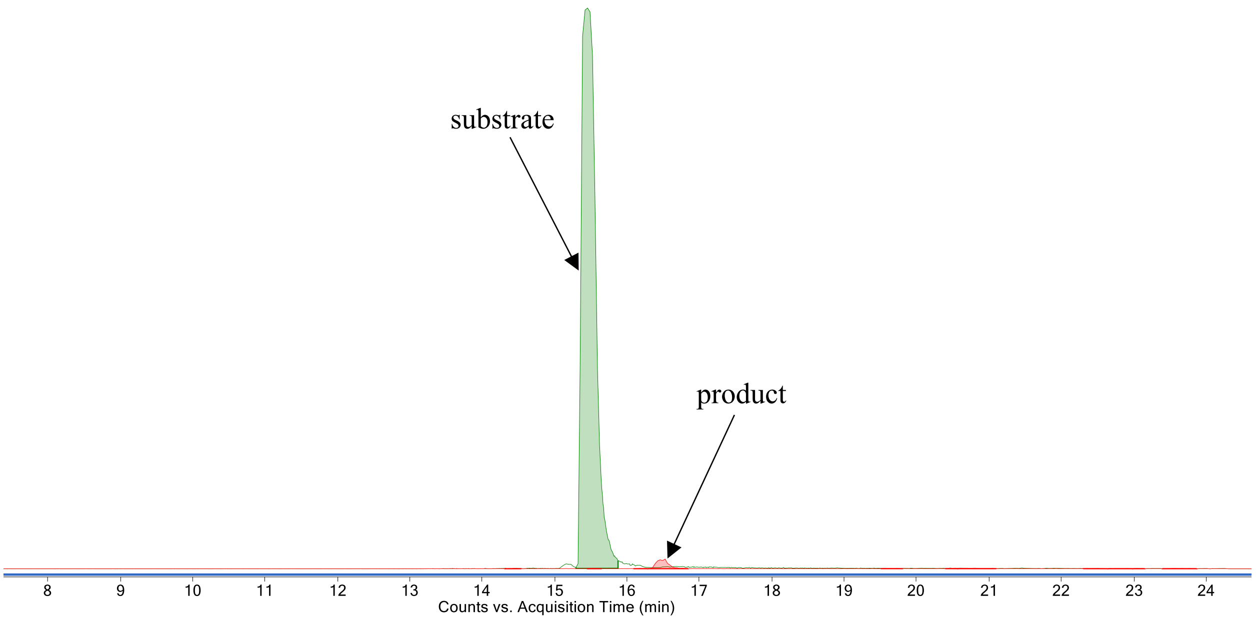

Figure S9: EICs for the most abundant $[M+2H]^{2+}$ ions corresponding to linear $MppE^{core}$ peptide (in green) and C_5-prenylated linear $MppE^{core}$ peptide (in red) when linear MppE substrate peptide was incubated with DMAPP and purified MppC. $MppE^{leader}$ peptide was excised by LahT150. Note that the MS^1 spectra for the product species denoted in this figure are not being shown as they do not conform to the theoretical isotopic distribution of the product species, indicating that no product was detected to be produced in this assay.

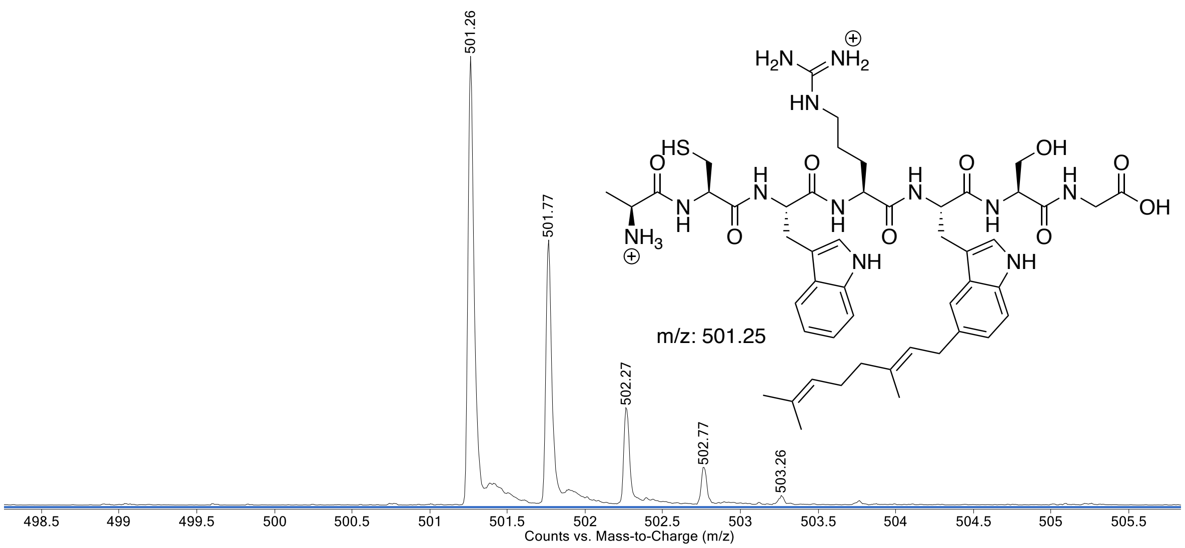

Figure S10: MS[1] spectrum demonstrating the isotopic distribution of the $[M+2H]^{2+}$ ions corresponding to C_{10}-prenylated linear MppE^{core} peptide (theoretical *m/z*: 501.25) following excision of MppE^{leader} peptide by LahT150.

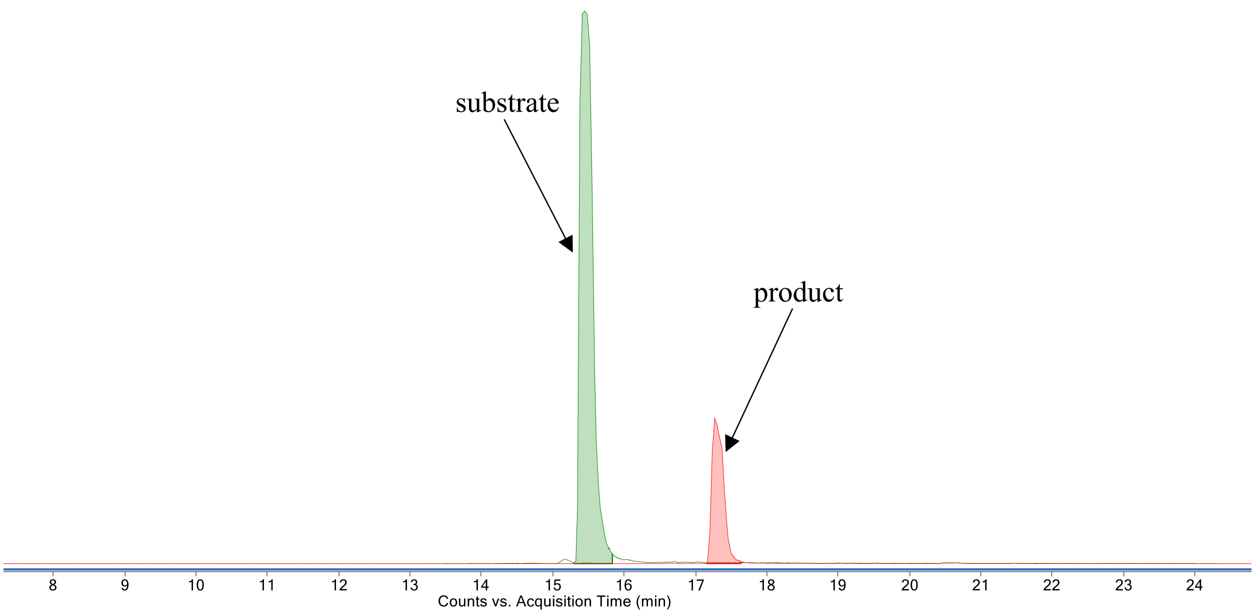

Figure S11: EICs for the most abundant $[M+2H]^{2+}$ ions corresponding to linear MppE^{core} peptide (in green) and C_{10}-prenylated linear MppE^{core} peptide (in red) when linear MppE substrate peptide was incubated with GPP and purified MppC.

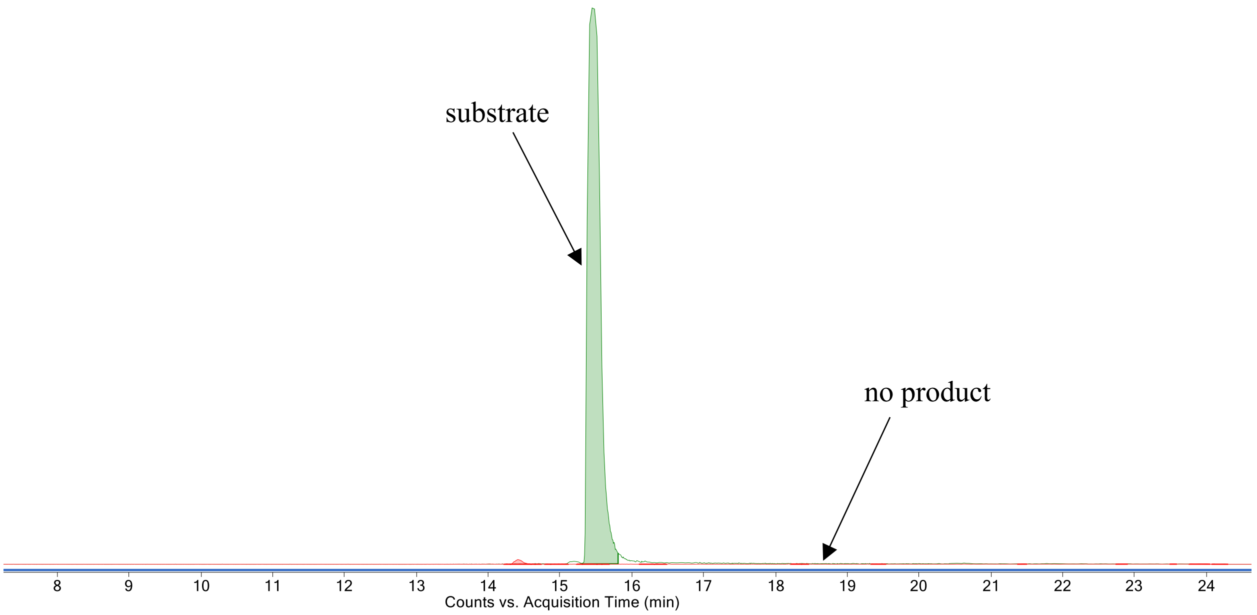

Figure S12: EICs for the most abundant $[M+2H]^{2+}$ ions corresponding to linear MppE^{core} peptide (in green) and C_{15}-prenylated linear MppE^{core} peptide (in red) when linear MppE substrate peptide was incubated with FPP and purified MppC. MppE^{leader} peptide was excised by LahT150. Note that the MS^1 spectra for the product species denoted in this figure are not being shown as they do not conform to the theoretical isotopic distribution of the product species, indicating that no product was detected to be produced in this assay.

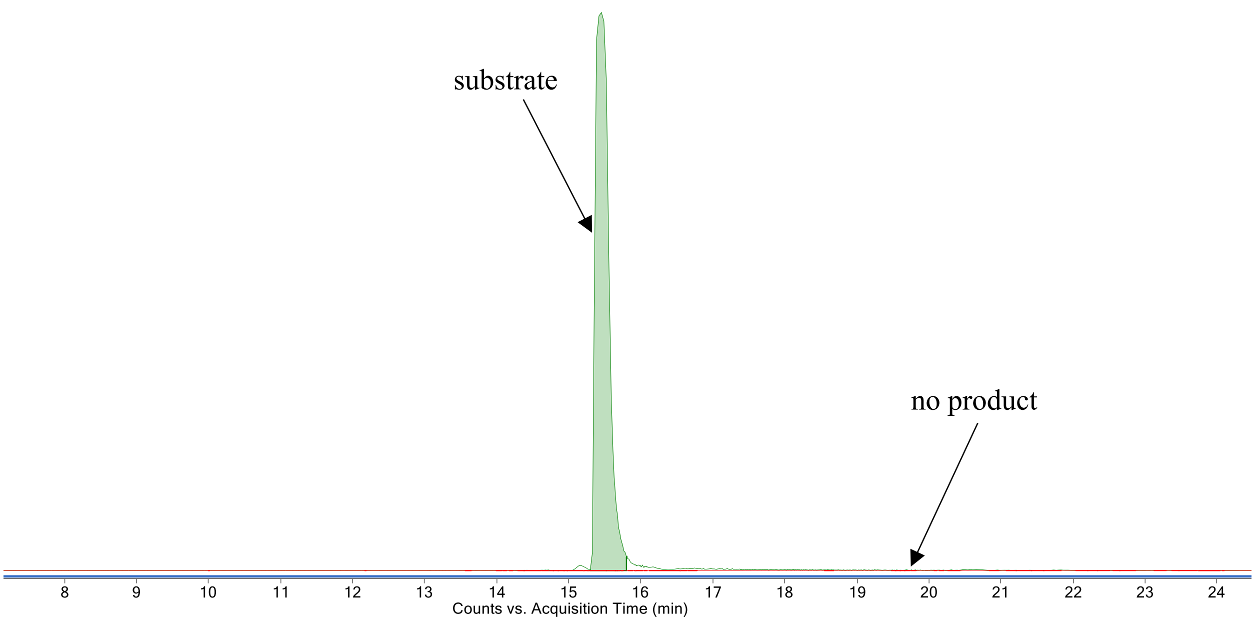

Figure S13: EICs for the most abundant $[M+2H]^{2+}$ ions corresponding to linear MppE^{core} peptide (in green) and C_{20}-prenylated linear MppE^{core} peptide (in red) when linear MppE substrate peptide was incubated with GGPP and purified MppC. MppE^{leader} peptide was excised by LahT150. Note that the MS^1 spectra for the product species denoted in this figure are not being shown as they do not conform to the theoretical isotopic distribution of the product species, indicating that no product was detected to be produced in this assay.

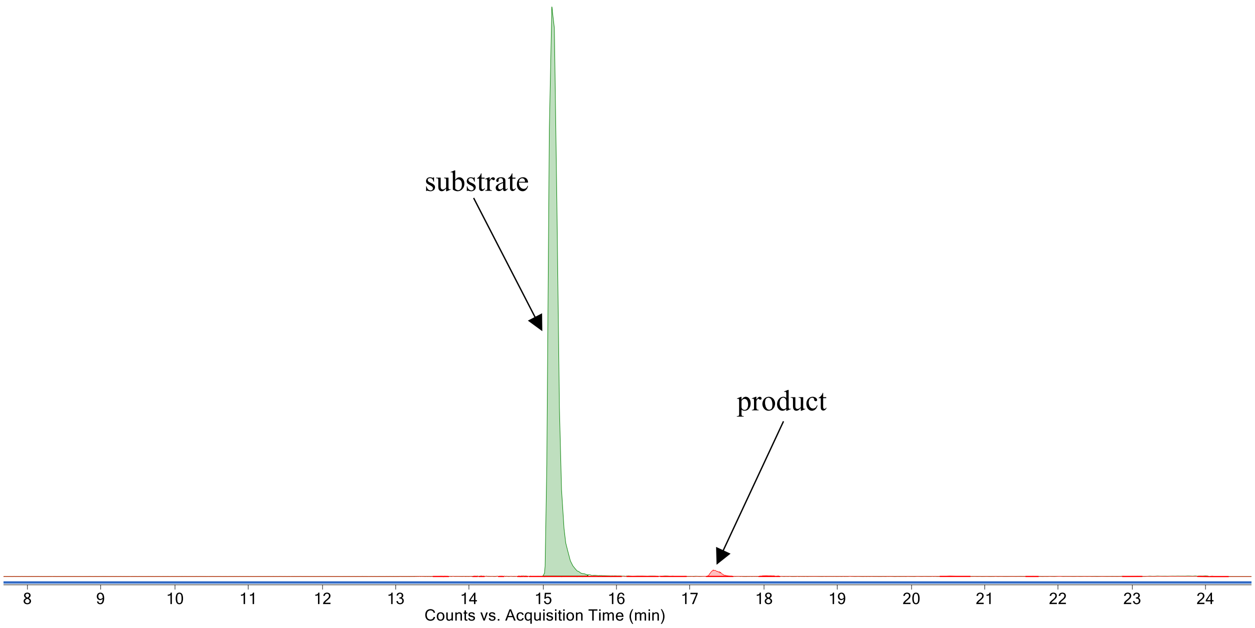

Figure S14: EICs for the most abundant $[M+2H]^{2+}$ ions corresponding to macrocyclic $MppE^{core}$ peptide (in green) and C_{10}-prenylated macrocyclic $MppE^{core}$ peptide (in red) when macrocyclic $MppE^{core}$ peptide (without $MppE^{leader}$) was incubated with GPP and purified MppC.

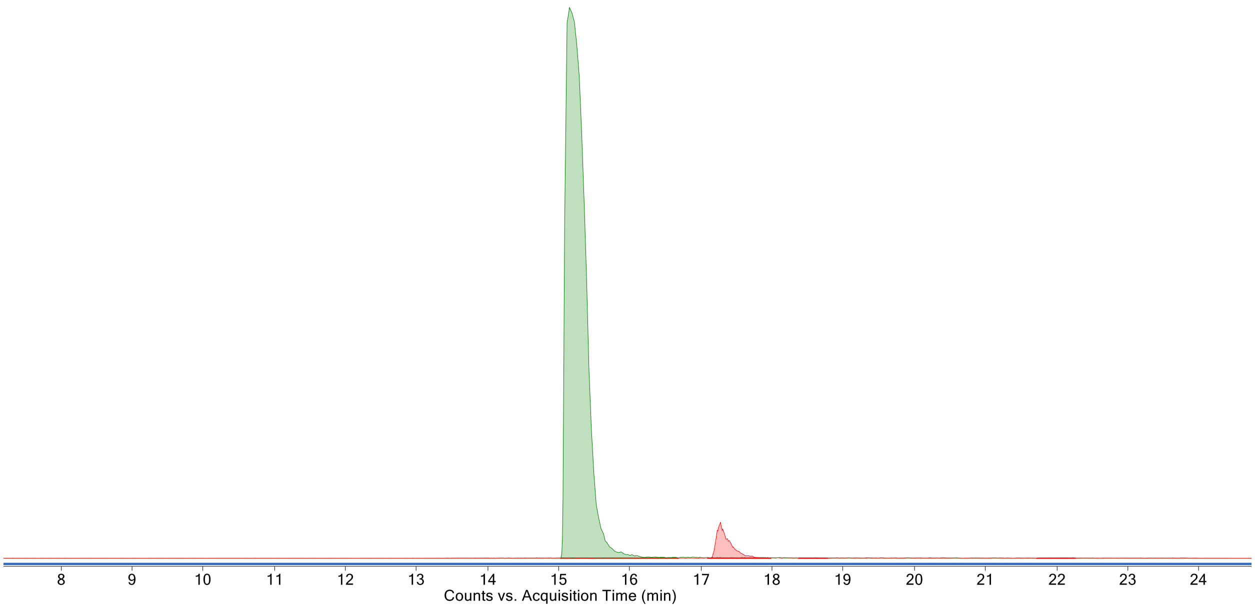

Figure S15: EICs for the most abundant $[M+2H]^{2+}$ ions corresponding to macrocyclic MppE^{core} peptide (in green) and C_{10}-prenylated macrocyclic MppE^{core} peptide (in red) when MppE^{leader} was provided *in trans* with the macrocyclic MppE^{core} peptide in an *in vitro* assay that involved substrate incubation with GPP and purified MppC. A substrate sample that possessed an equimolar ratio of the *in trans*-added MppE^{leader} with the macrocyclic MppE^{core} was generated by extensive digestion of the MppM-modified full length MppE substrate peptide with the LahT150 peptidase to release the MppE^{leader}.

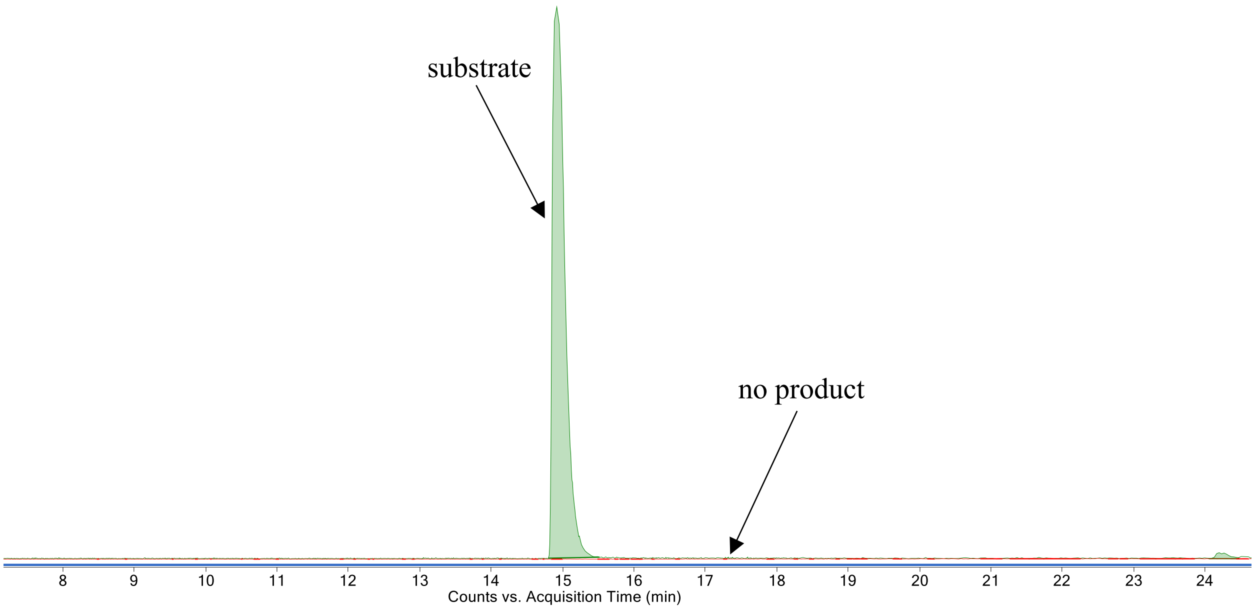

Figure S16: EICs for the most abundant $[M+H]^+$ ions corresponding to L-tryptophan substrate (in green) and C_{10}-prenylated L-tryptophan product (in red) when L-tryptophan was incubated with GPP and purified MppC for 2 h.

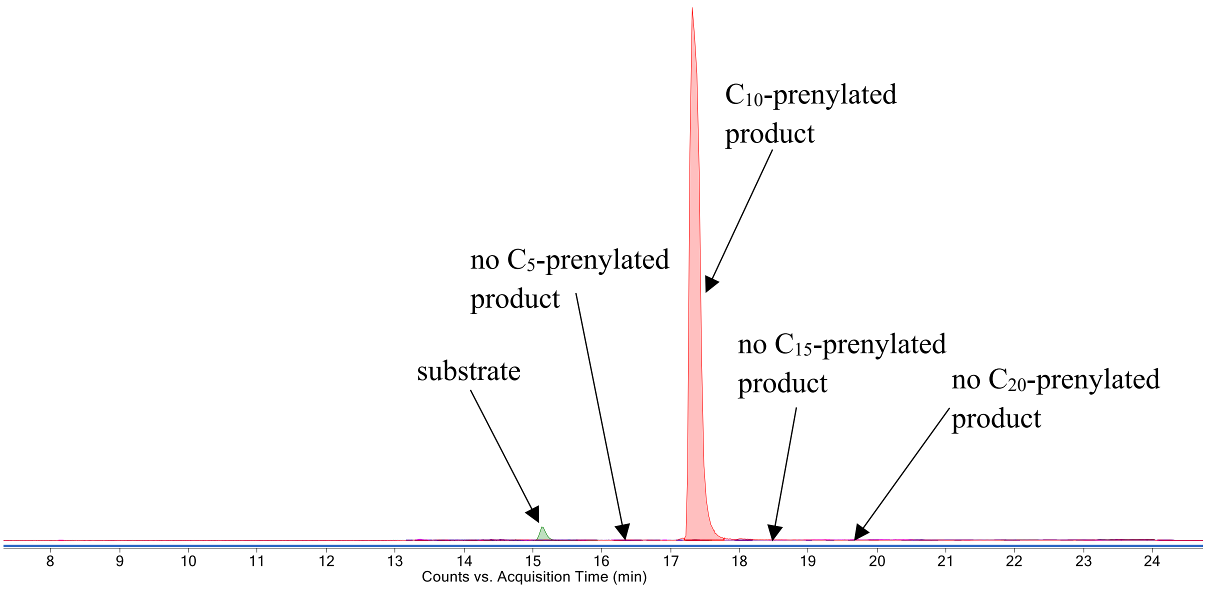

Figure S17: EICs for the most abundant $[M+2H]^{2+}$ ions corresponding to macrocyclic $MppE^{core}$ peptide (in green) and C_{10}-prenylated macrocyclic $MppE^{core}$ peptide (in red) when MppM-modified MppE substrate peptide was incubated with purified MppC and all four prenyl donors (DMAPP, GPP, FPP, and GGPP) in a competition experiment. $MppE^{leader}$ peptide was excised by LahT150. Note that the EICs for C_5-, C_{15}-, and C_{20}-prenylated macrocyclic $MppE^{core}$ peptides are not showing due to the absence of ions corresponding to these products, indicating that these products were not detected to be produced in this assay.

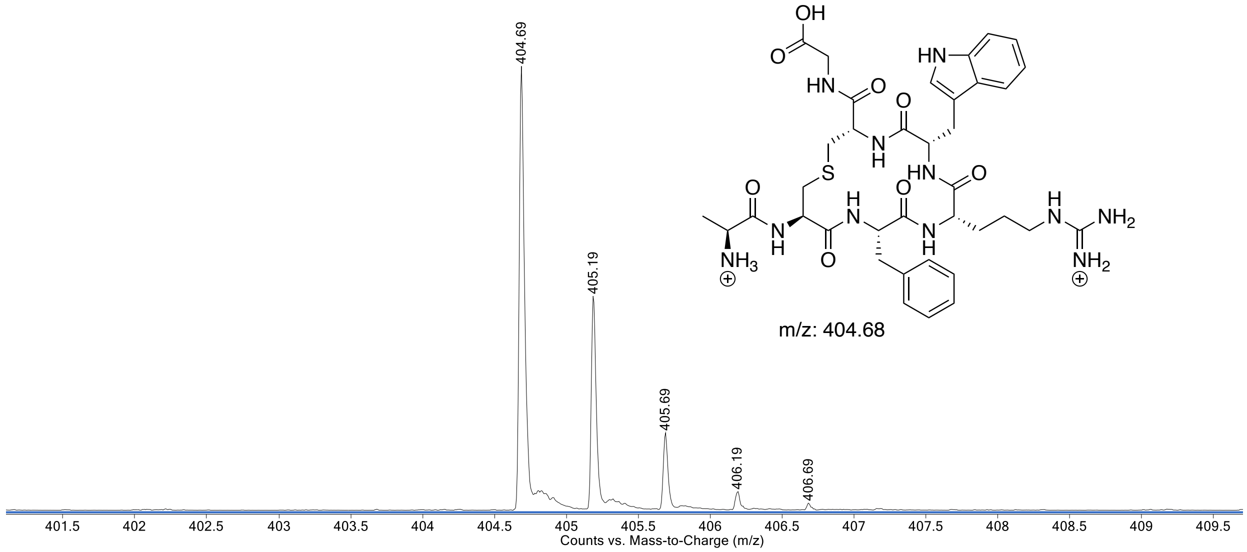

Figure S18: MS[1] spectrum demonstrating the isotopic distribution of the $[M+2H]^{2+}$ ions corresponding to macrocyclic MppE-W80F^{core} peptide (theoretical *m/z*: 404.68) following excision of MppE^{leader} peptide by LahT150.

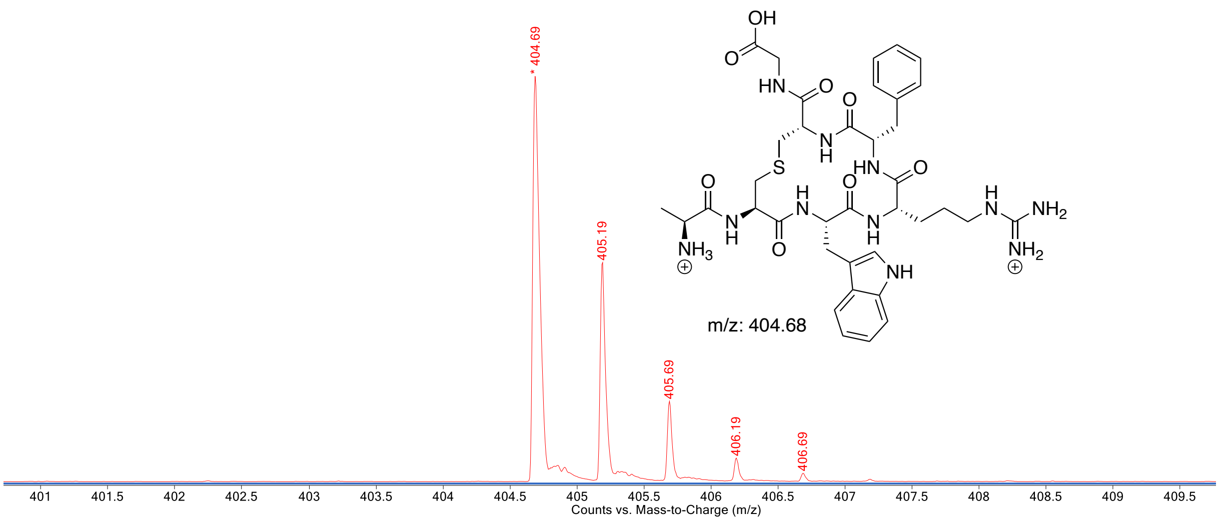

Figure S19: MS[1] spectrum demonstrating the isotopic distribution of the $[M+2H]^{2+}$ ions corresponding to macrocyclic MppE-W82F^{core} peptide (theoretical *m/z*: 404.68) following excision of MppE^{leader} peptide by LahT150.

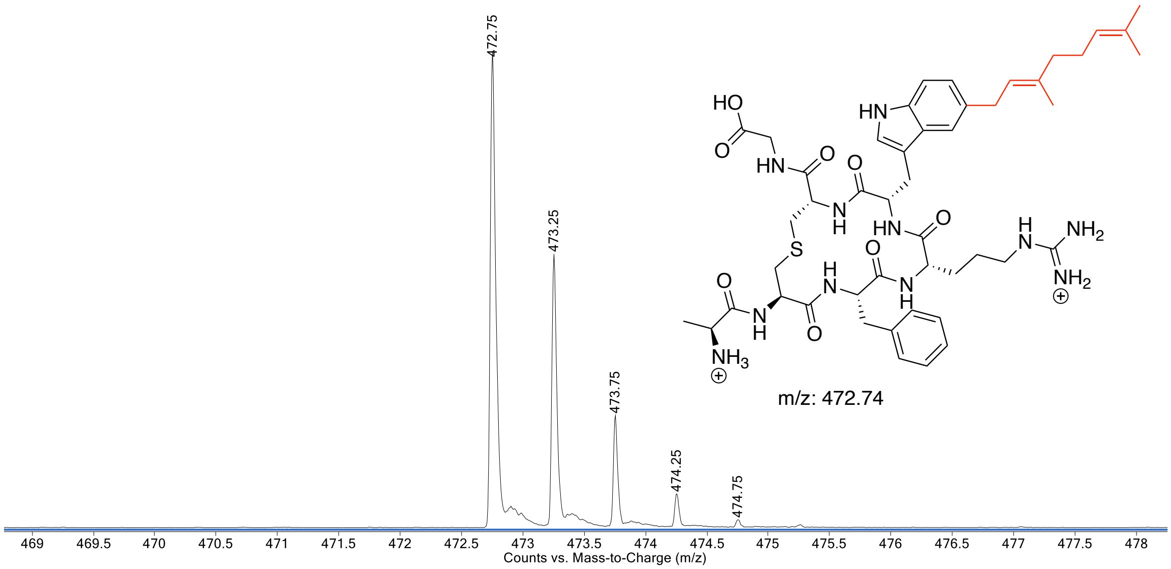

Figure S20: MS[1] spectrum demonstrating the isotopic distribution of the $[M+2H]^{2+}$ ions corresponding to C_{10}-prenylated macrocyclic MppE-W80F^{core} peptide (theoretical *m/z*: 472.74) following excision of MppE^{leader} peptide by LahT150.

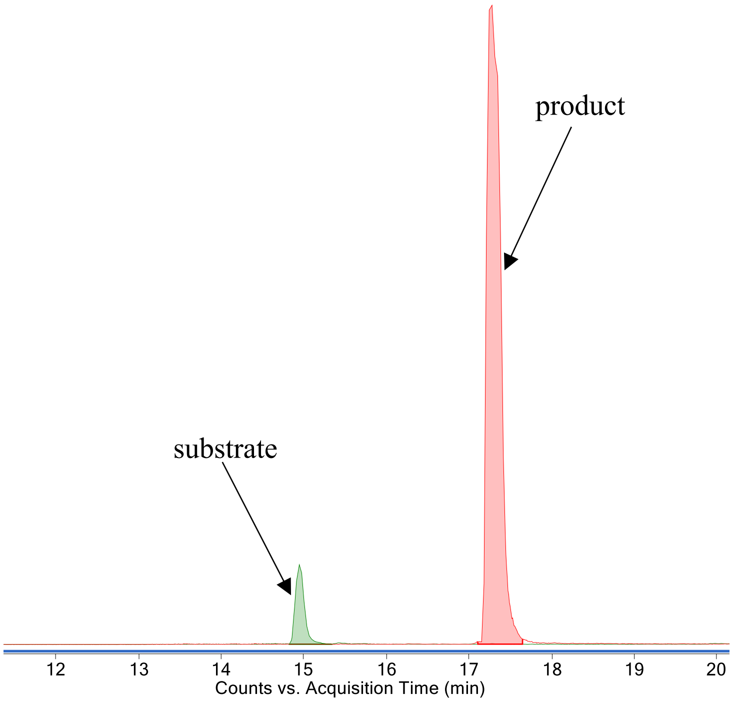

Figure S21: EICs for the most abundant $[M+2H]^{2+}$ ions corresponding to macrocyclic MppE-W80F^{core} peptide (in green) and C_{10}-prenylated macrocyclic MppE-W80F^{core} peptide (in red) when MppM-modified MppE-W80F substrate peptide was incubated with GPP and purified MppC.

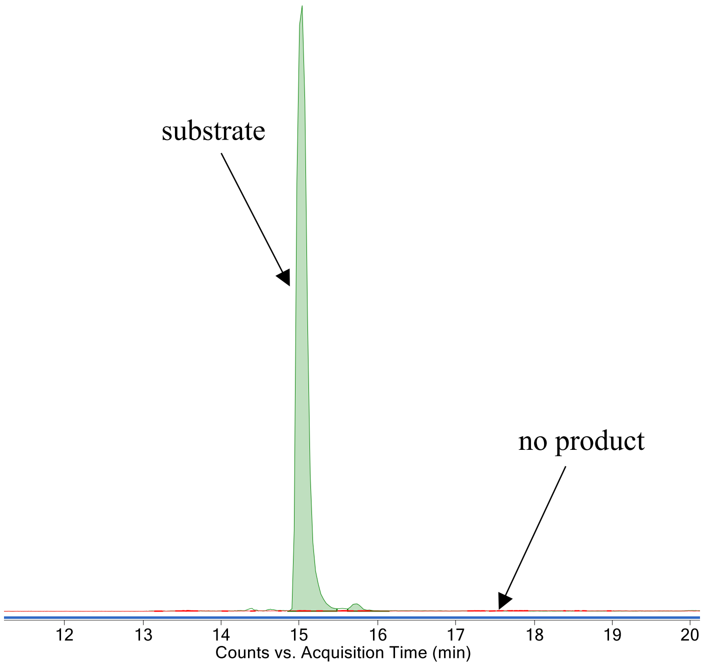

Figure S22: EICs for the most abundant $[M+2H]^{2+}$ ions corresponding to macrocyclic MppE-W82F^{core} peptide (in green) and C_{10}-prenylated macrocyclic MppE-W82F^{core} peptide (in red) when MppM-modified MppE-W82F substrate peptide was incubated with GPP and purified MppC.

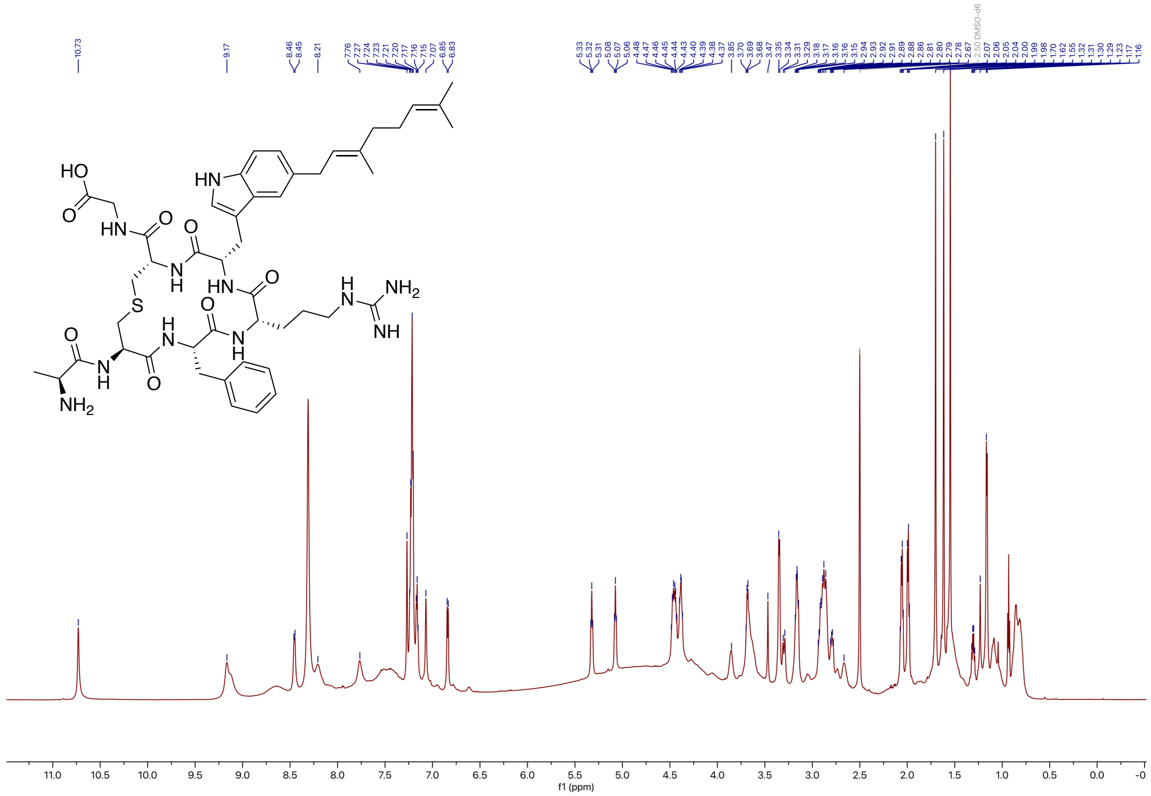

Figure S23: ^{1}H NMR spectrum of geranylated macrocyclic MppE-W80F^{core} peptide in DMSO-*d6*.

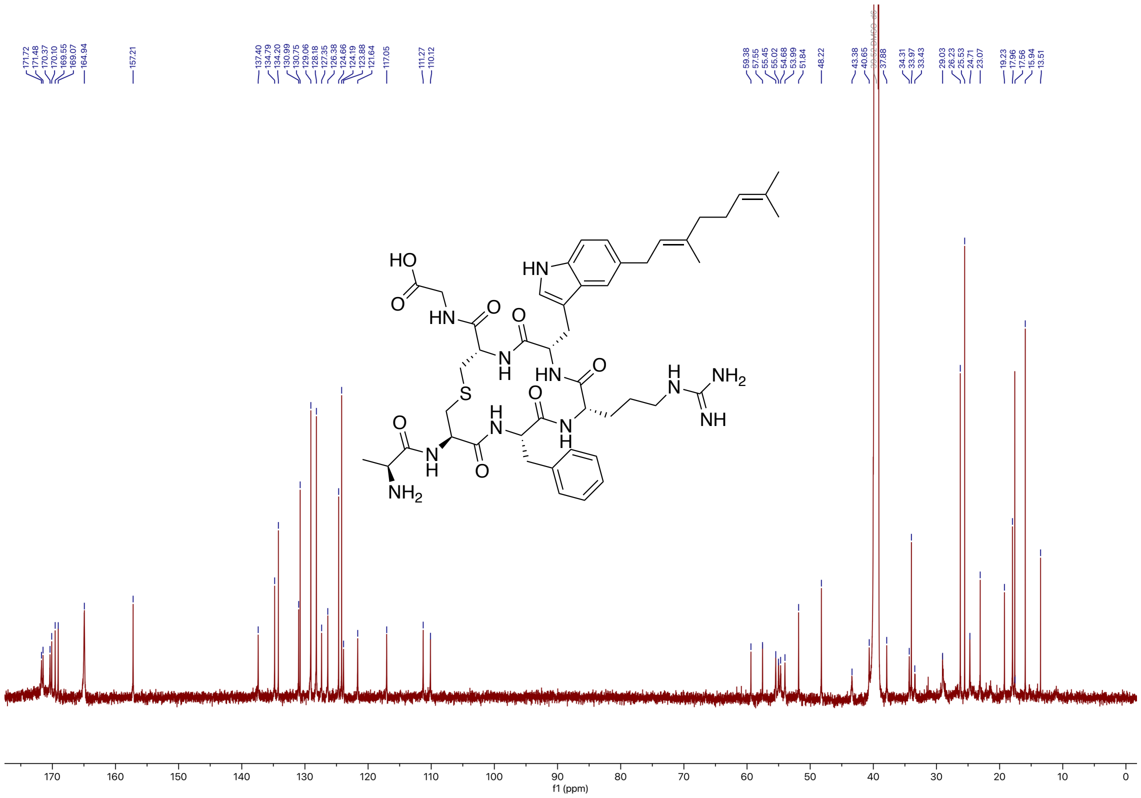

Figure S24: ^{13}C NMR spectrum of geranylated macrocyclic MppE-W80F^{core} peptide in DMSO-*d6*.

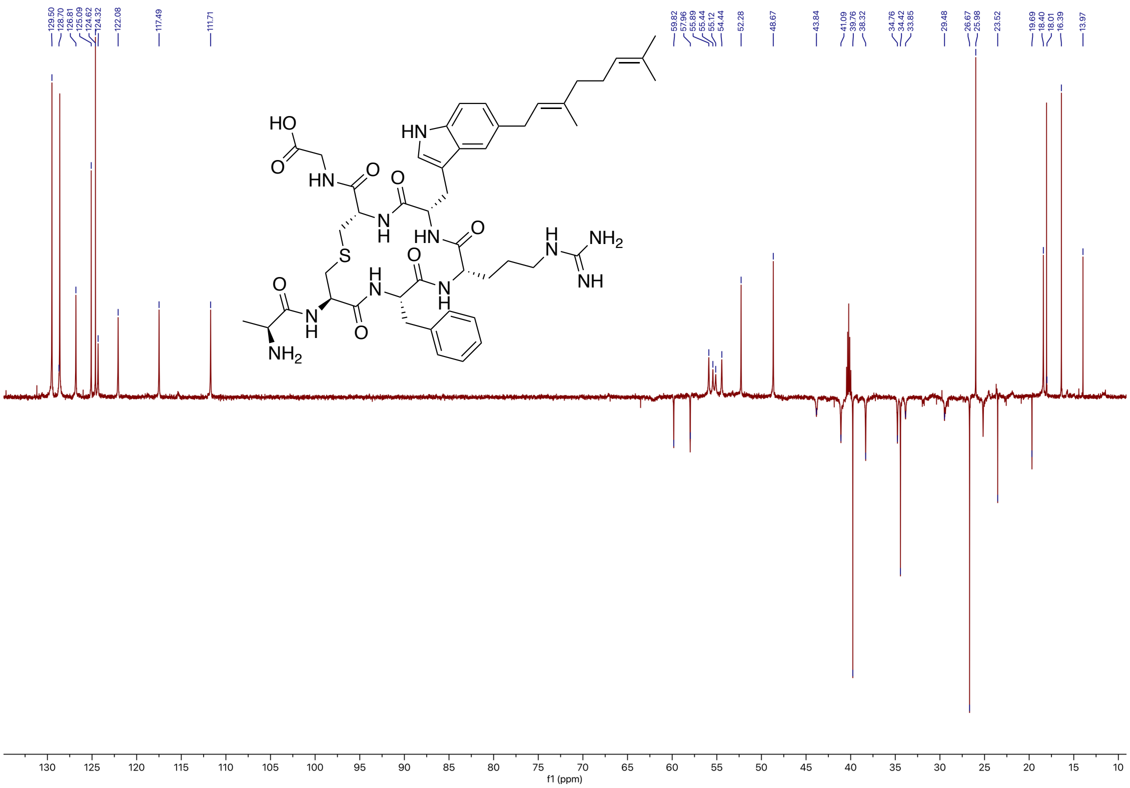

Figure S25: ^{13}C DEPT135 NMR spectrum of geranylated macrocyclic MppE-W80F^{core} peptide in DMSO-*d6*.

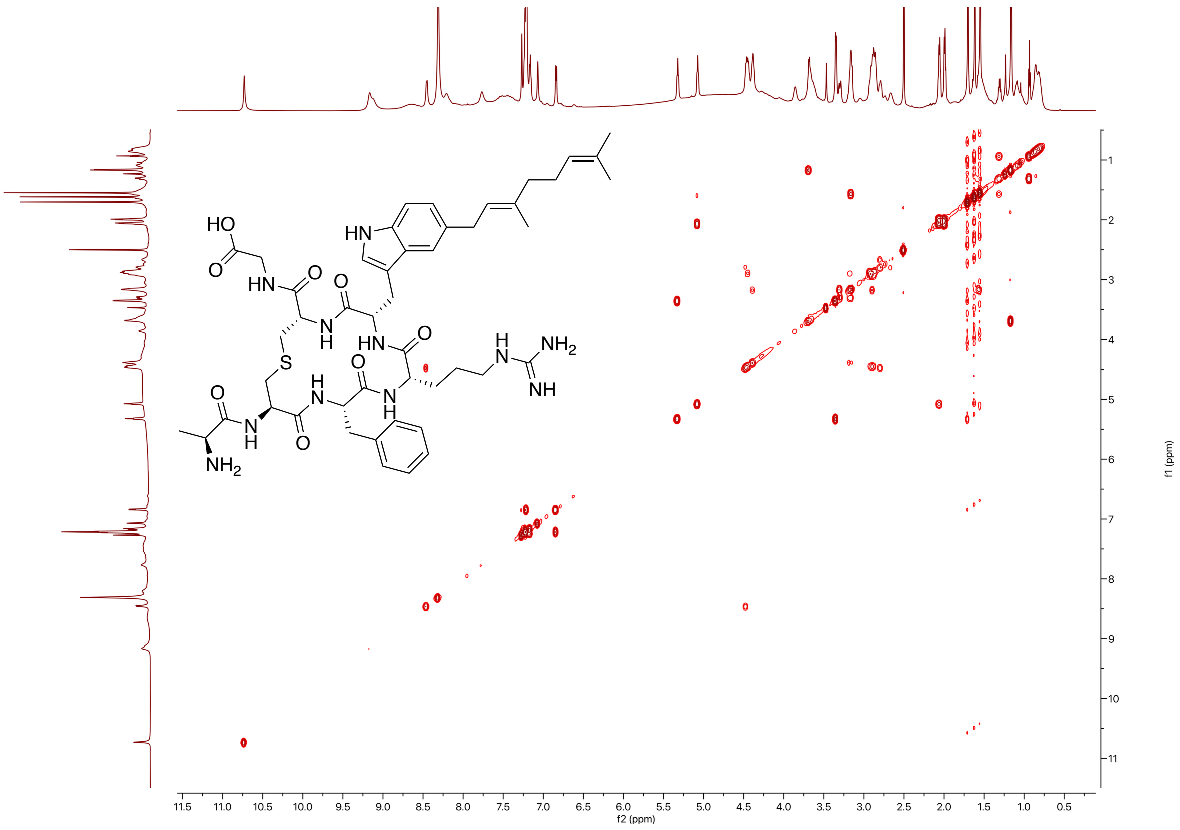

Figure S26: ^{1}H-^{1}H COSY NMR spectrum of geranylated macrocyclic MppE-W80F^{core} peptide in DMSO-*d6*.

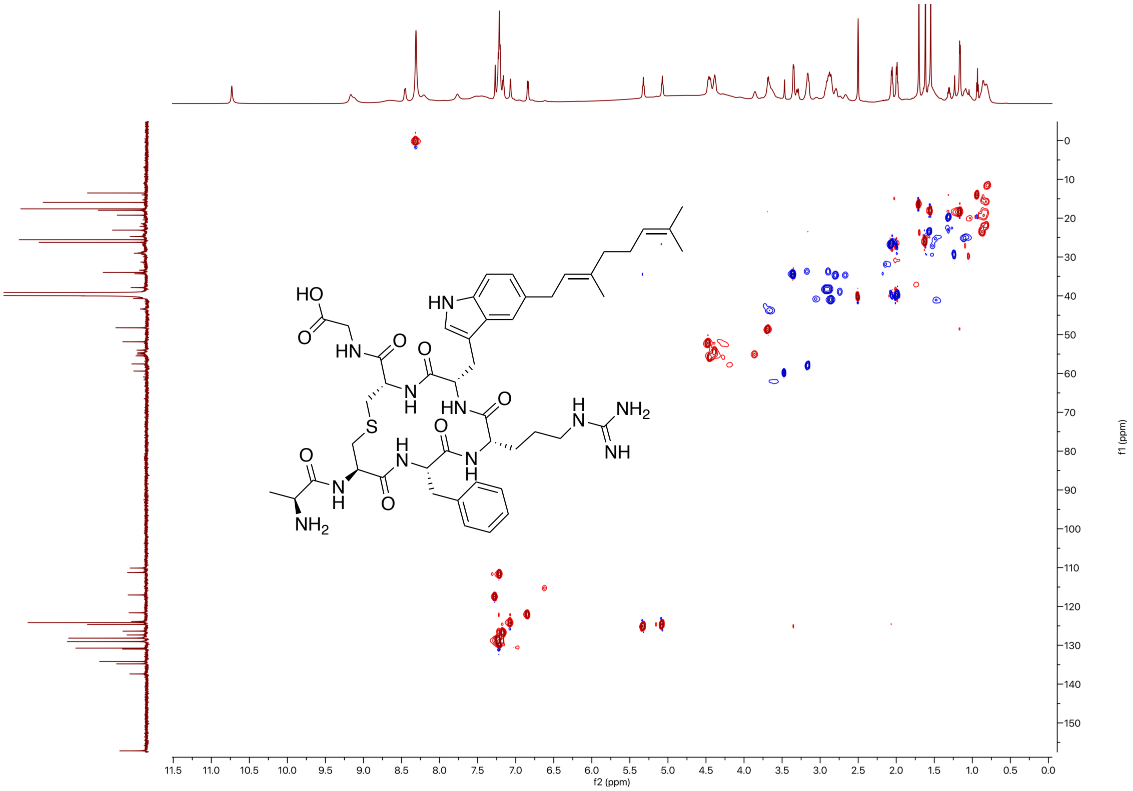

Figure S27: ^{1}H-^{13}C HSQC NMR spectrum of geranylated macrocyclic MppE-W80F^{core} peptide in DMSO-*d6*.

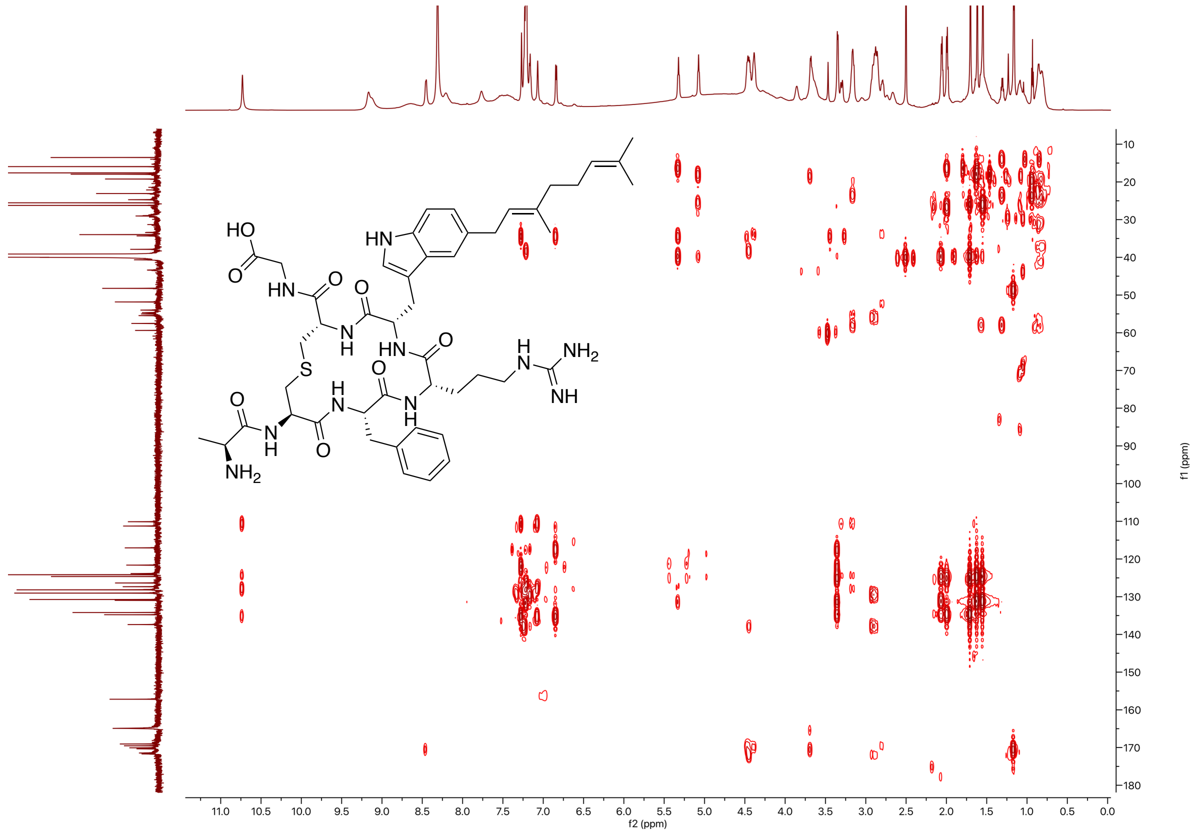

Figure S28: ^{1}H-^{13}C HMBC NMR spectrum of geranylated macrocyclic MppE-W80F^{core} peptide in DMSO-*d6*.

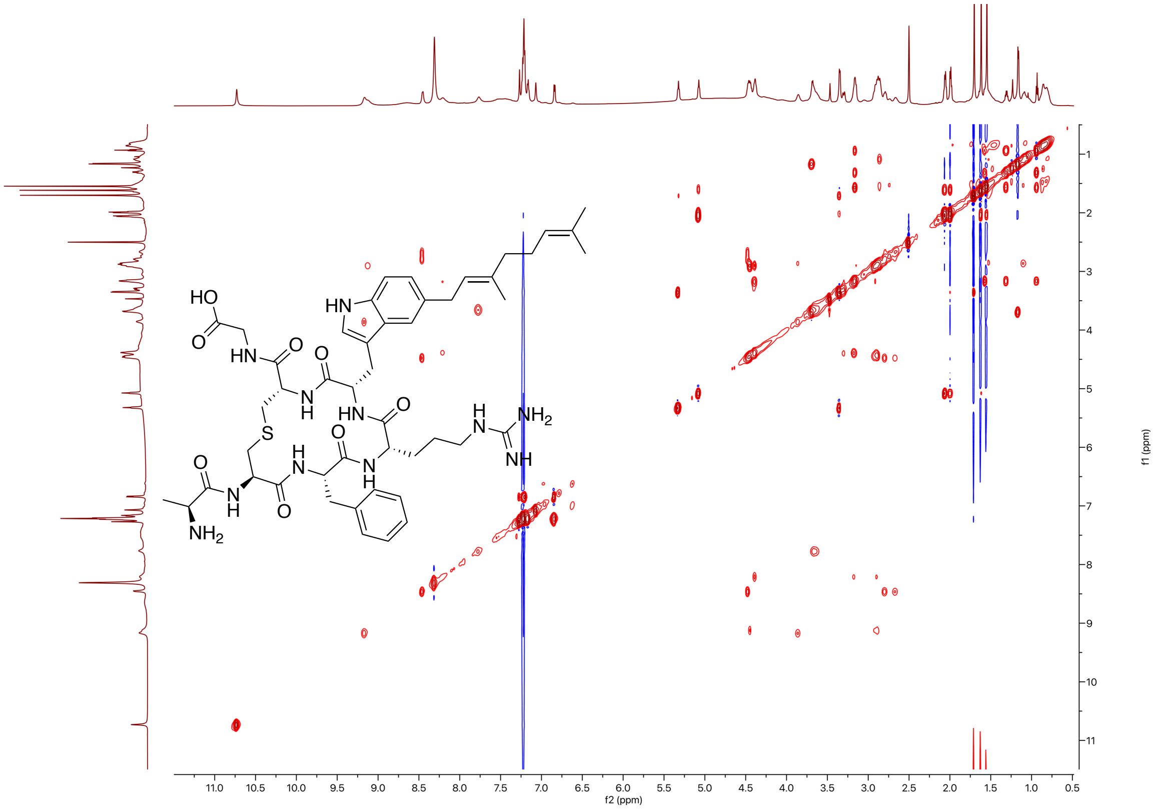

Figure S29: ^{1}H-^{1}H TOCSY NMR spectrum of geranylated macrocyclic MppE-W80F^{core} peptide in DMSO-*d6*.

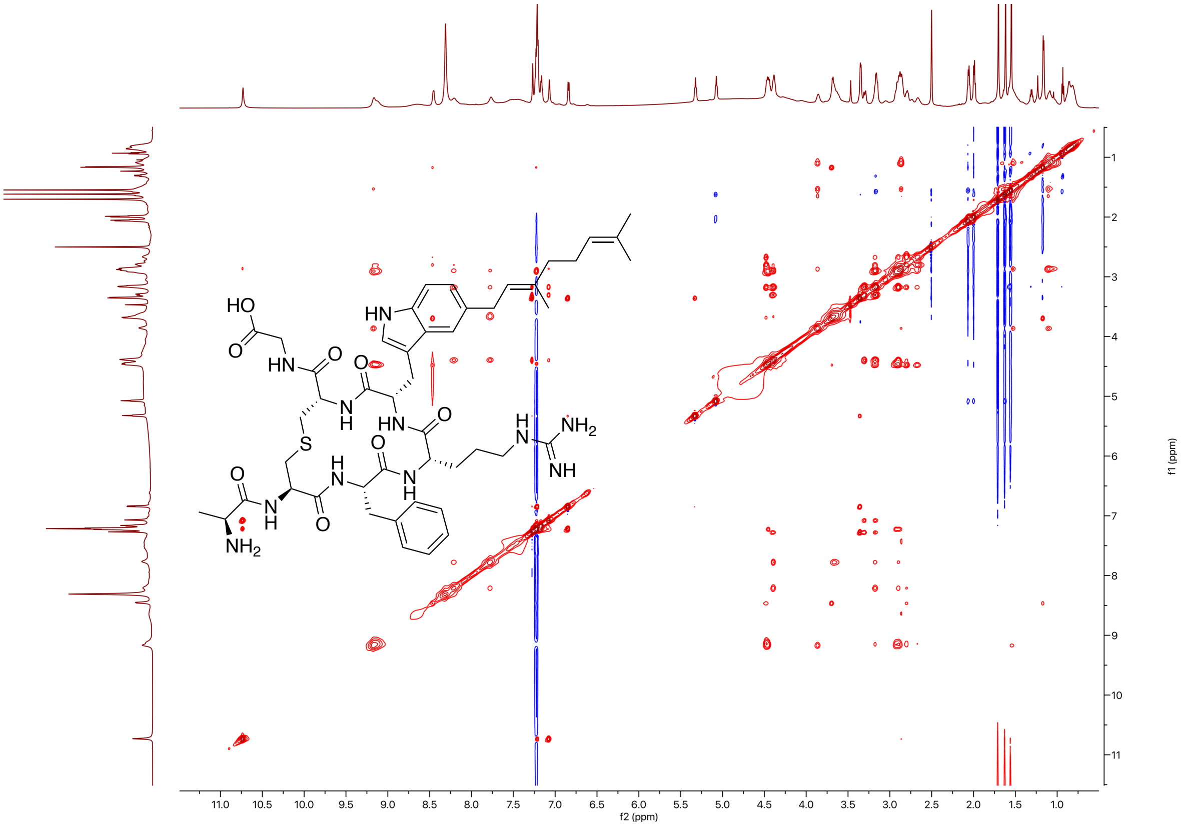

Figure S30: ^{1}H-^{1}H NOESY NMR spectrum of geranylated macrocyclic MppE-W80F^{core} peptide in DMSO-*d6*.

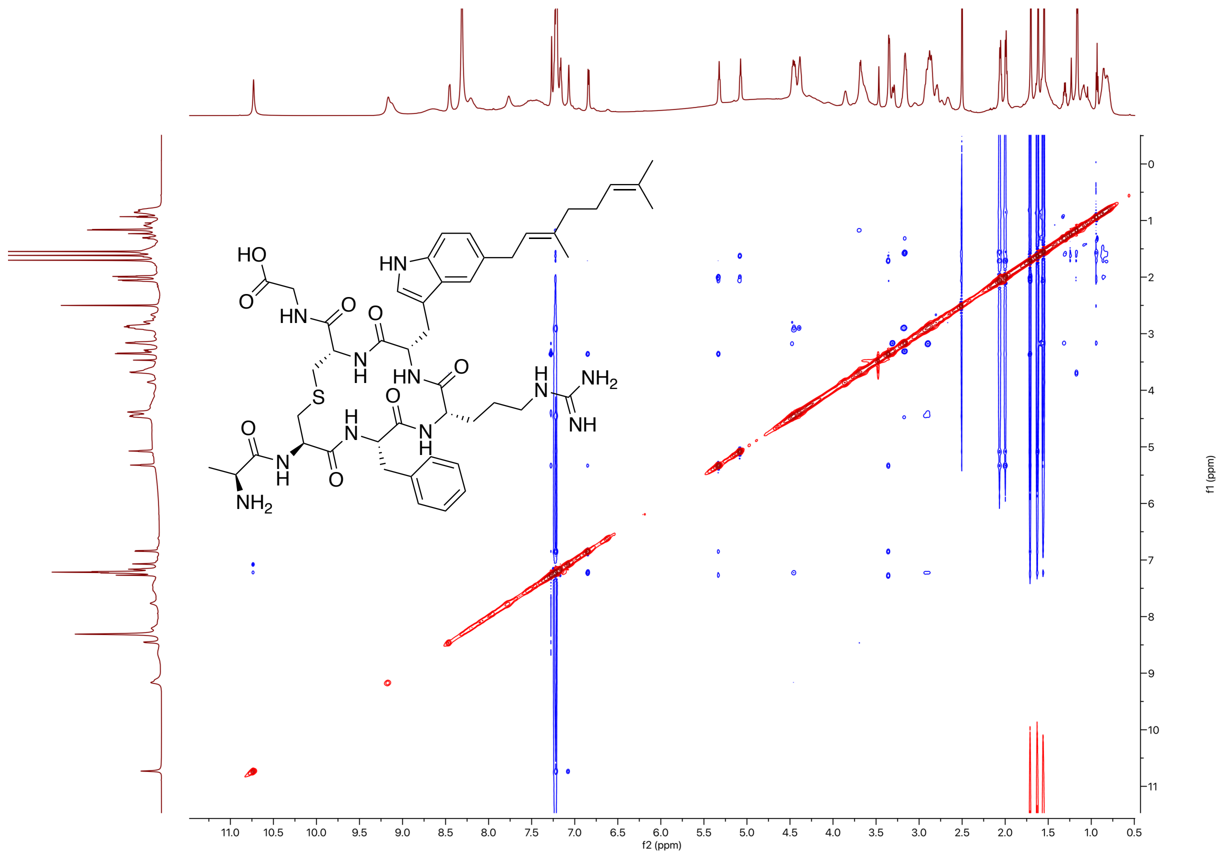

Figure S31: ${}^{1}H$-${}^{1}H$ ROESY NMR spectrum of geranylated macrocyclic MppE-W80F^{core} peptide in DMSO-*d6*.

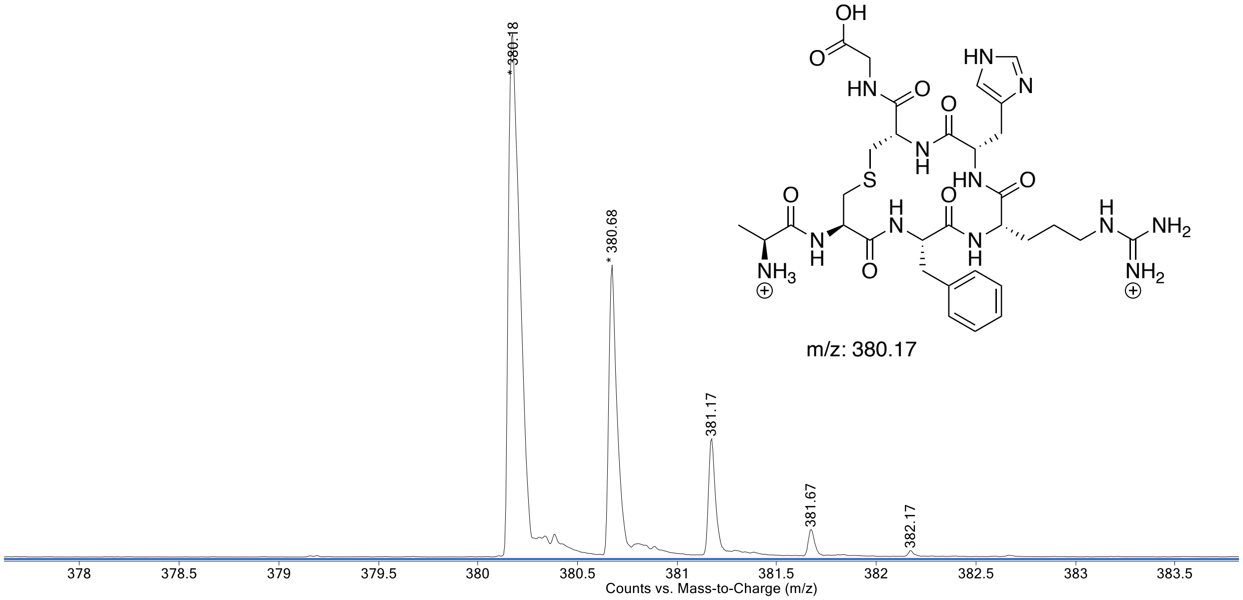

Figure S32: MS[1] spectrum demonstrating the isotopic distribution of the $[M+2H]^{2+}$ ions corresponding to macrocyclic MppE-W80F/W82H^{core} peptide (theoretical *m/z*: 380.17) following excision of MppE^{leader} peptide by LahT150.

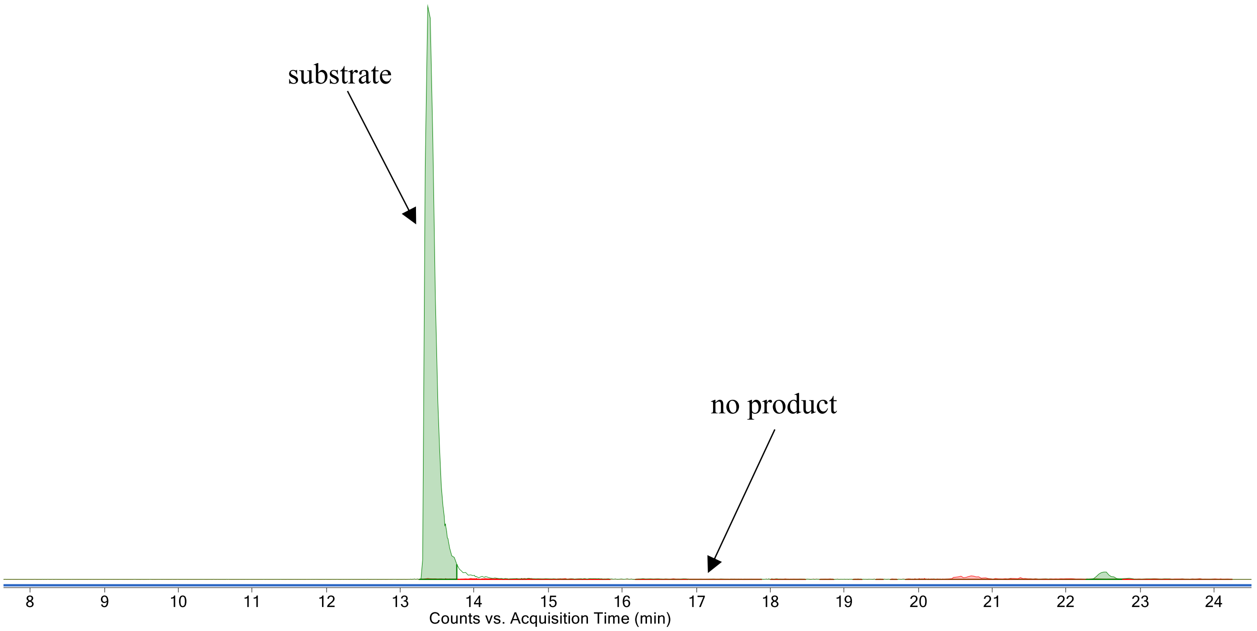

Figure S33: EICs for the most abundant $[M+2H]^{2+}$ ions corresponding to macrocyclic MppE-W80F/W82H^{core} peptide (in green) and C_{10}-prenylated macrocyclic MppE-W80F/W82H^{core} peptide (in red) when MppM-modified MppE-W80F/W82H substrate peptide was incubated with GPP and purified MppC at 30 °C for 18 h.

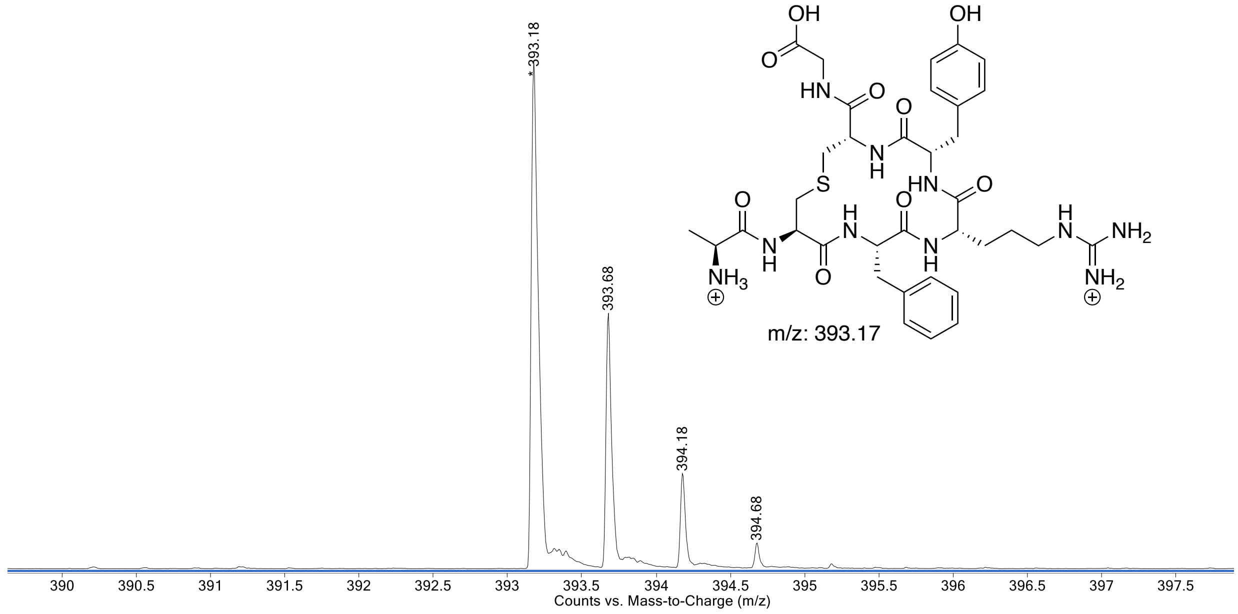

Figure S34: MS^1 spectrum demonstrating the isotopic distribution of the $[M+2H]^{2+}$ ions corresponding to macrocyclic MppE-W80F/W82Y^{core} peptide (theoretical *m/z*: 393.17) following excision of MppE^{leader} peptide by LahT150.

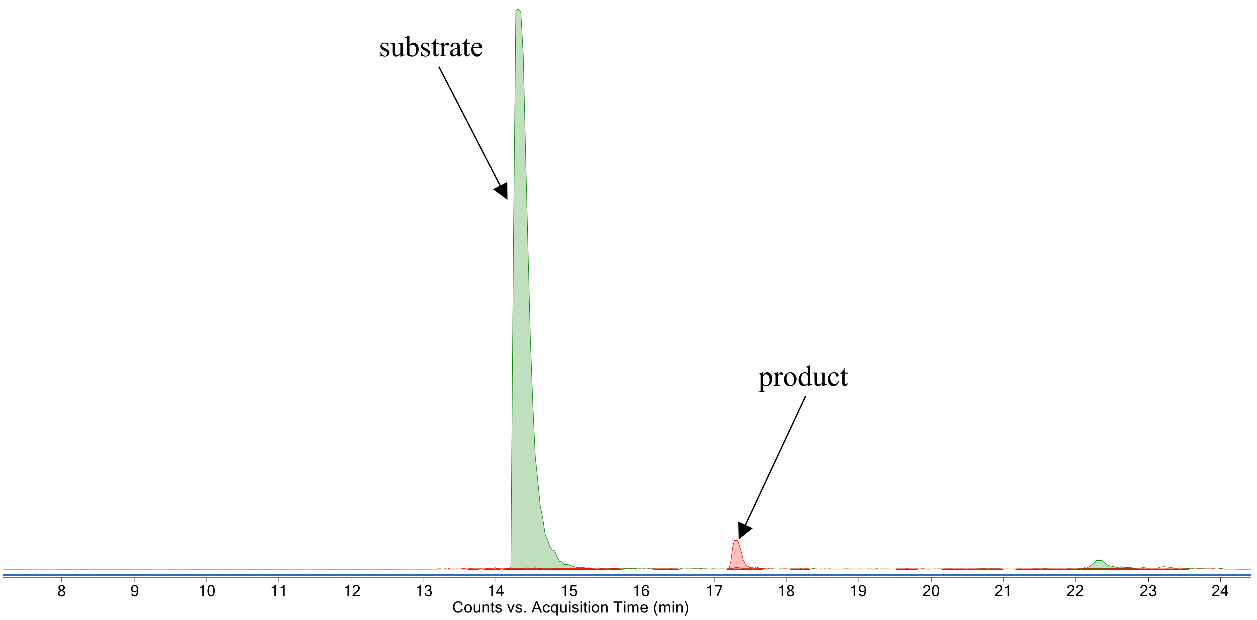

Figure S35: EICs for the most abundant $[M+2H]^{2+}$ ions corresponding to macrocyclic MppE-W80F/W82Y^{core} peptide (in green) and C_{10}-prenylated macrocyclic MppE-W80F/W82Y^{core} peptide (in red) when MppM-modified MppE-W80F/W82Y substrate peptide was incubated with GPP and purified MppC at 30 °C for 18 h.

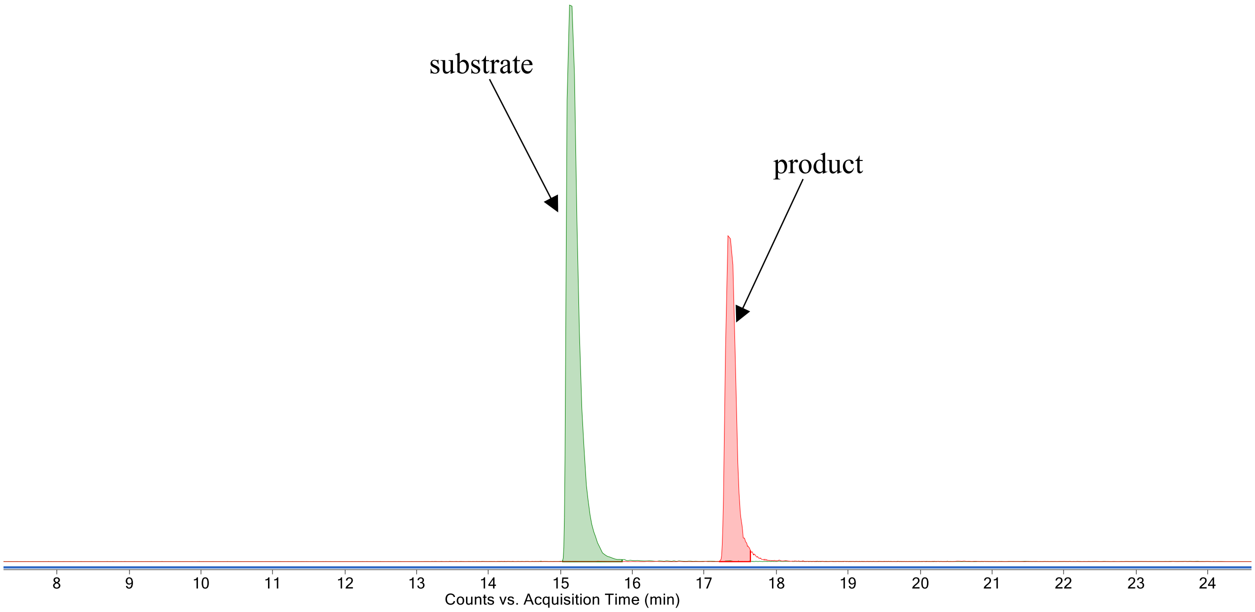

Figure S36: EICs for the most abundant $[M+2H]^{2+}$ ions corresponding to macrocyclic MppE^{core} peptide (in green) and C_{10}-prenylated macrocyclic MppE^{core} peptide (in red) when MppM-modified MBP-MppE substrate was incubated with GPP and purified MppC at 30 °C for 0.5 h. Note that MppE wild-type and (Δ1–55)MppE were produced as MBP-fused proteins to ensure the solubility of (Δ1–55)MppE and maintain similar conditions for MppE wild-type.

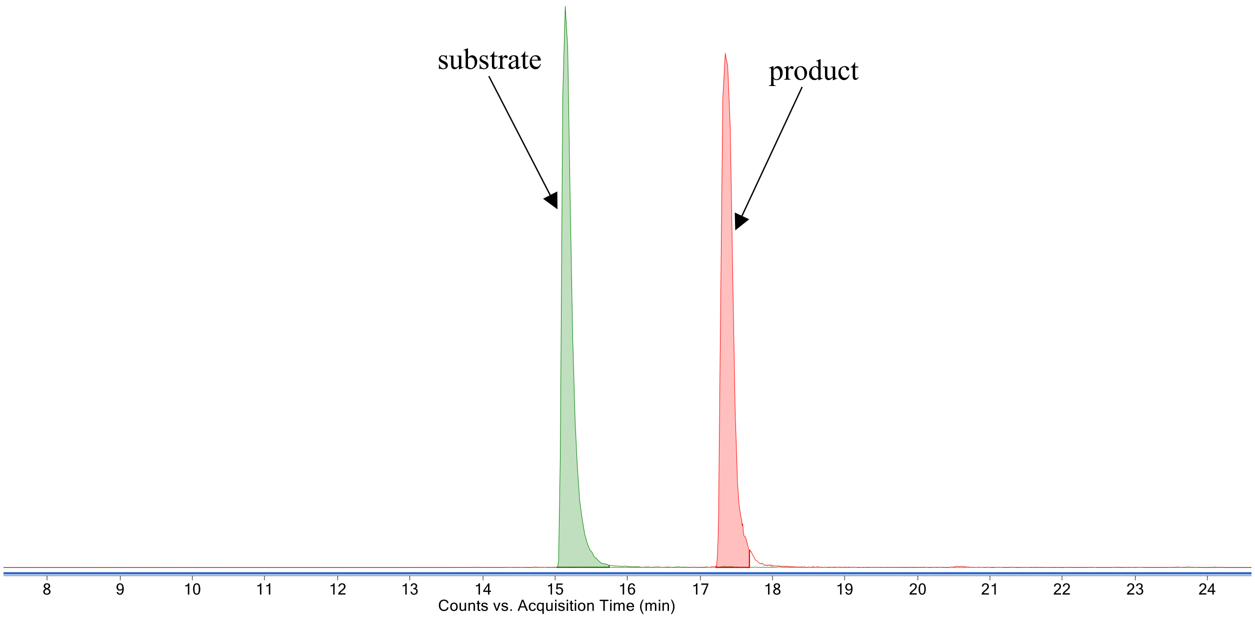

Figure S37: EICs for the most abundant $[M+2H]^{2+}$ ions corresponding to macrocyclic MppE^{core} peptide (in green) and C_{10}-prenylated macrocyclic MppE^{core} peptide (in red) when MppM-modified MBP-MppE substrate was incubated with GPP and purified MppC at 30 °C for 1 h.

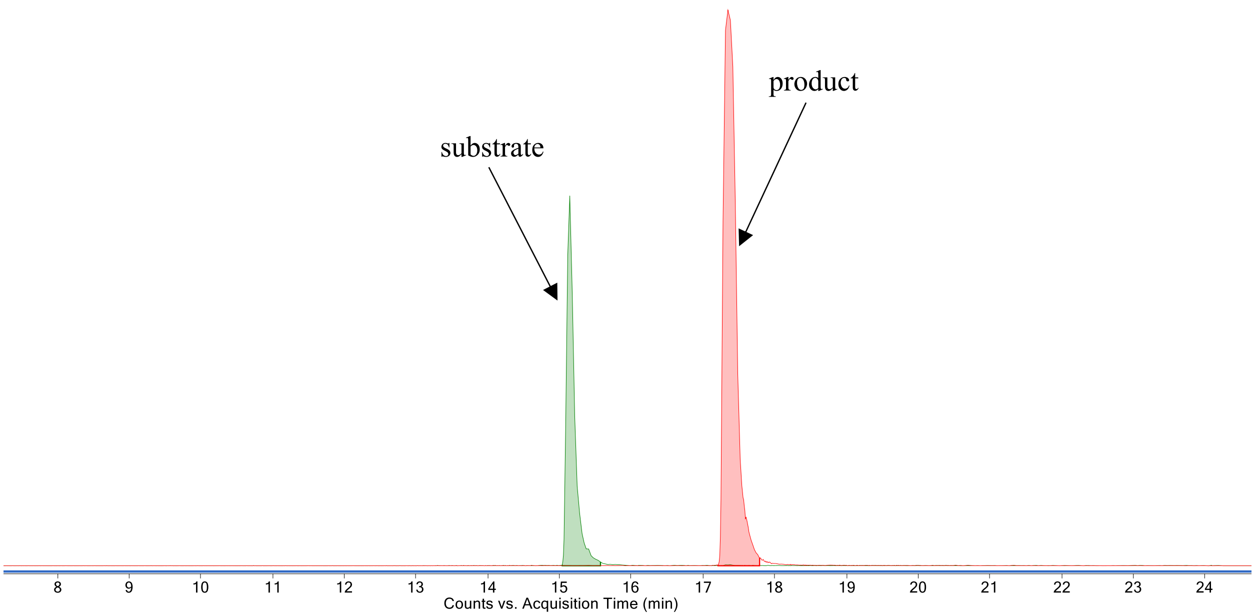

Figure S38: EICs for the most abundant $[M+2H]^{2+}$ ions corresponding to macrocyclic $MppE^{core}$ peptide (in green) and C_{10}-prenylated macrocyclic $MppE^{core}$ peptide (in red) when MppM-modified MBP-MppE substrate was incubated with GPP and purified MppC at 30 °C for 1.5 h

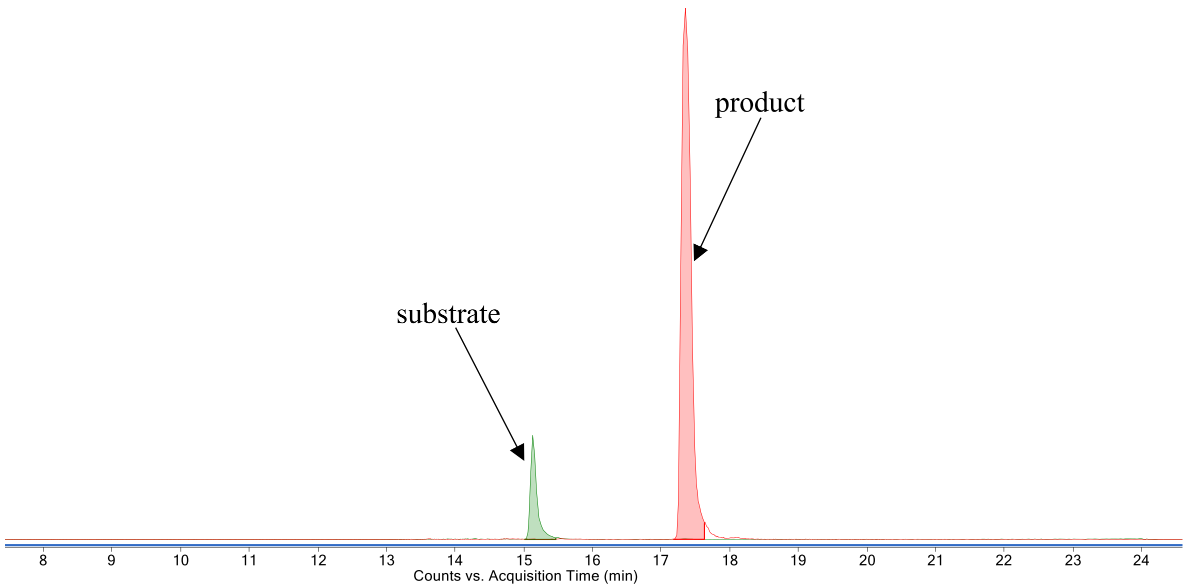

Figure S39: EICs for the most abundant $[M+2H]^{2+}$ ions corresponding to macrocyclic MppE^{core} peptide (in green) and C_{10}-prenylated macrocyclic MppE^{core} peptide (in red) when MppM-modified MBP-MppE substrate was incubated with GPP and purified MppC at 30 °C for 2 h.

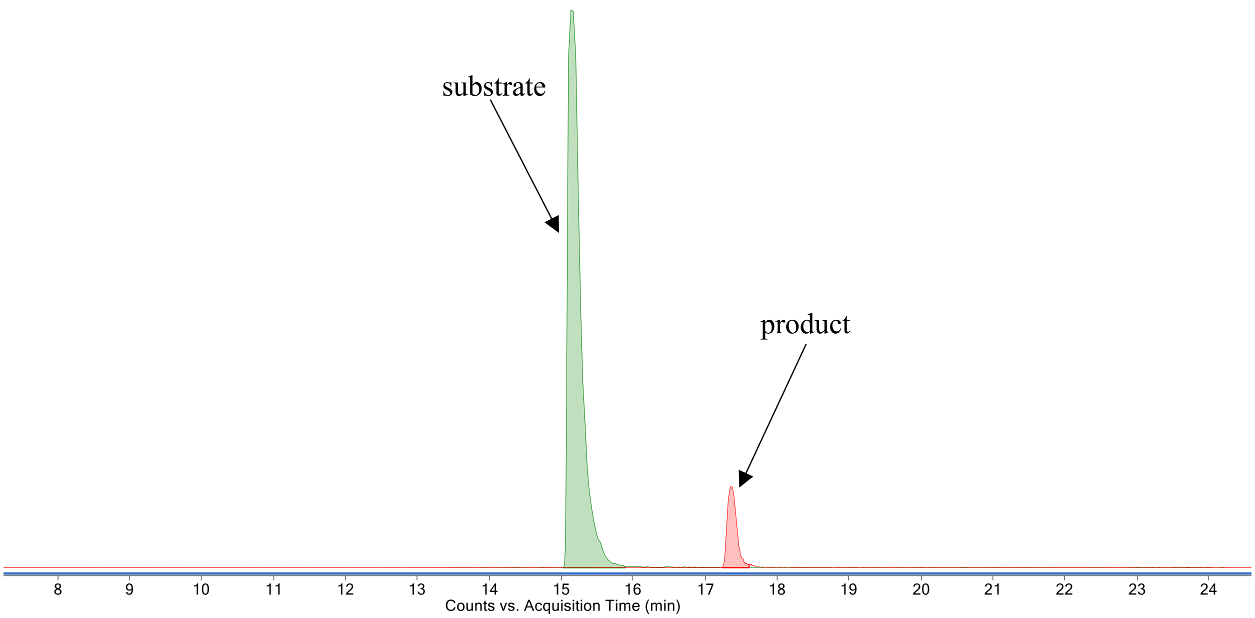

Figure S40: EICs for the most abundant $[M+2H]^{2+}$ ions corresponding to macrocyclic MppE^{core} peptide (in green) and C_{10}-prenylated macrocyclic MppE^{core} peptide (in red) when MppM-modified MBP-(Δ1–55)MppE substrate was incubated with GPP and purified MppC at 30 °C for 0.5 h.

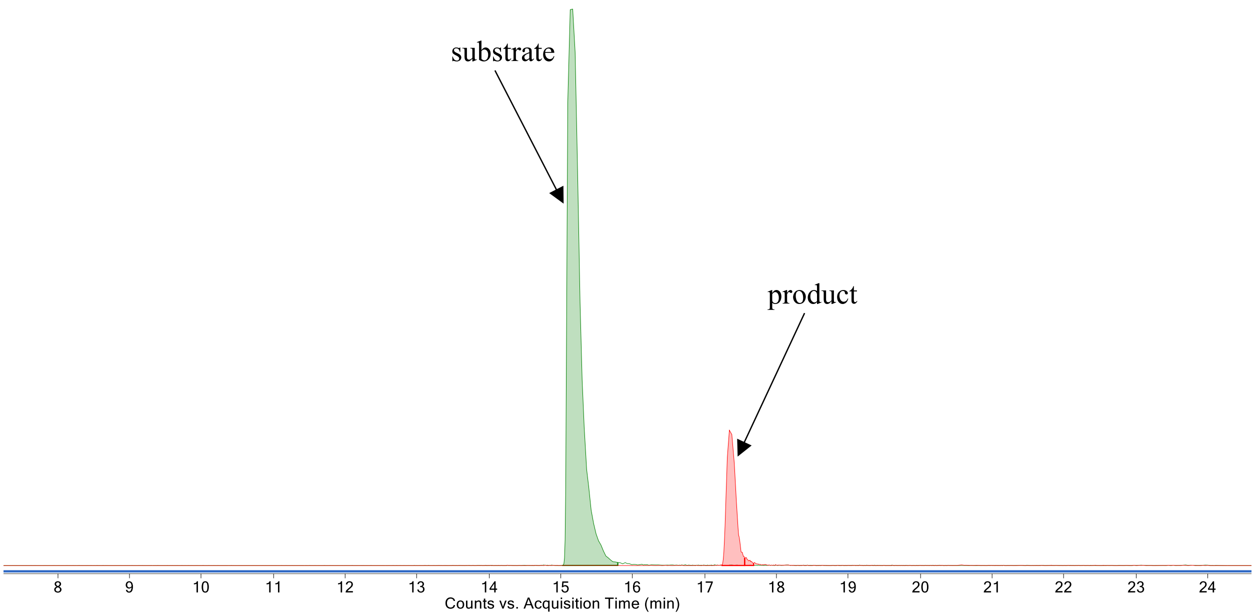

Figure S41: EICs for the most abundant $[M+2H]^{2+}$ ions corresponding to macrocyclic MppE^{core} peptide (in green) and C_{10}-prenylated macrocyclic MppE^{core} peptide (in red) when MppM-modified MBP-(Δ1–55)MppE substrate was incubated with GPP and purified MppC at 30 °C for 1 h.

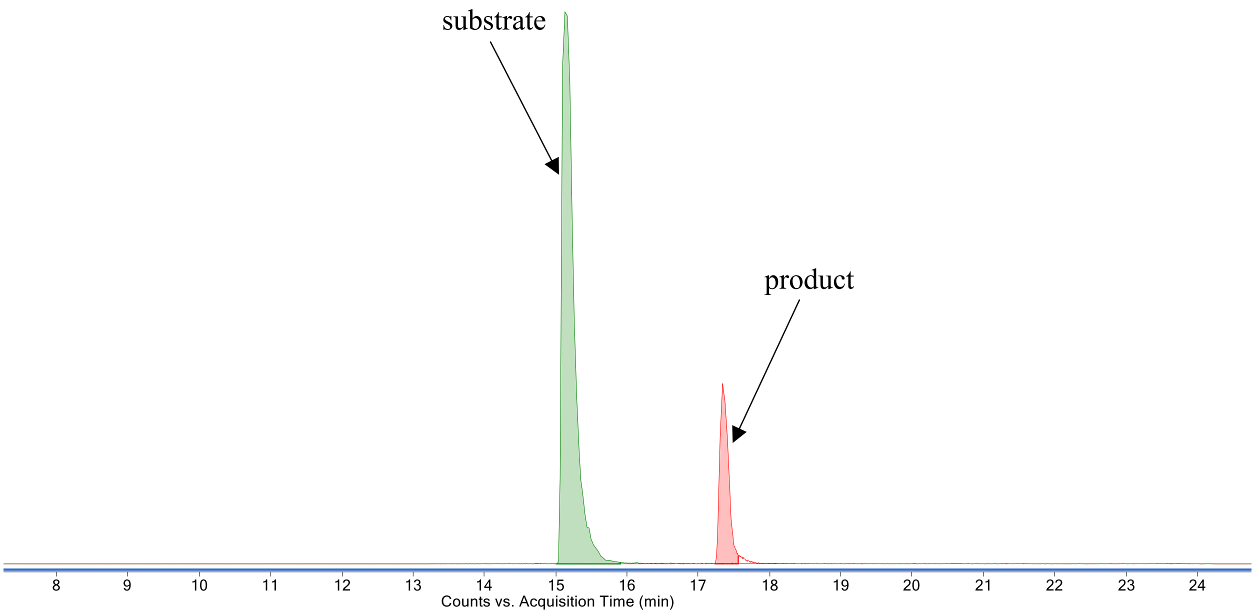

Figure S42: EICs for the most abundant $[M+2H]^{2+}$ ions corresponding to macrocyclic MppE^{core} peptide (in green) and C_{10}-prenylated macrocyclic MppE^{core} peptide (in red) when MppM-modified MBP-(Δ1–55)MppE substrate was incubated with GPP and purified MppC at 30 °C for 1.5 h.

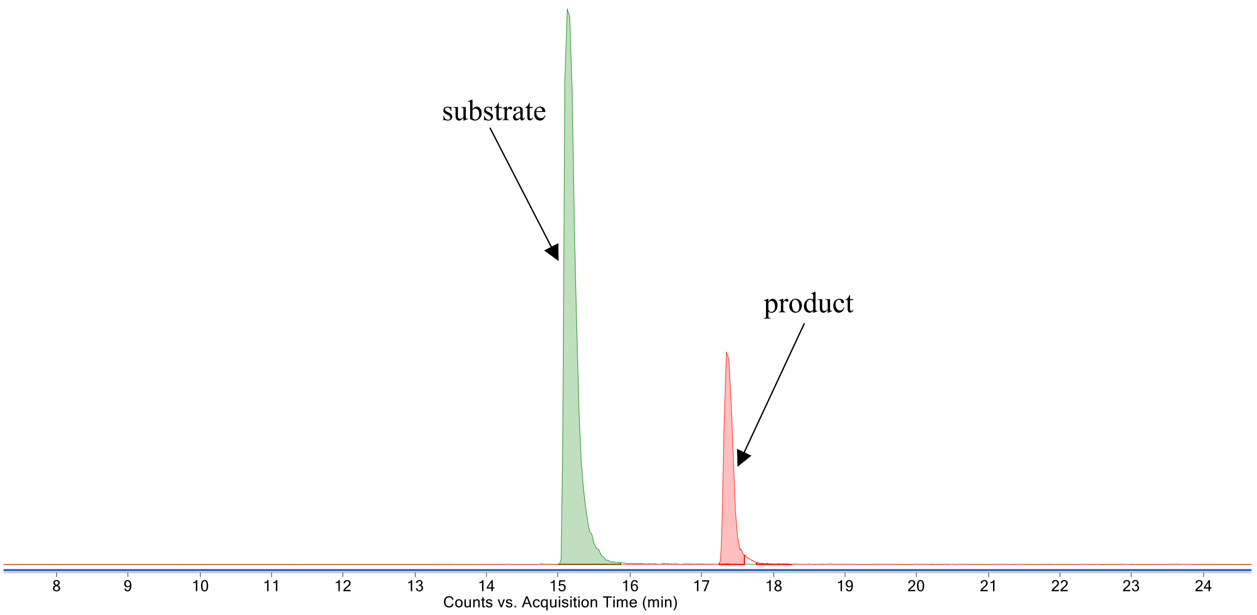

Figure S43: EICs for the most abundant $[M+2H]^{2+}$ ions corresponding to macrocyclic MppE^{core} peptide (in green) and C_{10}-prenylated macrocyclic MppE^{core} peptide (in red) when MppM-modified MBP-(Δ1–55)MppE substrate was incubated with GPP and purified MppC at 30 °C for 2 h.

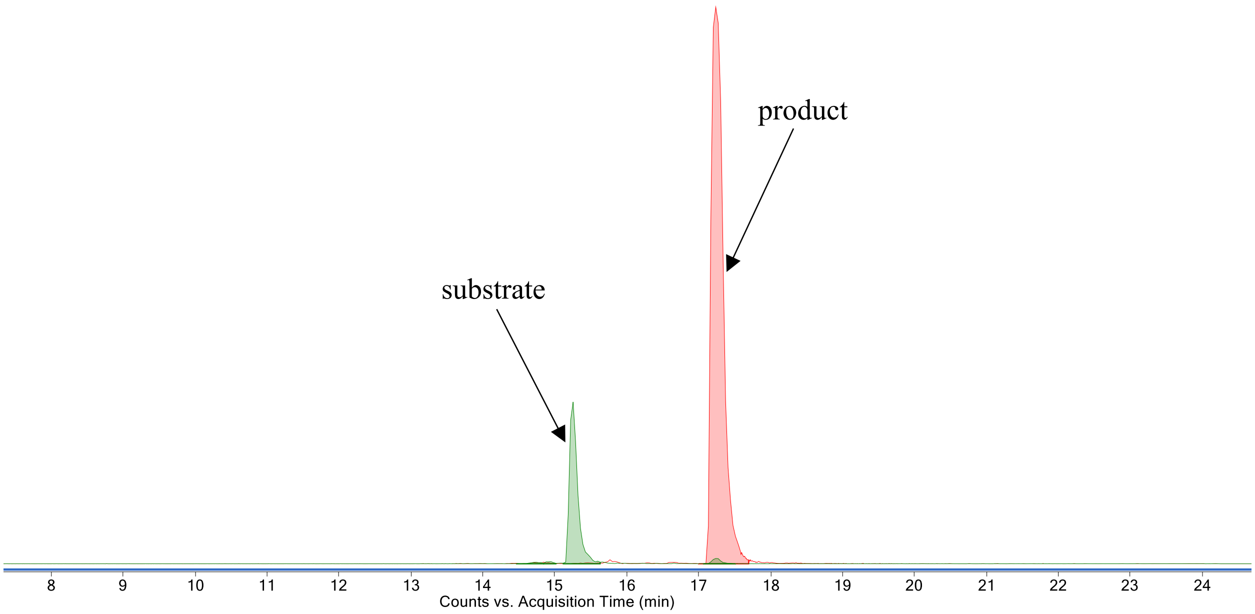

Figure S44: EICs for the most abundant $[M+2H]^{2+}$ ions corresponding to macrocyclic MppE^{core} peptide (in green) and C_{10}-prenylated macrocyclic MppE^{core} peptide (in red) when MppM-modified MppE wild-type substrate peptide was incubated with GPP and purified MppC. Here, the leader peptides of MppE wild-type and MppE-L(X)$_4$L mutants were excised by Glu-C protease.

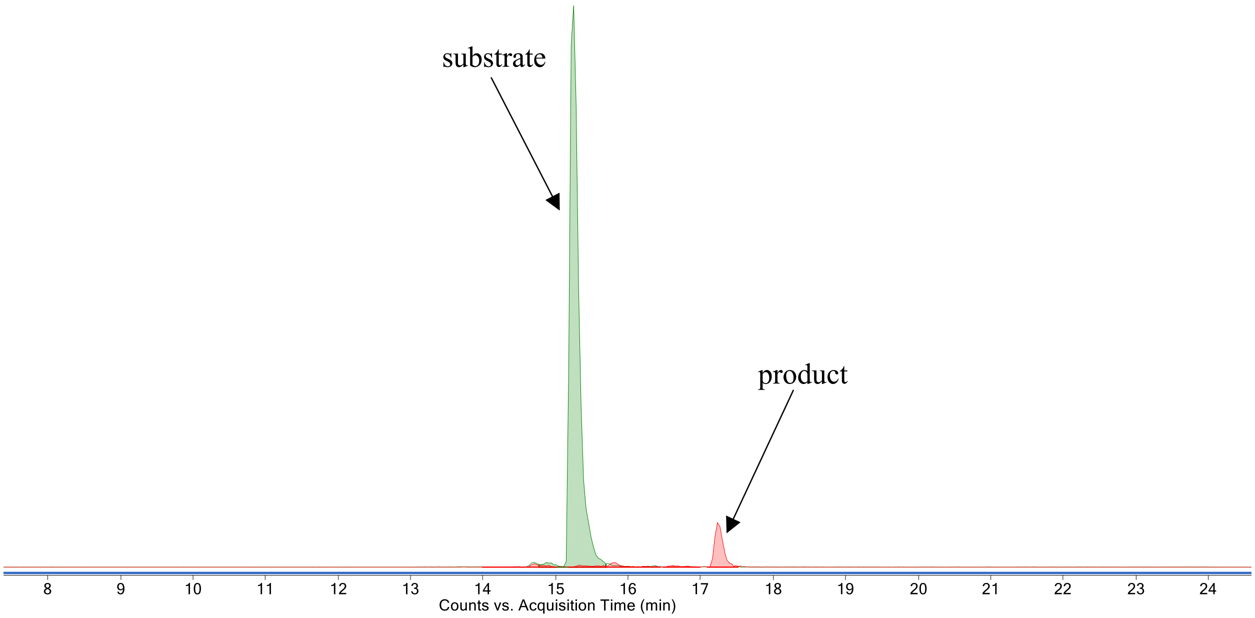

Figure S45: EICs for the most abundant $[M+2H]^{2+}$ ions corresponding to macrocyclic MppE^{core} peptide (in green) and C_{10}-prenylated macrocyclic MppE^{core} peptide (in red) when MppM-modified MppE-A$(X)_4$A substrate peptide was incubated with GPP and purified MppC.

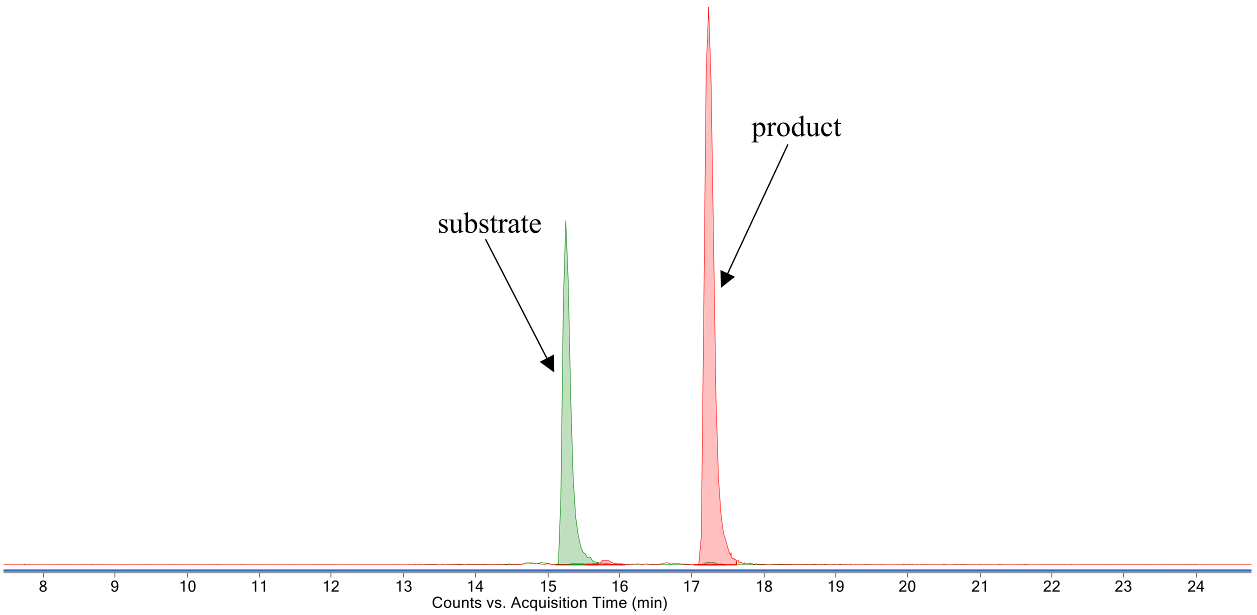

Figure S46: EICs for the most abundant $[M+2H]^{2+}$ ions corresponding to macrocyclic MppE^{core} peptide (in green) and C_{10}-prenylated macrocyclic MppE^{core} peptide (in red) when MppM-modified MppE-F(X)$_4$F substrate peptide was incubated with GPP and purified MppC.

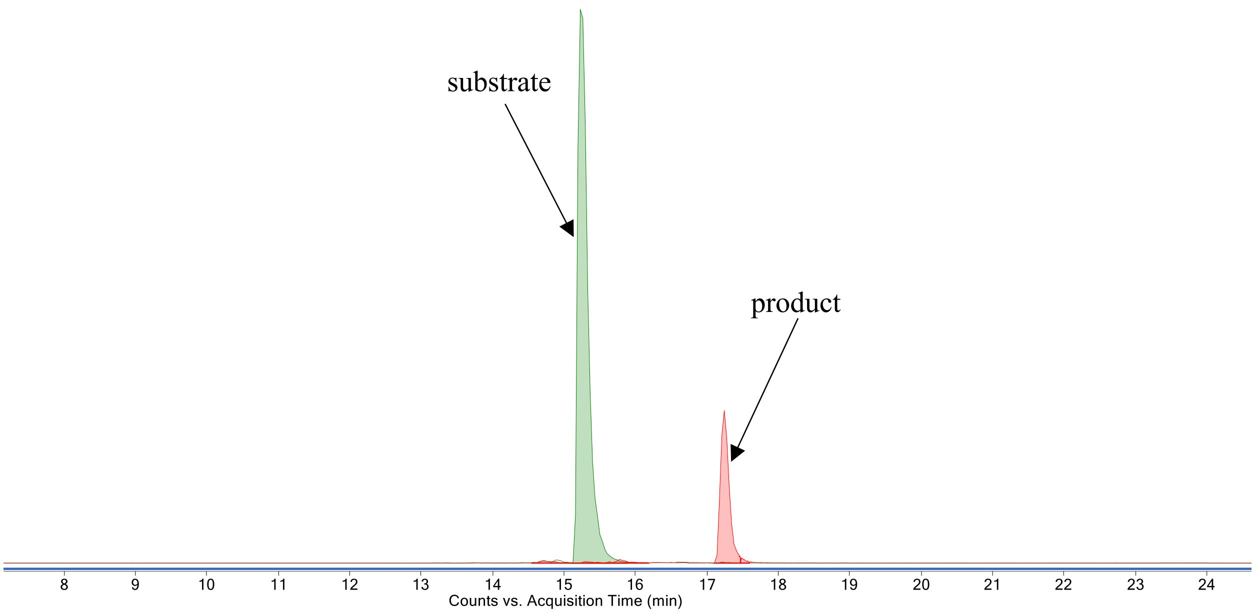

Figure S47: EICs for the most abundant $[M+2H]^{2+}$ ions corresponding to macrocyclic MppE^{core} peptide (in green) and C_{10}-prenylated macrocyclic MppE^{core} peptide (in red) when MppM-modified MppE-V(X)$_4$V substrate peptide was incubated with GPP and purified MppC.

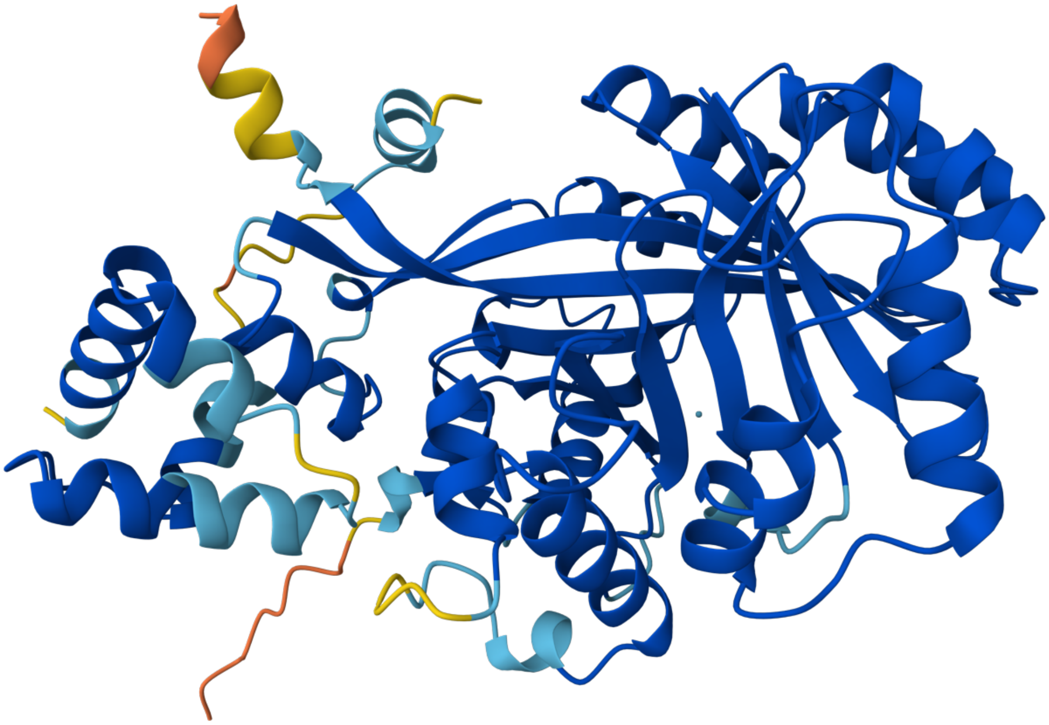

Figure S48: AlphaFold 3 model of MppC/MppE^{leader} complex in the presence of a Mg^{2+} (colored by pLDDT score, regions of low confidence colored in yellow and orange).

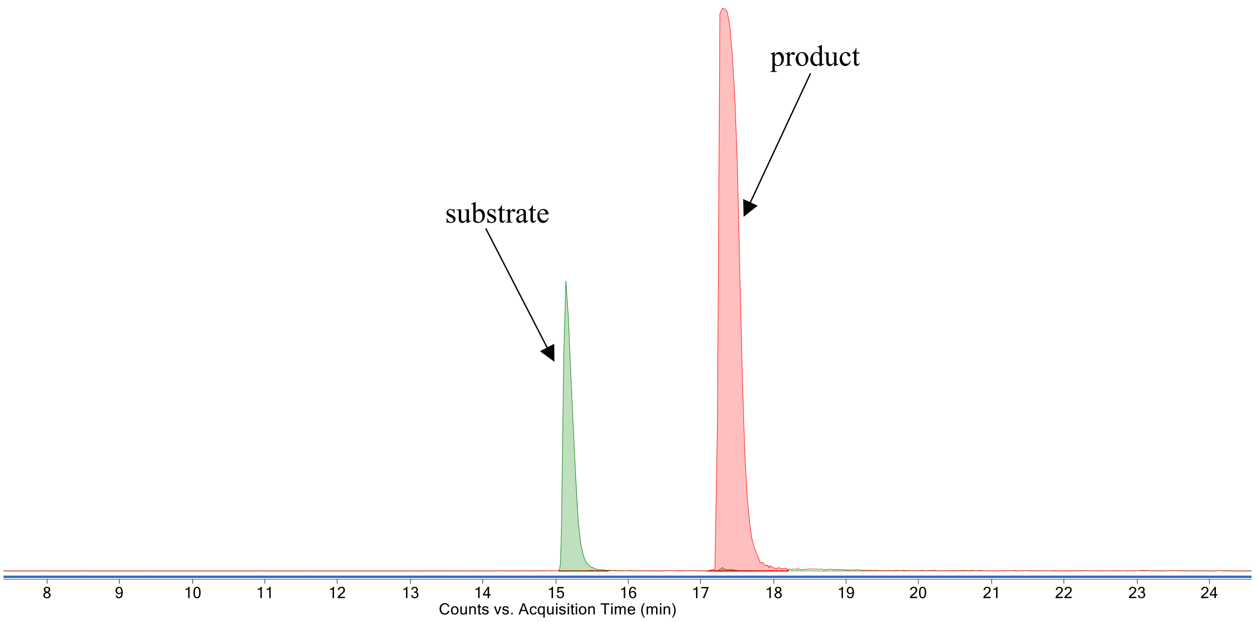

Figure S49: EICs for the most abundant $[M+2H]^{2+}$ ions corresponding to macrocyclic MppE^{core} peptide (in green) and C_{10}-prenylated macrocyclic MppE^{core} peptide (in red) when MppM-modified MppE substrate peptide was incubated with GPP and purified MppC wild-type at 30 °C for 0.5 h.

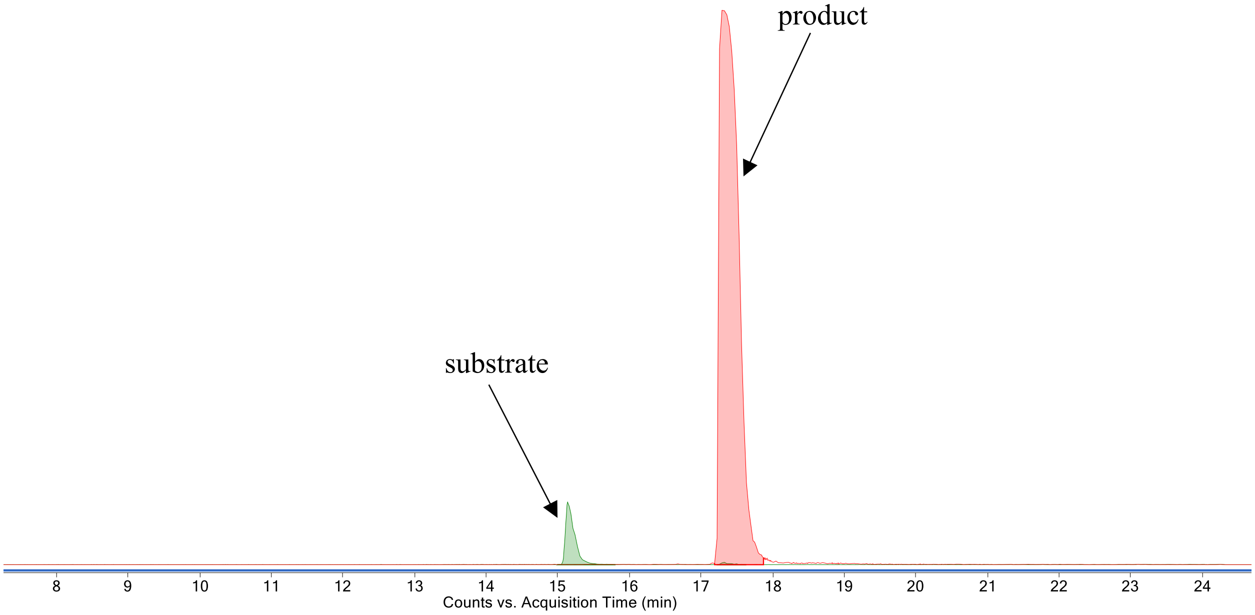

Figure S50: EICs for the most abundant $[M+2H]^{2+}$ ions corresponding to macrocyclic MppE^{core} peptide (in green) and C_{10}-prenylated macrocyclic MppE^{core} peptide (in red) when MppM-modified MppE substrate peptide was incubated with GPP and purified MppC wild-type at 30 °C for 1 h.

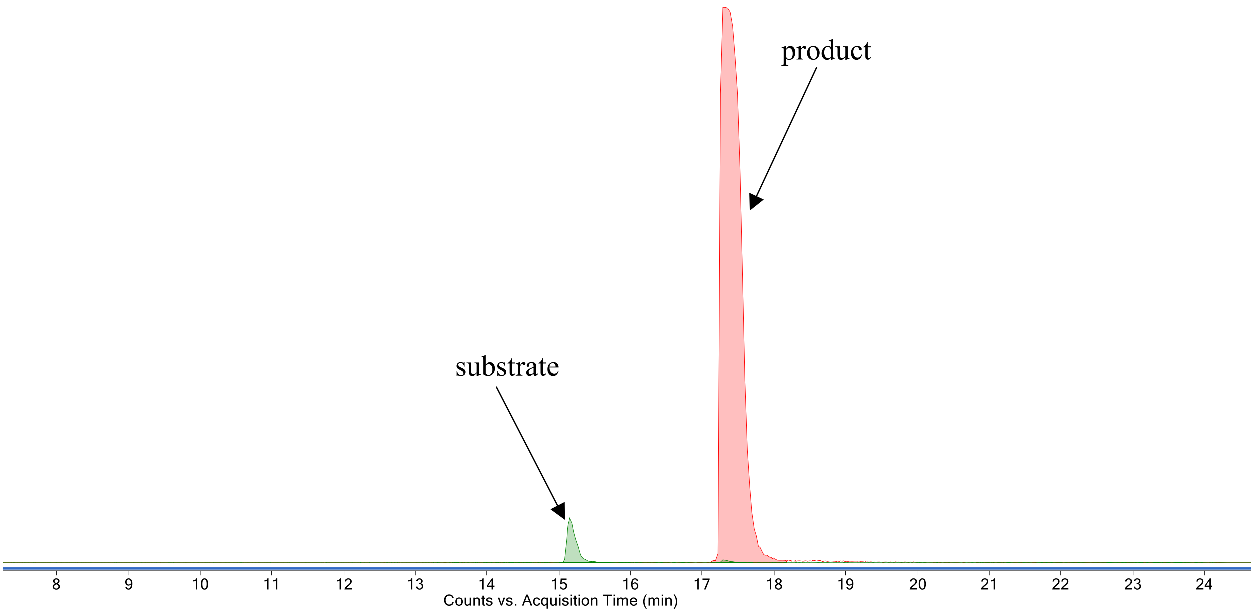

Figure S51: EICs for the most abundant $[M+2H]^{2+}$ ions corresponding to macrocyclic MppE^{core} peptide (in green) and C_{10}-prenylated macrocyclic MppE^{core} peptide (in red) when MppM-modified MppE substrate peptide was incubated with GPP and purified MppC wild-type at 30 °C for 1.5 h.

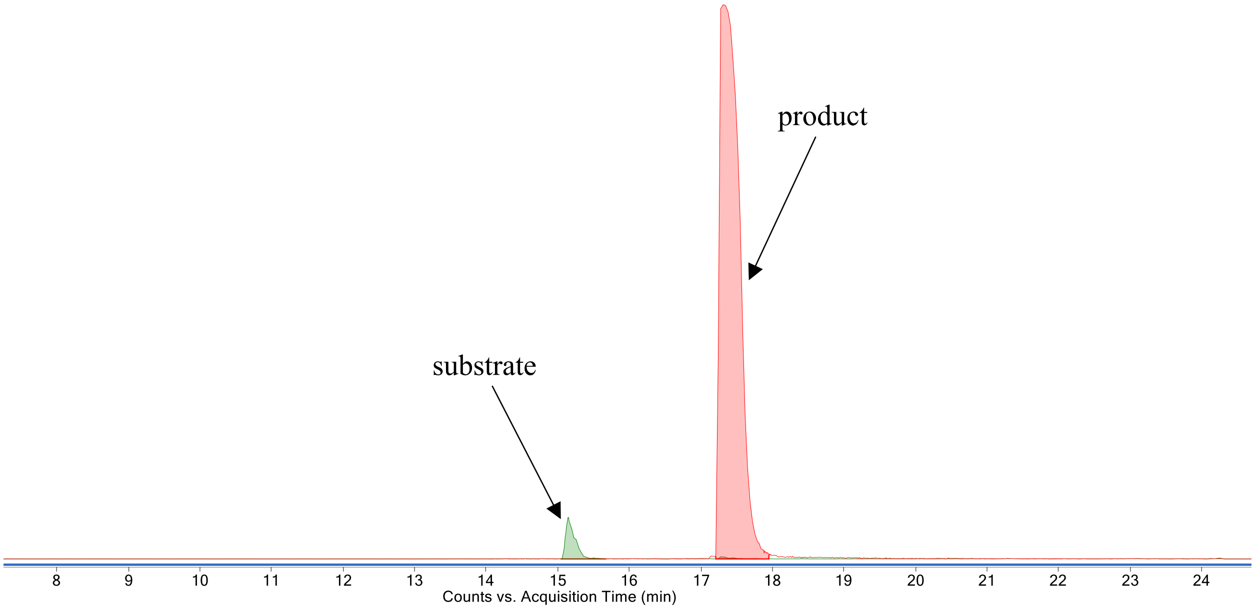

Figure S52: EICs for the most abundant $[M+2H]^{2+}$ ions corresponding to macrocyclic MppE^{core} peptide (in green) and C_{10}-prenylated macrocyclic MppE^{core} peptide (in red) when MppM-modified MppE substrate peptide was incubated with GPP and purified MppC wild-type at 30 °C for 2 h.

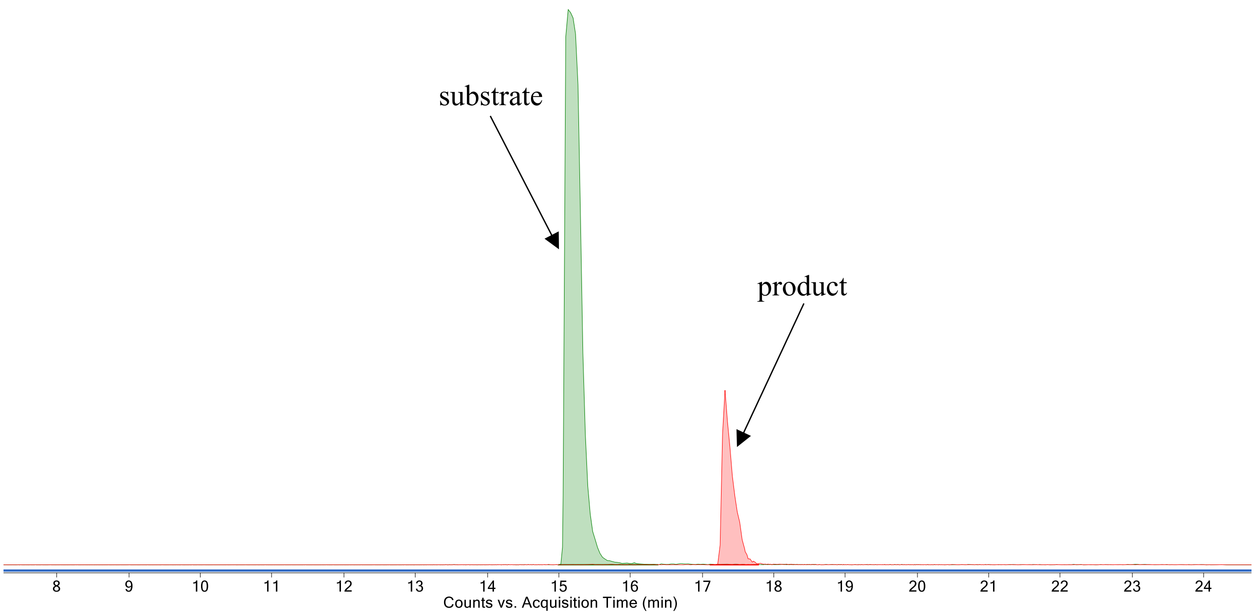

Figure S53: EICs for the most abundant $[M+2H]^{2+}$ ions corresponding to macrocyclic MppE^{core} peptide (in green) and C_{10}-prenylated macrocyclic MppE^{core} peptide (in red) when MppM-modified MppE substrate peptide was incubated with GPP and purified MppC-W336A/F340A at 30 °C for 0.5 h.

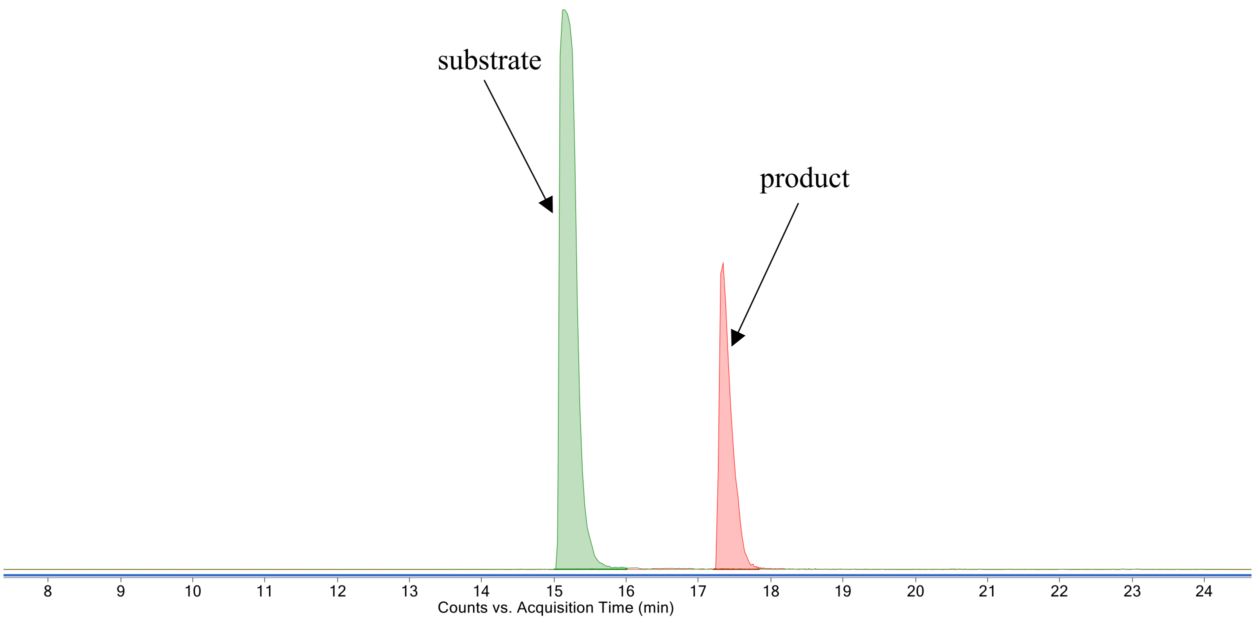

Figure S54: EICs for the most abundant $[M+2H]^{2+}$ ions corresponding to macrocyclic MppE^{core} peptide (in green) and C_{10}-prenylated macrocyclic MppE^{core} peptide (in red) when MppM-modified MppE substrate peptide was incubated with GPP and purified MppC-W336A/F340A at 30 °C for 1 h.

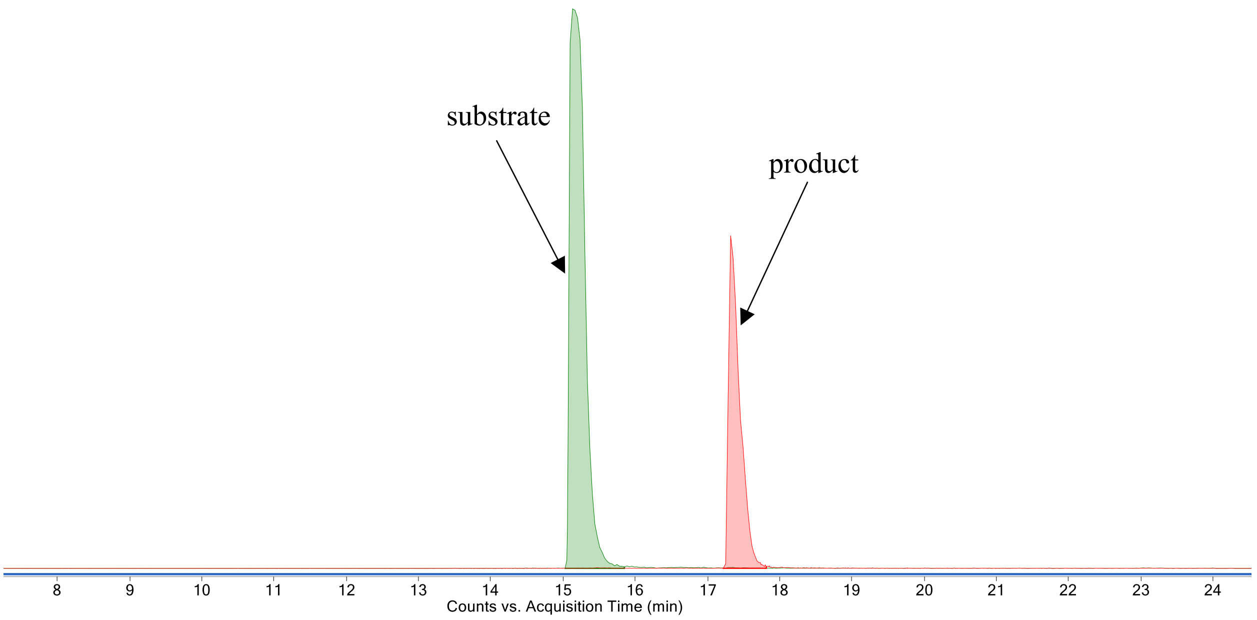

Figure S55: EICs for the most abundant $[M+2H]^{2+}$ ions corresponding to macrocyclic MppE^{core} peptide (in green) and C_{10}-prenylated macrocyclic MppE^{core} peptide (in red) when MppM-modified MppE substrate peptide was incubated with GPP and purified MppC-W336A/F340A at 30 °C for 1.5 h.

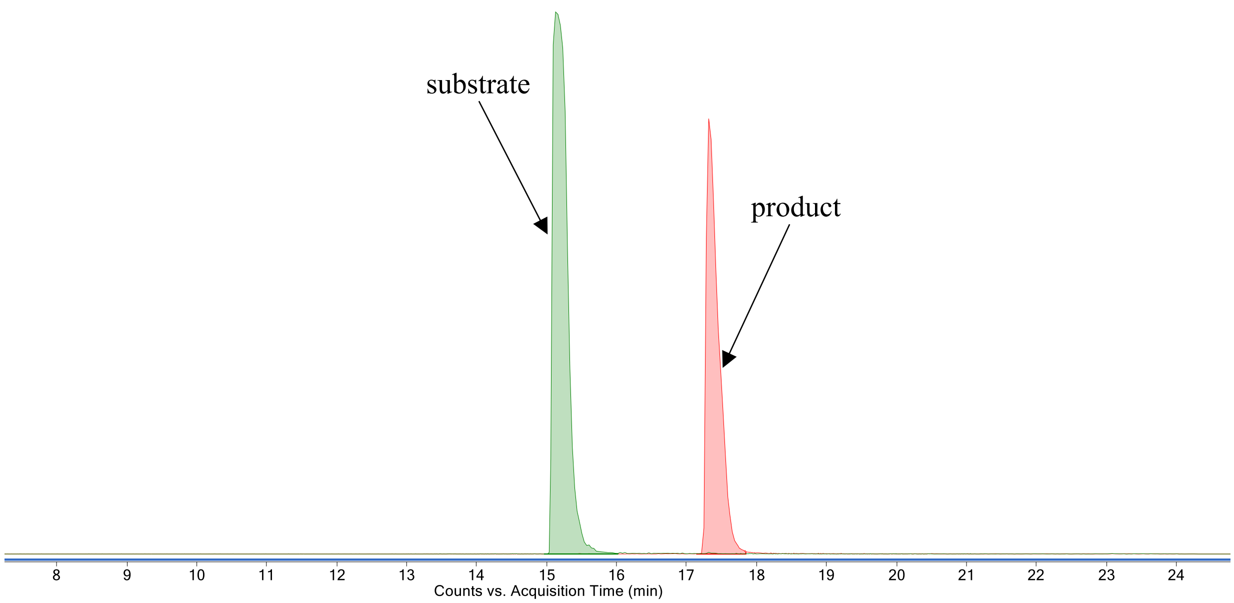

Figure S56: EICs for the most abundant $[M+2H]^{2+}$ ions corresponding to macrocyclic MppE^{core} peptide (in green) and C_{10}-prenylated macrocyclic MppE^{core} peptide (in red) when MppM-modified MppE substrate peptide was incubated with GPP and purified MppC-W336A/F340A at 30 °C for 2 h.

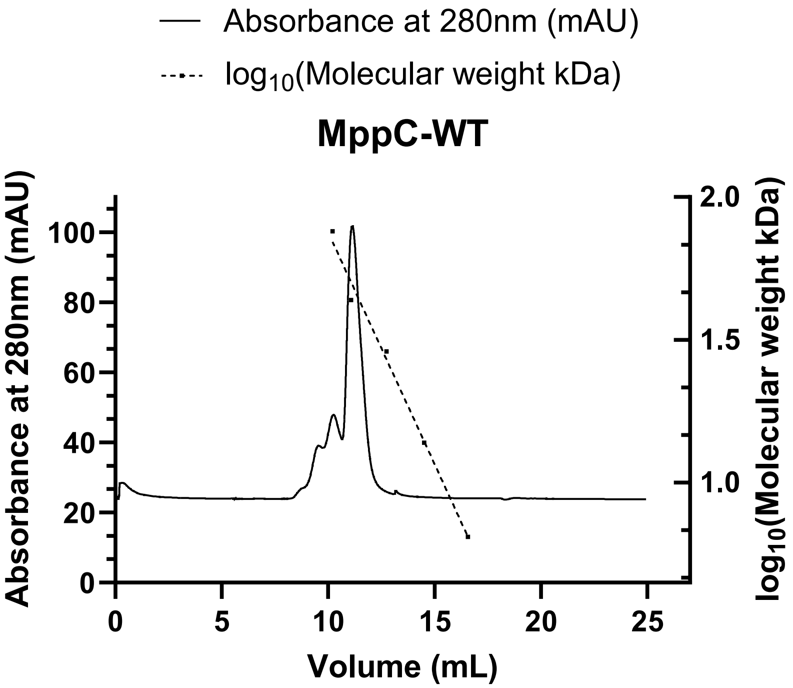

Figure S57: Elution profile for MppC wild-type (solid line) and linear fit of elution volume peaks for standard proteins of known molecular weight (dashed line) on Superdex 75 increase 10/300 GL size exclusion chromatography column (Cytiva). The standard proteins and corresponding molecular weights are: conalbumin, 75 kDa; ovalbumin, 44 kDa; carbonic anhydrase, 29 kDa; ribonuclease A, 13.7 kDa; and aprotinin, 6.5 kDa).

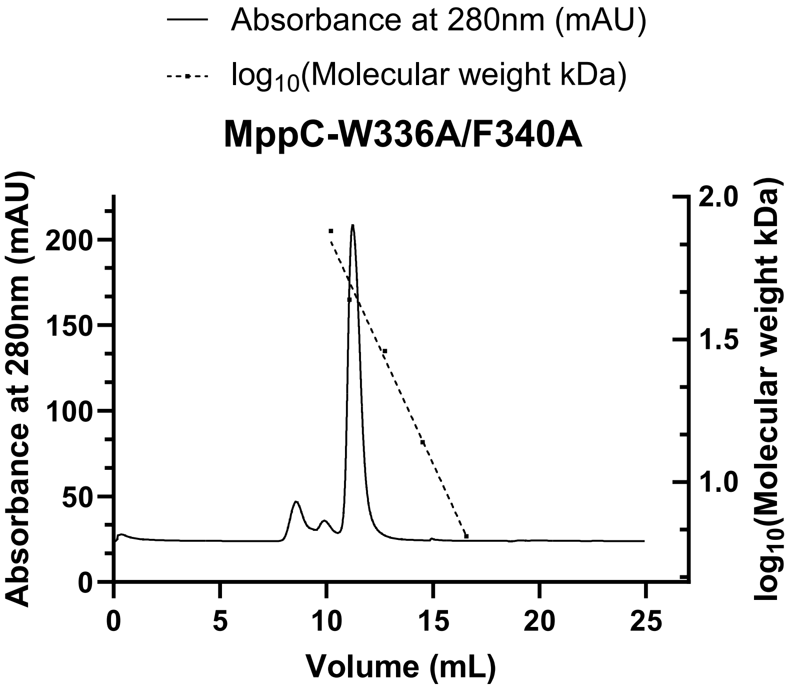

Figure S58: Elution profile for MppC-W336A/F340A (solid line) and linear fit of elution volume peaks for standard proteins of known molecular weight (dashed line) on Superdex 75 increase 10/300 GL size exclusion chromatography column (Cytiva). As compared to the elution profile of the wild-type MppC illustrated in Figure S56, the MppC-W336A/F340A mutant enzyme did not demonstrate increased aggregation or change in oligomeric state.

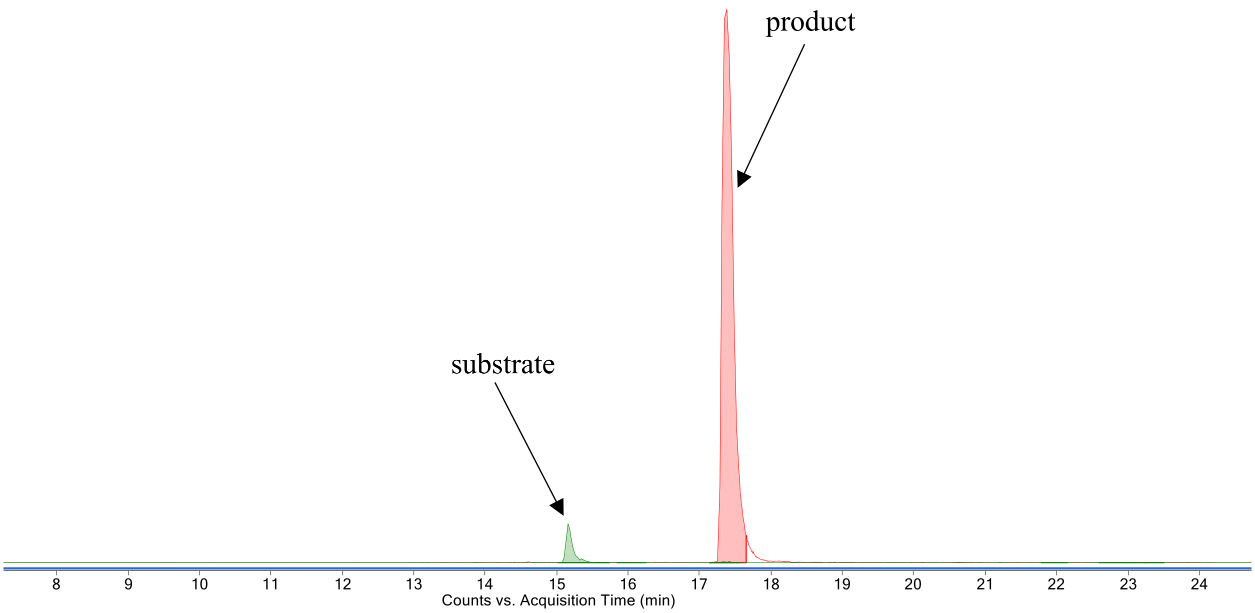

Figure S59: EICs for the most abundant $[M+2H]^{2+}$ ions corresponding to macrocyclic MppE^{core} peptide (in green) and C_{10}-prenylated macrocyclic MppE^{core} peptide (in red) when MppM-modified MppE substrate peptide was incubated with GPP and purified MppC-W336A at 30 °C for 0.5 h.

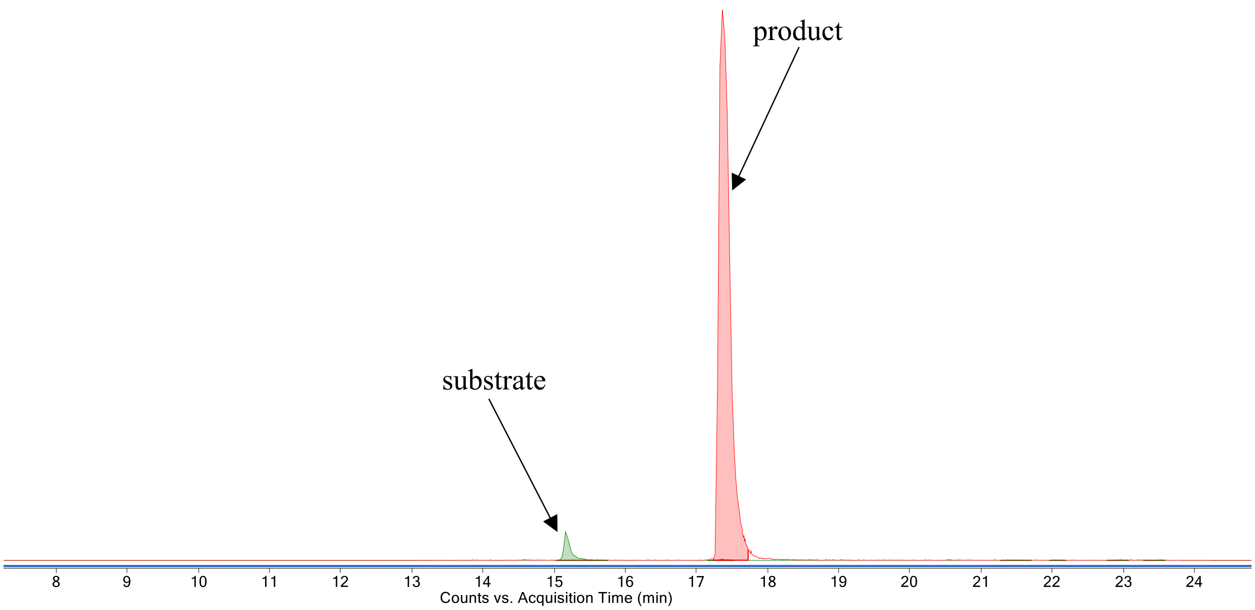

Figure S60: EICs for the most abundant $[M+2H]^{2+}$ ions corresponding to macrocyclic MppE^{core} peptide (in green) and C_{10}-prenylated MppE^{core} peptide (in red) when MppM-modified MppE substrate peptide was incubated with GPP and purified MppC-W336A at 30 °C for 1 h.

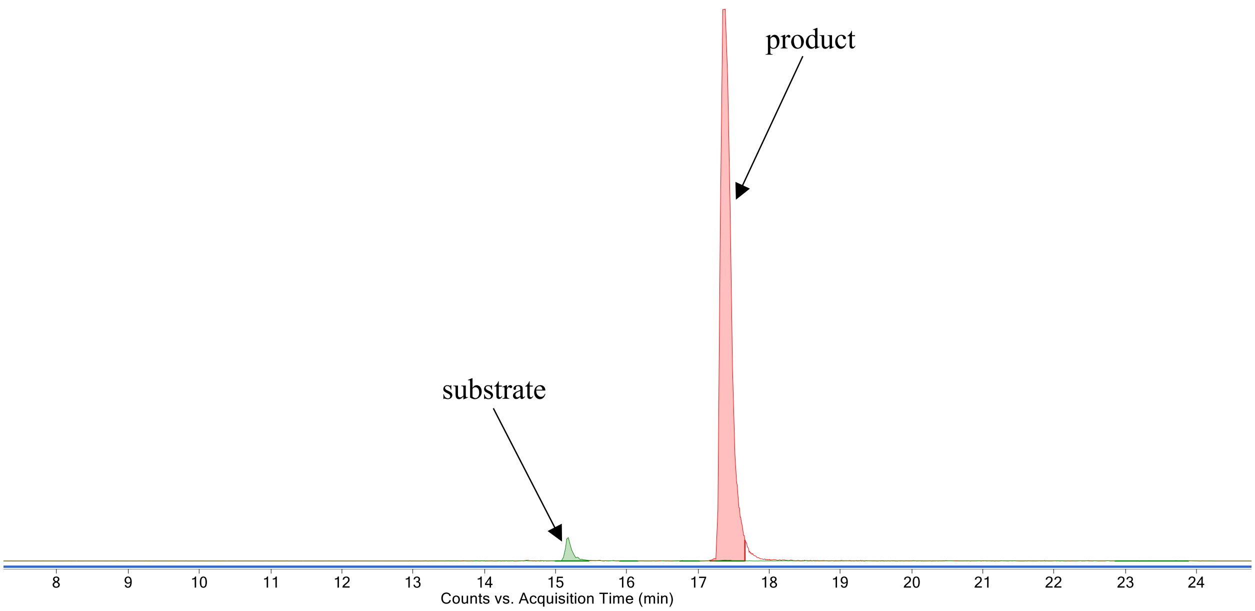

Figure S61: EICs for the most abundant $[M+2H]^{2+}$ ions corresponding to macrocyclic MppE^{core} peptide (in green) and C_{10}-prenylated macrocyclic MppE^{core} peptide (in red) when MppM-modified MppE substrate peptide was incubated with GPP and purified MppC-W336A at 30 °C for 1.5 h.

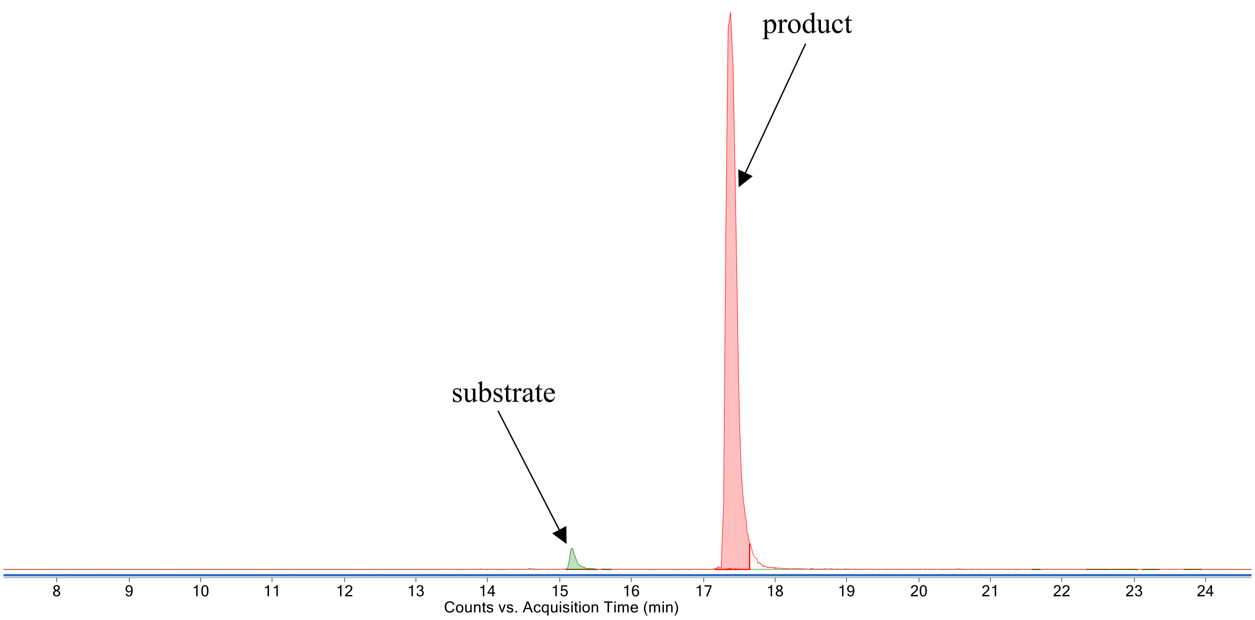

Figure S62: EICs for the most abundant $[M+2H]^{2+}$ ions corresponding to macrocyclic MppE^{core} peptide (in green) and C_{10}-prenylated macrocyclic MppE^{core} peptide (in red) when MppM-modified MppE substrate peptide was incubated with GPP and purified MppC-W336A at 30 °C for 2 h.

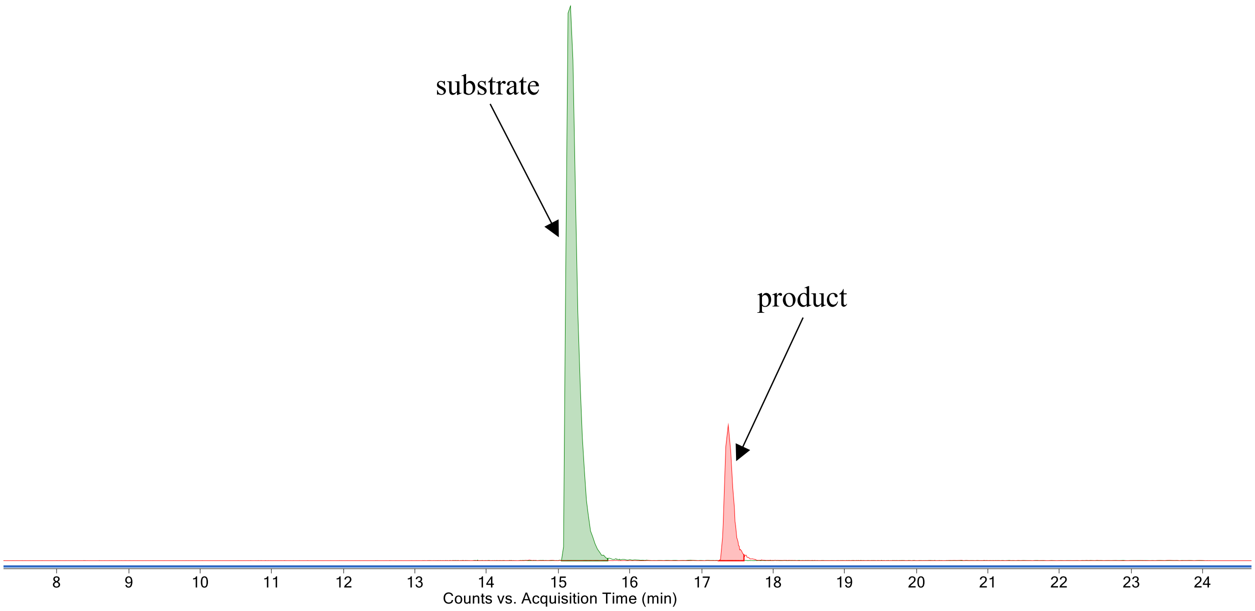

Figure S63: EICs for the most abundant $[M+2H]^{2+}$ ions corresponding to macrocyclic MppE^{core} peptide (in green) and C_{10}-prenylated macrocyclic MppE^{core} peptide (in red) when MppM-modified MppE substrate peptide was incubated with GPP and purified MppC-F340A at 30 °C for 0.5 h.

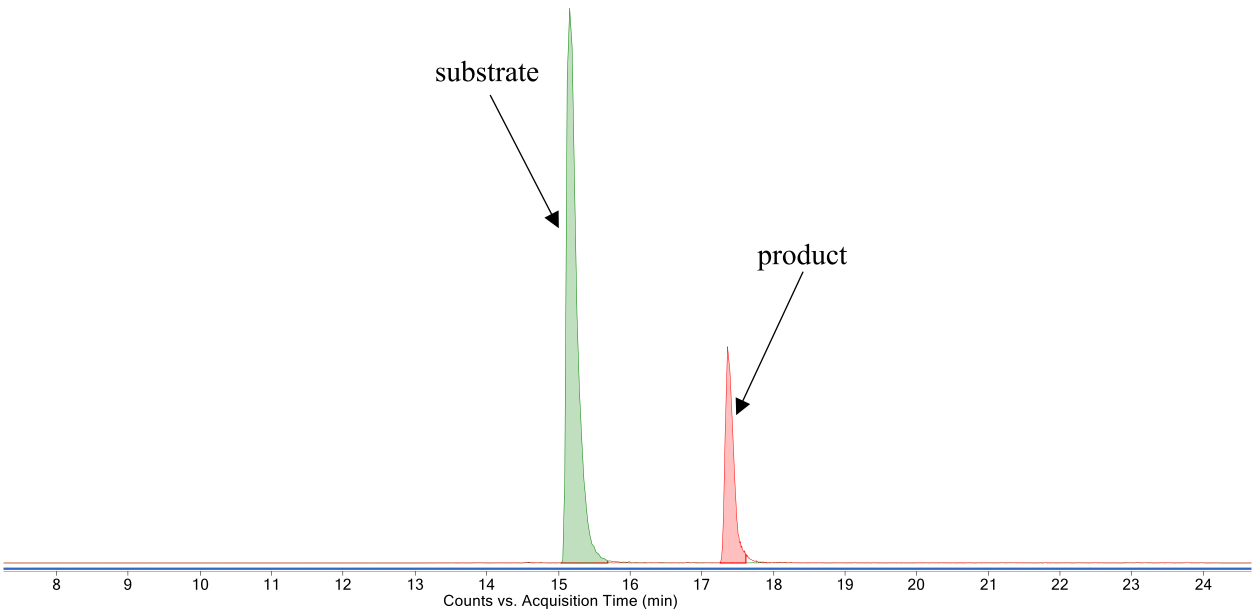

Figure S64: EICs for the most abundant $[M+2H]^{2+}$ ions corresponding to macrocyclic MppE^{core} peptide (in green) and C_{10}-prenylated macrocyclic MppE^{core} peptide (in red) when MppM-modified MppE substrate peptide was incubated with GPP and purified MppC-F340A at 30 °C for 1 h.

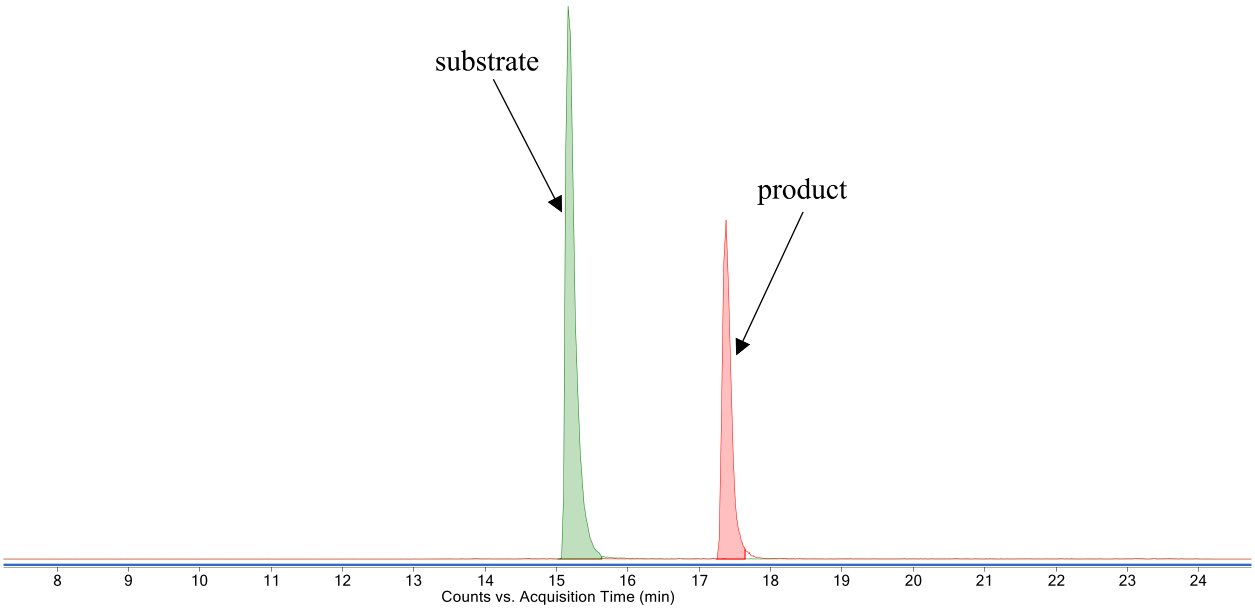

Figure S65: EICs for the most abundant $[M+2H]^{2+}$ ions corresponding to macrocyclic $MppE^{core}$ peptide (in green) and C_{10}-prenylated macrocyclic $MppE^{core}$ peptide (in red) when MppM-modified MppE substrate peptide was incubated with GPP and purified MppC-F340A at 30 °C for 1.5 h.

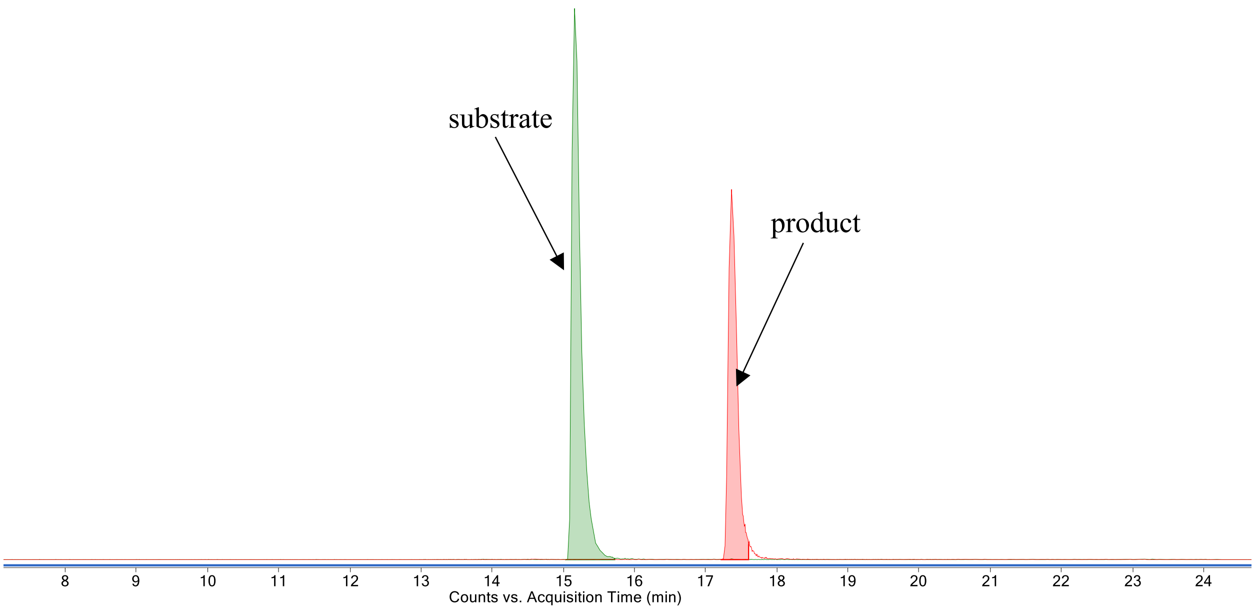

Figure S66: EICs for the most abundant $[M+2H]^{2+}$ ions corresponding to macrocyclic MppE^{core} peptide (in green) and C_{10}-prenylated macrocyclic MppE^{core} peptide (in red) when MppM-modified MppE substrate peptide was incubated with GPP and purified MppC-F340A at 30 °C for 2 h.

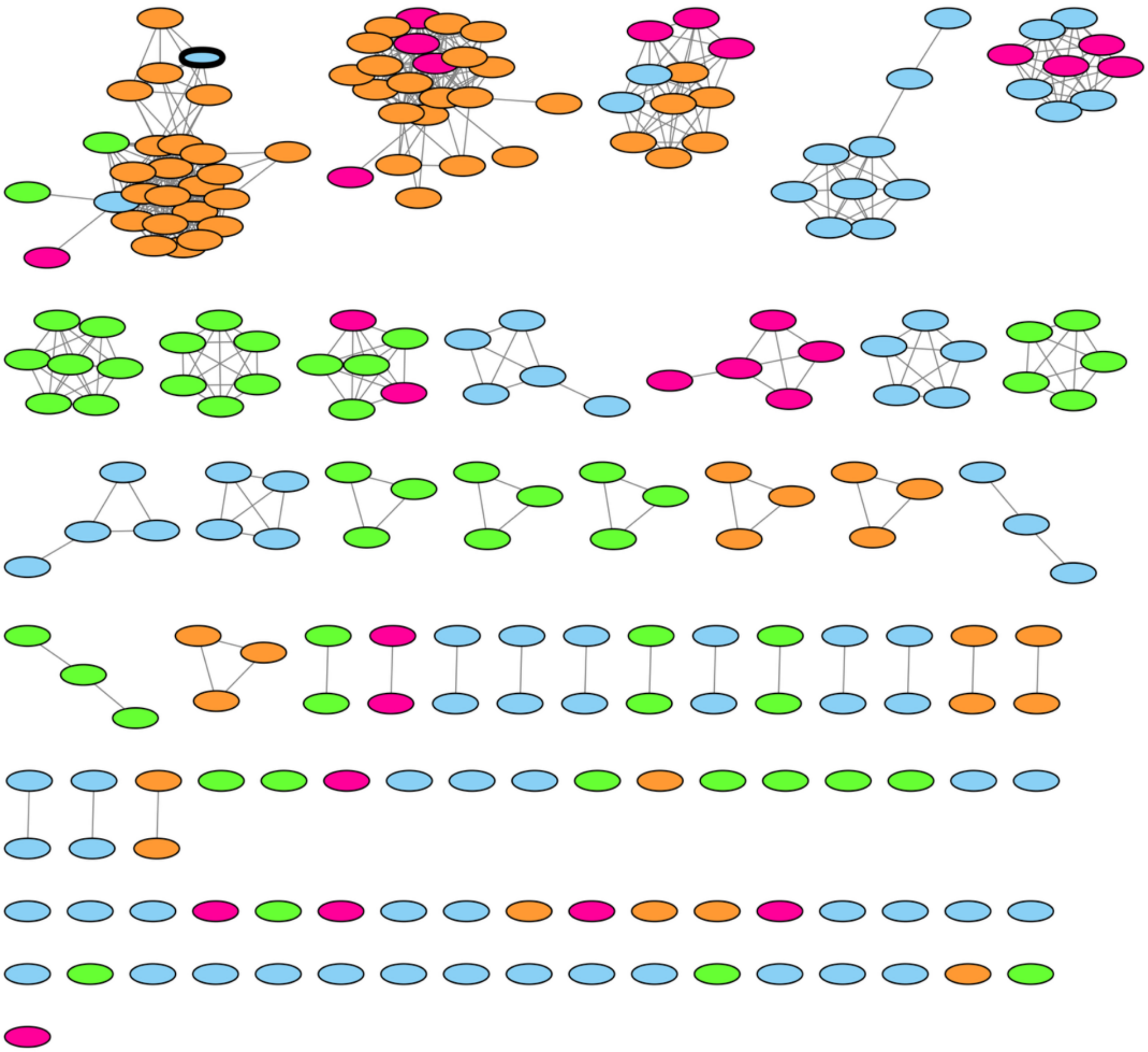

Figure S67: An SSN with 229 nodes demonstrating sequences similar to MppC retrieved from the UniProt database. The nodes are colored according to bacterial phyla with nodes derived from Cyanobacteriota colored orange, nodes derived from Myxococcota colored green, and nodes derived from Pseudomonadota colored pink. All other nodes are colored blue. The node corresponding to MppC is shown with a thick border in the cluster of nodes in the top left corner of the SSN. The sequences that comprise of clusters 1 and 2 (top, left) which denote a majority of Cyanobacteriota-derived sequences co-localize with lanthionine synthetase (LanM) like proteins as denoted by Pfams PF05147-PF13575.

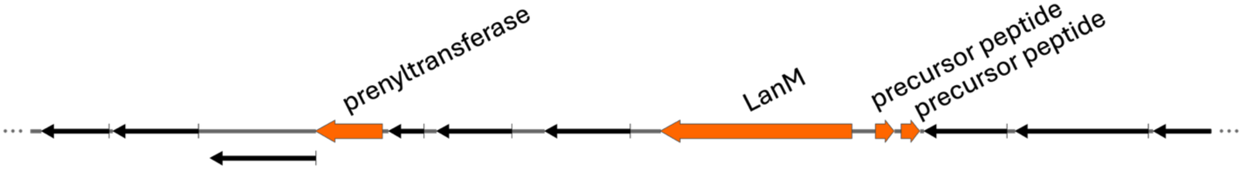

Figure S68: A 20kb region from the genome of the cyanobacterium *Nostocales cyanobacterium* HT-58-2 showing genes encoding two RiPP precursor peptides along with genes encoding a class II lanthipeptide synthetase (LanM) and a prenyltransferase. Other genes with possible roles in RiPP biosynthesis are shown as black arrows. Both precursor peptides possess Nif11-like leader peptides.

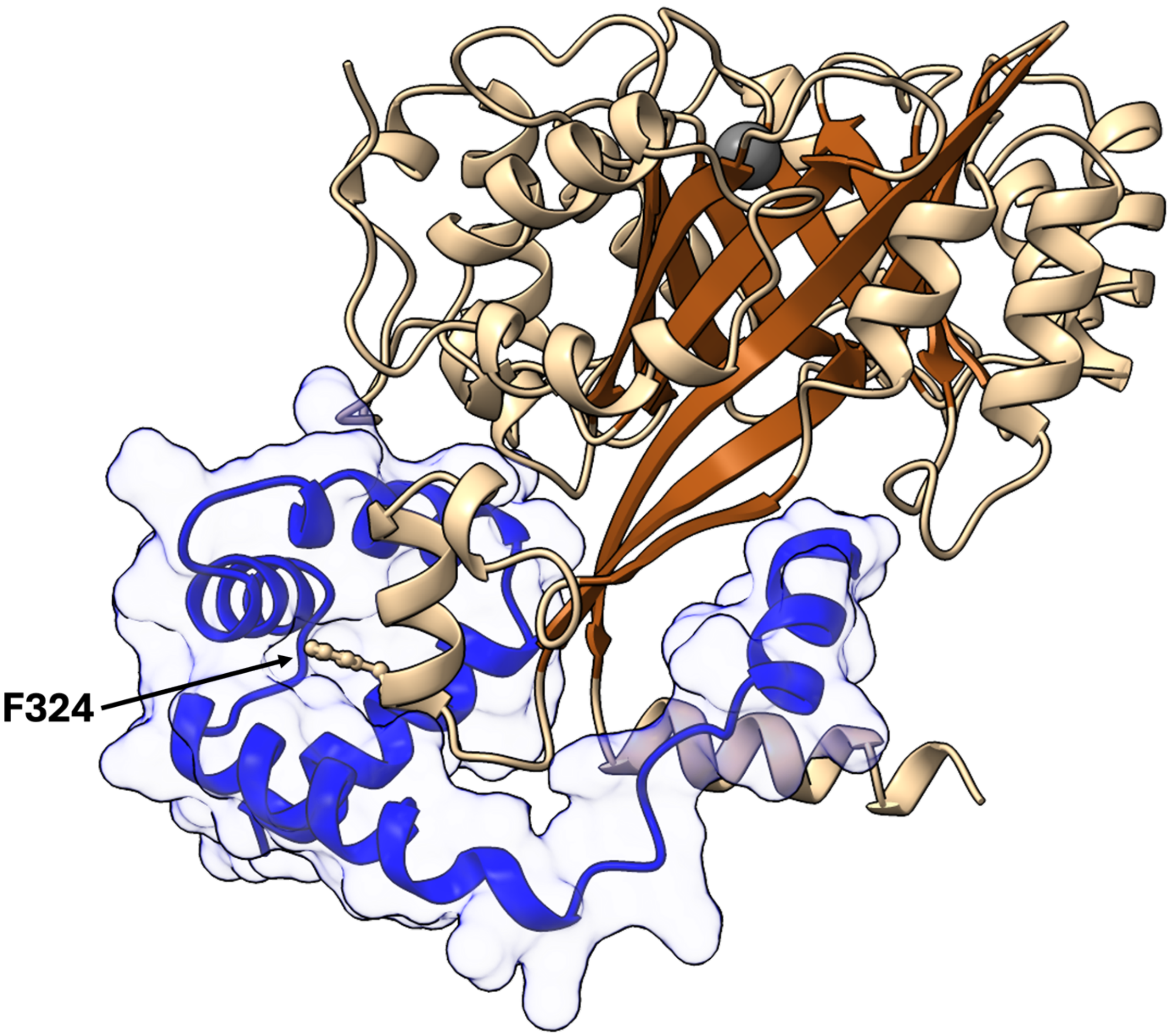

Figure S69: An AlphaFold 3 generated model (ipTM=0.88; pTM=0.92) of the *N. cyanobacterium* HT-58-2 derived prenyltransferase (in brown) in complex with the Nif11-like leader peptide (in blue) described in Figure 66. The Mg^{2+} ion is shown in grey. Note that the side chain of the Phe324 residue is inserted into the hydrophobic cavity of the leader^{SN} akin to the MppC Phe340 residue described in the main text.

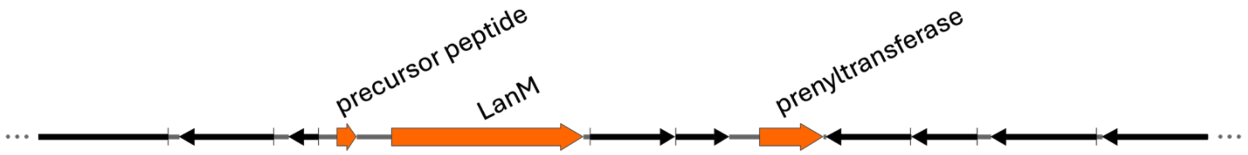

Figure S70: A 20kb region from the genome of the cyanobacterium *Aphanizomenon* sp. UHCC 0183 showing genes encoding a RiPP precursor peptide along with genes encoding a LanM and a prenyltransferase. Other genes with possible roles in RiPP biosynthesis are shown as black arrows. The precursor peptide possess a Nif11-like leader.

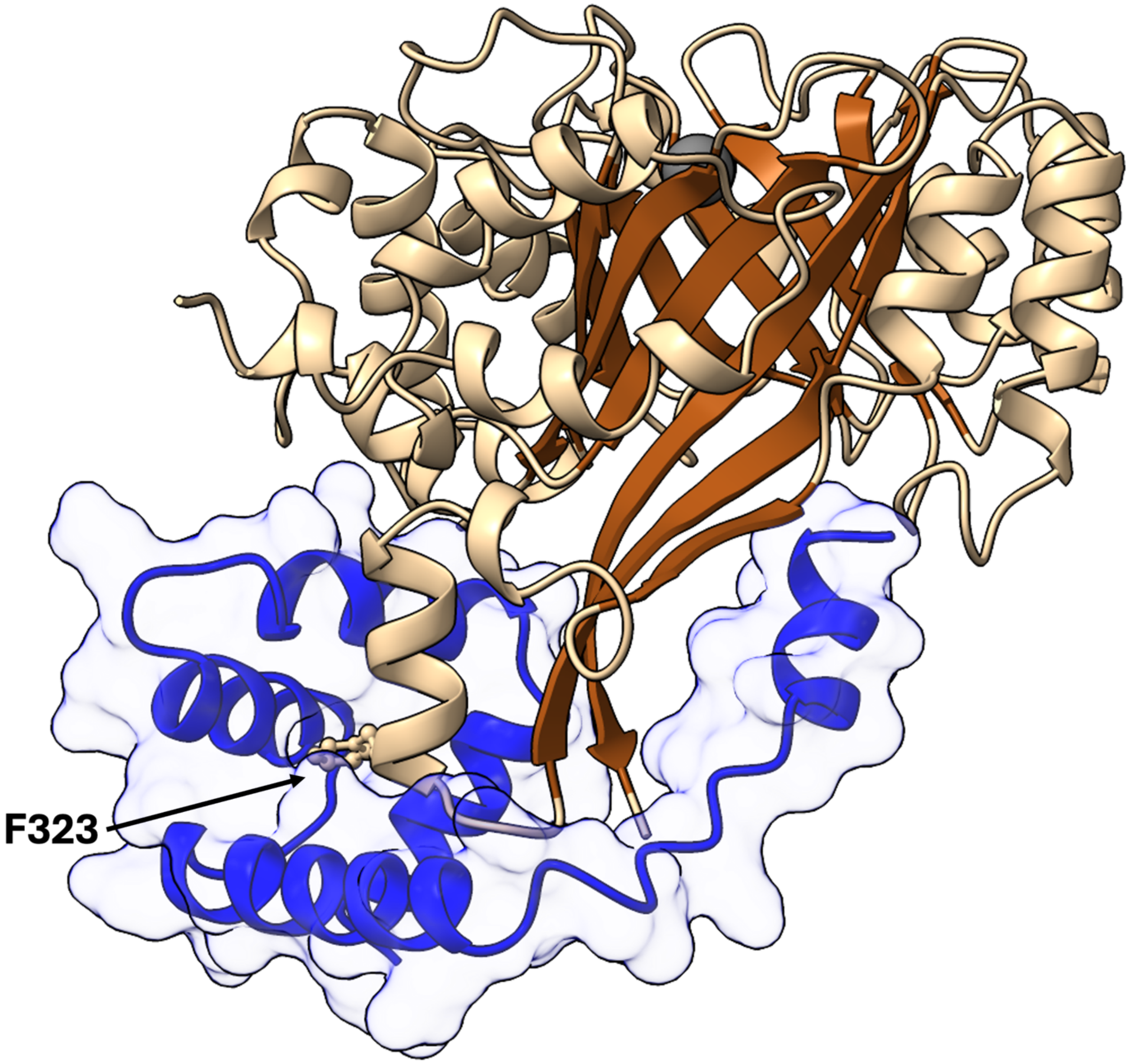

Figure S71: An AlphaFold 3 generated model (ipTM=0.81; pTM=0.90) of the *Aphanizomenon* sp. UHCC 0183 derived prenyltransferase (in brown) in complex with the Nif11-like leader peptide (in blue) described in Figure 68. The Mg^{2+} ion is shown in grey. Note that the side chain of the Phe323 residue is inserted into the hydrophobic cavity of the leader^{SN} akin to the MppC Phe340 residue.

SUPPLEMENTARY REFERENCES

1. Saha, N.; Vidya, F. N. U.; Luo, Y.; van der Donk, W. A.; Agarwal, V., Transformation-Guided Genome Mining Provides Access to Brominated Lanthipeptides. *Org Lett* **2025**.
2. Bobeica, S. C.; Dong, S.-H.; Huo, L.; Mazo, N.; McLaughlin, M. I.; Jiménez-Osés, G.; Nair, S. K.; van der Donk, W. A., Insights into AMS/PCAT transporters from biochemical and structural characterization of a double Glycine motif protease. *eLife* **2019,** *8*, e42305.
3. Zallot, R.; Oberg, N.; Gerlt, J. A., The EFI web resource for genomic enzymology tools: leveraging protein, genome, and metagenome databases to discover novel enzymes and metabolic pathways. *Biochemistry* **2019,** *58*, 4169-4182.
